# Reassessing neolithic subsistence in Northern Italy through a critical review and new evidence from Molino Casarotto

**DOI:** 10.1038/s41598-025-28005-6

**Published:** 2025-11-28

**Authors:** Francesco Breglia, Vito Giuseppe Prillo, Marta Dal Corso, Maria Sofia Manfrin, Silvia D’Aquino, Federico Polisca, Luigi Germinario, Giorgio Piazzalunga, Paola Salzani, Cristiano Nicosia

**Affiliations:** 1 Dipartimento di Geoscienze, Università di Padova, 35131 Padua, Italy; 2 Dipartimento di Beni Culturali, Università di Padova, 35139 Padua, Italy; 3 Dipartimento di Beni Culturali e Ambientali, Laboratorio di Preistoria, Protostoria ed Ecologia Preistorica, Università degli Studi di Milano, 20141 Milan, Italy; 4 Soprintendenza Archeologia, Belle Arti e Paesaggio per le province di Verona, Rovigo e Vicenza, 37121 Verona, Italy; 5 Dipartimento di Beni Culturali, Università del Salento, 73100 Lecce, Italy

**Keywords:** Neolithic, Subsistence strategy, Foraging, Squared-Mouthed pottery, Wild plants, fishing, Environmental economics, Plant domestication, Wetlands ecology

## Abstract

This paper delves into the intricate nature of neolithic subsistence economies in northern Italy by providing a comprehensive overview of all previously edited data, quantitatively compared, including plant macroremains, faunal remains, and aquatic resources. Additionally, it introduces new findings from the site of Molino Casarotto, recently investigated as part of the ERC CoG GEODAP project, through an interdisciplinary approach that combines carpological analysis, phytolith analysis, and archaeozoological studies of both terrestrial and aquatic fauna. This wetland settlement, with material culture fully representative of the middle neolithic Square-Mouthed Pottery (SMP) culture, showcases an economy primarily based on hunting, fishing, gathering molluscs and wild fruits, with water chestnut serving as the staple food. The rare presence of bone remains from domestic animals and a few cereal grains indicates access to food from a agro-pastoral economy. However, the absence of chaff remains, confirmed by phytolith analysis, suggests that cereal cultivation and processing did not occur on site. These findings, within the broader context of the cultural and environmental diversity of the northern Italian Neolithic, challenge the rigidity of concepts often used to describe Neolithization processes. The emerging picture is non-linear and complex, both chronologically and geographically, with local peculiarities that push to abandon a dichotomous view of foraging versus agricultural economy.

**Supplementary Information:**

The online version contains supplementary material available at 10.1038/s41598-025-28005-6.

## Introduction

Since its 19th-century definition^1^, the term Neolithic has expanded from a pure technological to a broader cultural, social, and economic concept. Classical notions such as the “Neolithic revolution” and “Neolithic package” describe the transition to farming^2–5^, herding^6^, and new material markers, though since the 1980s they have been increasingly debated^2,3,7–17^. Critics argue that the package model oversimplifies the process, emphasizing instead the variability of subsistence strategies shaped by both environmental and cultural factors across time and space^16^.

Although paleogenetic research has shown that the diffusion of neolithic lifeways in Europe was caused by migrations of human groups from Anatolia and the West Asia, in some regions incoming farmers encountered indigenous hunter-gatherers, leading to different neolithization models^18–29^. Early research, mainly based on archaeozoological evidence highlighting the importance of hunting, hypothesized that sub-Alpine Italy may have been one of these regions and interpreting Neolithization of northern Italy in terms of an admixture of diffusionist and acculturation models^30–34^. Later studies—based on material culture and radiocarbon dating—have questioned the extent of such interactions, except in the Adige Valley, where stratigraphic evidence may support them^35^. Besides a possible Mesolithic substrate, the Neolithic of the region was shaped by its geography. Enclosed by the Alps, Apennines, and Adriatic Sea, the forested plains of northern Italy with their hydrographic network^36–41^, served as a crossroads between the Mediterranean and Central Europe (north–south) and between the Balkans and southern France (east–west). In the 6th millennium BCE, at least three diffusion routes reached the region: a western one through Liguria, a southern along the Adriatic via the Romagna plain, and an eastern through the Balkans into Friuli. These brought diverse traditions that shaped a cultural mosaic, represented by the Ligurian Cardial Pottery, Fiorano, and Friulian groups^42,43^. By the late 6th millennium BCE, new groups appeared—Vhò in the central Po Plain, Isolino in the Varese Prealps, and Gaban in the Adige Valley. From the outset, this mosaic was dynamic, as pottery and lithic circulation indicate strong interaction, especially in border areas, supported by medium- and long-distance exchange networks^44–48^. Over the past decade, scholars have argued that these dynamics gave rise to the Square-Mouthed Pottery (SMP) culture, emerging from Early Neolithic traditions through the gradual integration of local communities into a new syncretic framework^49–51^. This process, beginning around 5000 BCE and culminating by 4700 BCE, marked the onset of the middle Neolithic, characterized by cultural homogeneity in northern Italy. The SMP sequence spans the 5th millennium BCE, with three internal styles grouped into two main stages: stabilization (SMP I–II) until mid-millennium, and expansion (SMP III) until about 4000 BCE^52^. This expansion is especially evident in the north-eastern area, where SMP spread northward and eastward. Settlements now include plains, hills, lakesides, and high-altitude sites, as well as open-air locations, caves, and rock shelters, reflecting a full territorial “fill-in,” broad environmental adaptation, and likely diversification of resource use^52^. At the same time, processes of cultural fragmentation marked the late Neolithic in other areas. In north-western Italy, new elements from southern France gradually replaced SMP II traditions. The Chassey culture spread eastward, merging with local SMP elements in western Lombardy to form a ‘Protolagozza’ phase, later developing into the Lagozza culture^51,53^. In Emilia, Chassey and SMP sites coexisted around 4300–4200 BCE, with limited direct contact and potential tensions, before SMP traits eventually disappeared. Further southeast, in the Romagna plain, communities associated with peninsular cultures (i.e. Ripoli and Diana) established a broad territorial presence, later incorporating Chassey-Lagozza influences, reflecting the growing spread of western traditions^54^. By the end of the 5th millennium the earlier cultural framework was largely displaced. Even in the north-east, the first half of the 4th millennium BCE saw demographic contraction and the dissolution of the SMP horizon, with poorly standardized ceramics retaining only residual SMP traits, strong north-Alpine and eastern influences emerging, and settlement patterns focusing on lakesides, hills, and continued high-altitude occupation, possibly reflecting economic changes^52^.

## State of knowledge on neolithic subsistence

Although knowledge of Neolithic subsistence has grown, comprehensive reconstructions remain limited by scarce, non-systematic archaeobotanical and archaeozoological analyses, compounded by taphonomic constraints and variable sampling methods. Sampling strategies critically affect the quality and representativeness of faunal and botanical assemblages. Systematic recovery through sieving or flotation, calibrated to stratigraphic units, produces more reliable data^55^, yet this approach was often neglected in older excavations, where hand-collection of visible remains prevailed. As a result, comparisons between sites excavated in different periods and by different research groups cannot be fully standardized. These biases may underrepresent certain taxa (e.g., fish bones, small seeds) or activities (e.g., fishing, mollusc gathering), so reconstructions should be regarded as partial and open to refinement as more systematically sampled datasets become available. Although some regional reviews have been produced, these rarely present quantitative data and generally treat agriculture^39,56,57^ and husbandry^58,59^ separately, neglecting aquatic resources and not presenting an overall view. Generally speaking, early archaeobotanical studies suggested a gradual introduction of cultivated crops^60^, while zooarchaeological evidence pointed to the continued importance of wild species, including red and roe deer and wild boar^61–70^. As already mentioned this combination of domestic and wild resources was initially interpreted as reflecting acculturation processes^31,33^. However, more recent research has shown that all the main crops—hulled wheats, naked wheats, barley, lentils, peas, and bitter vetch—were present from the earliest phases^39,56,57^. Nonetheless, the collection of wild fruits, especially hazelnuts, remained economically significant throughout the early and middle Neolithic as also seen in other part of Europe^71–76^, while hunting continued to play a variable role, possibly related to the local forest coverage^77^.

**Fig. 1:**
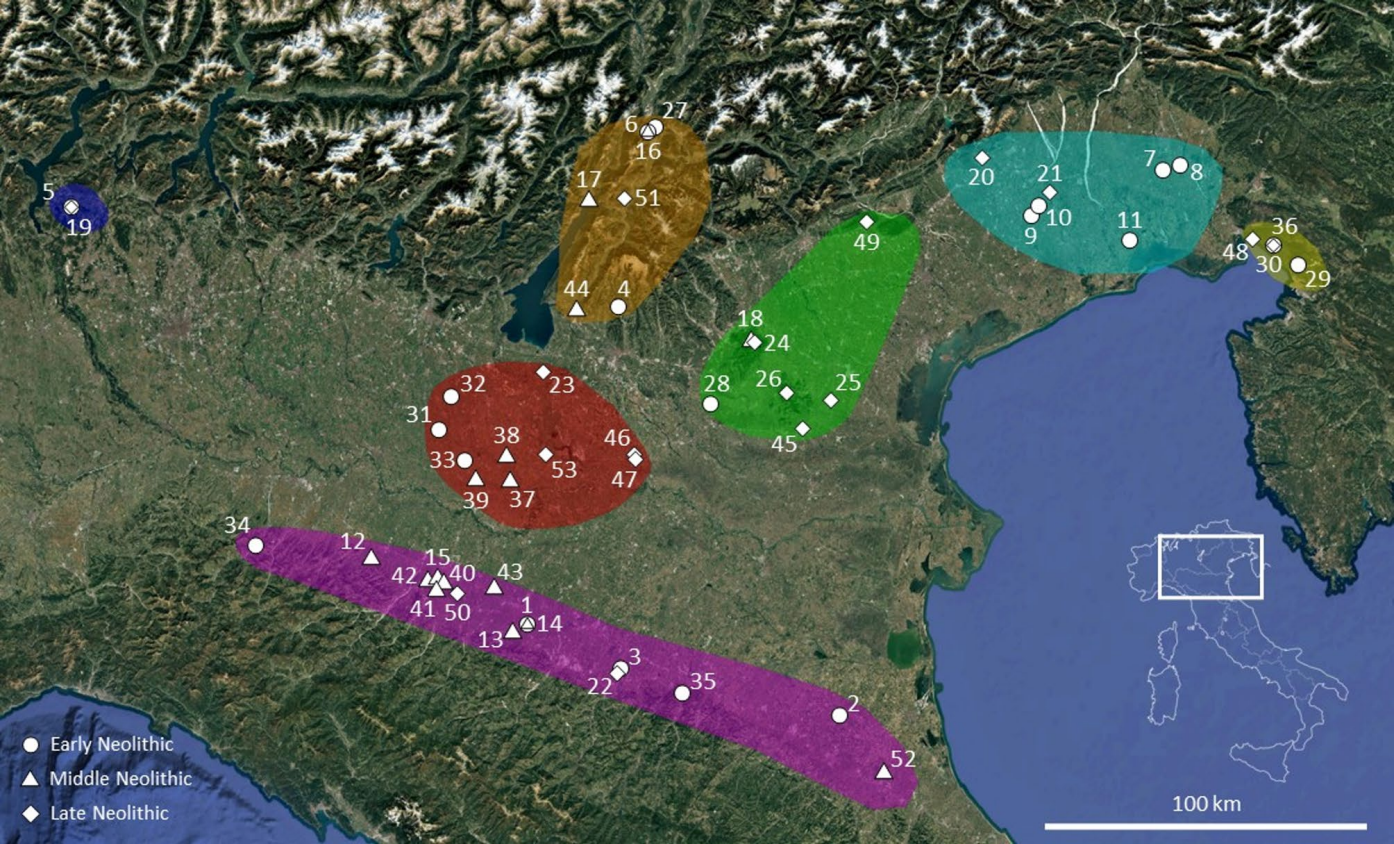
Map of the study area in northern Italy showing the location of the 53 Neolithic sites analyzed, grouped into seven sub-regions and color-coded as follows: Trieste Karst (yellow: 29. Grotta degli Zingari, 30. Grotta dell’Edera—Phase 1, 36. Grotta dell’Edera—Phase 2, 48. Grotta del Mitreo—Trincea 5); Friulian Plains (cyan: 7. Sammardenchia, 8. Pavia di Udine, 9. Fagnigola, 10. Valer, 11. Piancada, 20. Palù di Livenza, 21. Bannia—Palazzine di Sopra); Venetian Hills and Plains (green: 18. Fimon—Molino Casarotto, 24. Fimon—Le Fratte, 25. Maserà di Padova, 26. Castelnuovo di Teolo, 28. Cologna Veneta, 45. Monselice, 49. Cornuda); Garda-Venetian Prealps and Adige Valley (orange: 4. Lugo di Grezzana, 6. La Vela—Phase 1, 16. La Vela—Phase 2, 17. Riva del Garda—Via Brione, 27. Riparo Gaban, 44. Rocca di Rivoli, 51. Isera—La Torretta); Central Po Plain (red: 23. Tosina di Monzambano, 31. Ostiano—Dugali Alti, 32. Isorella, 33. Vhò di Piadena, 37. Belforte di Gazzuolo, 38. Casatico di Marcaria, 39. Rivarolo Mantovano, 46. Olmo di Nogara, 47. Gazzo Veronese—Scolo Gelmina, 53. Levata di Curtatone); Southern Po Plain (magenta: 1. Bazzarola—Phase 1, 2. Lugo di Romagna, 3. Spilamberto—Via Macchioni, 12. Ponte Ghiara, 13. Rivaltella—Cà Romensini, 14. Bazzarola—Phase 2, 15. Parma—Via Guidorossi, 22. Spilamberto—Site VIII, 34. Casa Gazza, 35. Casalecchio di Reno, 40. Parma—Benefizio, 41. Gaione—Parco del Cinghio, 42. Vicofertile, 43. Razza di Campegine, 50. Botteghino, 52. Forlì—Via Navicella); and Varese Prealps (blue: 5. Isolino Virginia—Phase 1, 19. Isolino Virginia—Phase 2). Each site is also annotated with a symbol indicating its chronological phase (Early, Middle, or Late Neolithic). Map modified from Google Earth (© Google, 2025). Used for academic research purposes.

**Fig. 2:**
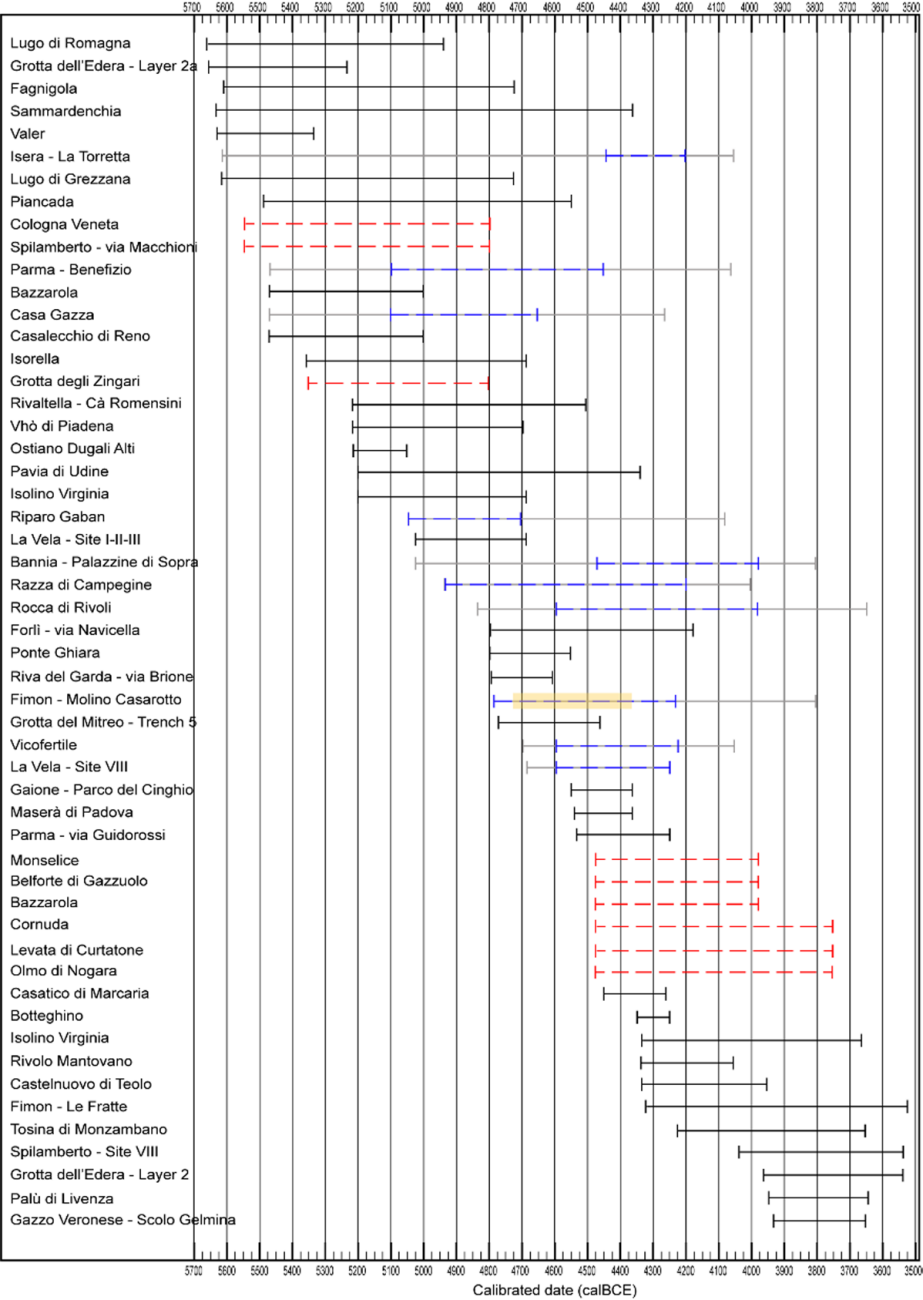
Chronological spans of Neolithic archaeological sites in north-eastern Italy with available zooarchaeological and/or carpological analyses referenced in this paper. The time intervals shown for each site correspond to the radiocarbon dates listed in SI Table S1. The solid black line indicates the full chronological interval between the oldest and the most recent radiocarbon dates available. Where possible, radiocarbon dates were selected to refer specifically to the archaeological layers from which the faunal and carpological assemblages were recovered. Grey lines indicate cases in which the full radiocarbon interval could be narrowed thanks to the attribution of a site to an archaeological culture (blue dashed lines). Red dashed lines indicate sites for which no radiocarbon dates are available and chronological reference is based solely on cultural attribution. Absolute dates for archaeological cultures follow Pessina and Tiné^77^. For Fimon—Molino Casarotto, a yellow box indicates the chronological interval based on radiocarbon dates from the 2022 excavation from which the studied assemblages come from.

Within this framework, the middle Neolithic site of Molino Casarotto plays a crucial role in understanding the socio-economic dynamics of the Neolithic in northern Italy. Known since the late 1960s, and alongside sites in the Adige Valley, Molino Casarotto was initially central to hypotheses of acculturation models, as early archaeologists interpreted the site’s economy as primarily based on hunting, fishing, and gathering^78^. Recent excavations, however, have provided new data through systematic sediment sampling, including flotation and sieving, ensuring that no archaeobotanical or faunal evidence was lost, either qualitatively or quantitatively. This rigorous investigation did not radically alter the overall reconstruction of the site’s economy, leaving open the question of why a site with a predominantly foraging-oriented economy existed during the middle Neolithic, approximately 1000 years after the last Mesolithic occupations in the region. To understand and interpret this evidence within the broader Neolithic dynamics of northern Italy, we not only present the results from Molino Casarotto but also a complete quantitative review of all available carpological and faunal data from Neolithic sites in northern Italy, excluding its westernmost part (Figs. 1, 2). This review is based on all variables related to plant and animal subsistence, including fish and mollusc exploitation, and evaluates potential chronological and geographical patterns while remaining attentive to the nuances and peculiarities that might fall outside broader trends.

## Molino Casarotto: old data and new excavations

The Neolithic site of Molino Casarotto is located in the Fimon valley, within the Berici Hills district (Veneto, north-eastern Italy). The site was first reported in 1943 during extensive peat extraction activities that had begun in the late 19th century and intensified during the Second World War. These operations brought to light a variety of archaeological materials and structural remains, among them a distinctive elliptical hearth made of stone resting on wooden planks^79^. This feature was later precisely relocated during the systematic excavations conducted between 1969 and 1972 by Barfield and Broglio, which established Molino Casarotto as one of the earliest lakeside settlements in northern Italy^78^. The investigations of 1969–1972 covered about 450 m² and revealed three distinct habitation areas, each characterized by substantial hearths, layers of ash, and accumulations of burnt mollusk shells and domestic refuse, interpreted as shell middens^80^. These were built directly above wooden platforms composed of horizontally arranged beams and posts, interpreted either as house foundations or as reclamation structures designed to stabilize the lacustrine margins^78,81,82^. While the first dwelling area was explored in its entirety, the second and third were only partially excavated. Ceramic assemblages were classified within the SMP culture, while a rich lithic industry, primarily based on high-quality flint from the Monti Lessini and greenstones from south-western Lombardy, testified to long-distance procurement networks^46^. One of the distinctive aspects of the earlier research was the analysis of plant and animal remains, which provided a preliminary outline of the subsistence economy^62,68^. Zooarchaeological studies emphasized the predominance of wild animals in the faunal assemblage, especially red deer and wild boar, suggesting that hunting played a central role in the community’s diet. Fish bones were also recorded, with pike identified among the species, together with shells of freshwater mussels (Unio sp.). Mollusc remains were abundant, though their quantity was not systematically quantified, as well as that of the plant remains^61,78^, and one of the main hearths was described as containing “alternate layers of freshwater mussel shells and water-chestnut shells”^62^. Alongside the predominance of water chestnut, plant macro-remains included abundant hazelnuts and some wild grape pips, while cereals were very rarely attested. These data led to an interpretation of Molino Casarotto’s economy as a distinctive adaptation to the wetland environment of the Fimon valley. Because of the very limited presence of cereals and domestic animals, the settlement was originally described as practicing “a basically Mesolithic economy of hunting and gathering by people with a Neolithic material culture”^62^. This formulation encapsulated the double hypothesis proposed at the time: either that Neolithic settlers had adapted their economy to the particular ecological conditions of the valley, or that indigenous Mesolithic groups had adopted selected elements of Neolithic technology and material culture.

After several decades, new fieldwork was initiated. Preliminary geophysical surveys carried out in 2020 indicated the presence of anomalies corresponding to the hearth previously excavated by Barfield and Broglio in the second dwelling area. These results led to a new excavation in 2022, within the framework of the ERC-CoG GEODAP (Geoarchaeology of Daily Practices) project, with the goal of re-examining Molino Casarotto. The new campaign reopened and extended the second habitation area explored in the 1970s, allowing for the first time a clearer definition of the structural boundaries of the settlement. A line of wooden posts uncovered at the northern edge of the excavation has been interpreted as a palisade or delimitation element, possibly separating the habitation zone from the external space of the valley floor. This discovery contributes to the understanding of the spatial organization of the settlement^79^.

**Fig. 3:**
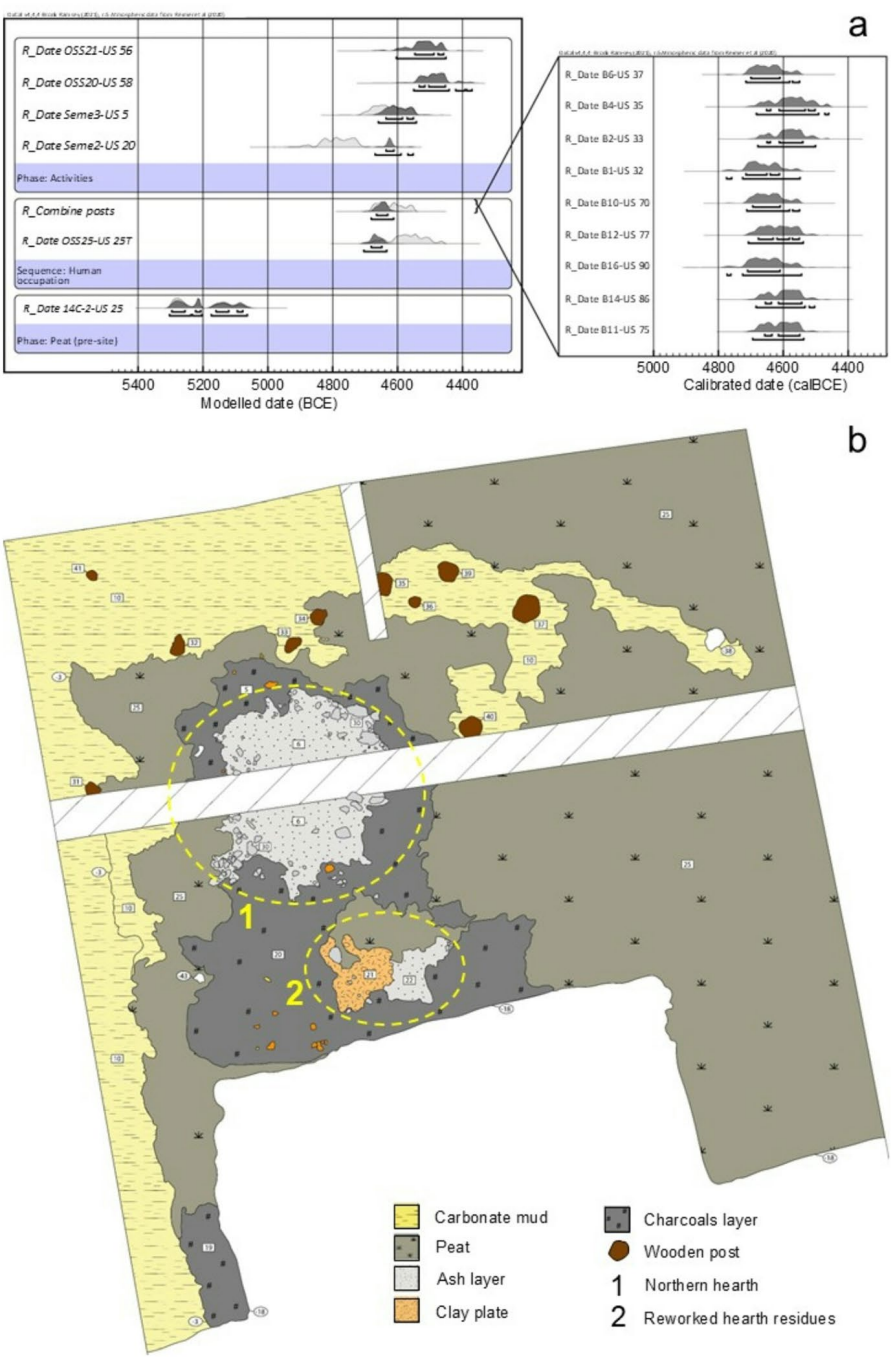
**a** Plot with the dates from the 2022 campaign modelled. On the right are all the dates of the posts to the north, which in the plot on the left have been ‘combined’ together using the R_combine function of Oxcal; **b** plan of the 2022 campaign with an indication of the main features discussed in this article. Map created using Adobe Illustrator (v. 25.2.1—Adobe Inc.; https://www.adobe.com/products/illustrator.html).

Excavations allowed researchers to determine that the site was built some centuries after the withdrawal of Lake Fimon and the subsequent establishment of a terrestrial peat-covered environment dating to 5306–5068 cal BCE. The new set of radiocarbon dates and the stratigraphic excavations show that the site was built and functioned between 4680 and 4440 cal BCE, during the 2nd phase of the SMP culture (Fig. 3a). Within the newly exposed area, a new fireplace, termed the “northern hearth,” was found and dated to 4670–4543 cal BCE (Fig. 3b). This chronology was established through a new series of radiocarbon determinations. Samples were carefully taken from wooden posts, animal bones, charred seeds, and peat layers, and modelled using Bayesian statistical methods^79^. The northern hearth and its associated sediments were the focus of specific micromorphological analyses, which provided valuable insights into food preparation (including mollusc cooking practices) and a more accurate assessment of fish consumption, as well as into hearth cleaning and the management of domestic space^83^.

This new research season has also enabled systematic sampling for the study of macro- and micro-plant remains (SI Table S2), as well as terrestrial and freshwater faunal assemblages, to rigorously outline a subsistence economy framework for this site. In order to interpret the results within their archaeological context, the analysed stratigraphic units have been grouped into six macro-contexts: abandonment layers (i.e., areal layers covering the structures and the walking surface—SU 7, 9, 11), charcoal layers (i.e., charred materials scattered on the domestic floor during the later use of the combustion areas—SU 5, 20), northern hearth (i.e., a fire structure including the hearth plate and the related cleansing layers—SU 6, 42, 46, 48, 60, 66), reworked hearth residues (i.e., the reworked remains of smaller firing structures including burnt soil fragments and large amounts of charcoal—SU 21, 67, 72, 73), Barfield–Broglio hearth (SU 56), and pre-site peat layer (i.e., natural layer preceding the establishment of the settlement—SU 25).

## Results

### Plant macro-remains

The archaeobotanical analysis resulted in the extraction of 4337 plant remains, of which 2306 were identifiable to a taxonomic level. These remains exhibit a remarkable floristic variety, with 76 different taxa recognized and organized into six categories based on economic and environmental criteria. The complete table of results is available in the supplementary materials (SI Table S3).

**Fig. 4:**
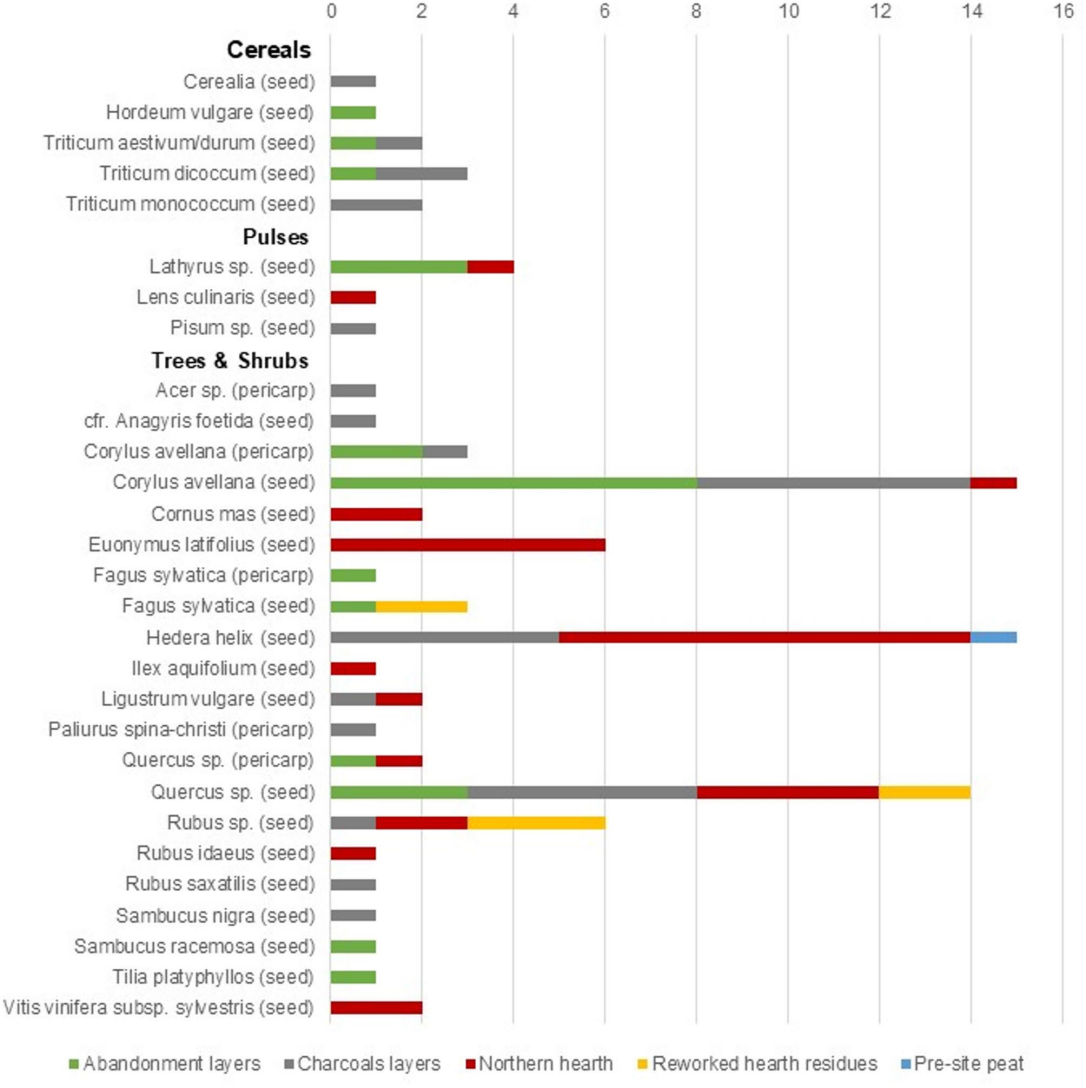
Chart showing the results of the carpological analysis for the macro-categories of economic interest: Cereals, Pulses, and Trees & Shrubs, grouped by provenance context.

Aquatic or wetland plants are by far the most represented category (1988 remains for 18 taxa), followed by grassland and undergrowth herbs (165 remains for 22 taxa), arboreal fruit remains (80 remains for 19 taxa), non-habitat-specific herbaceous plants (33 remains for 10 taxa), cereals (9 remains for 5 taxa) and pulses (6 remains for 3 taxa). Two taxa dominate the assemblage: the water chestnut (*Trapa natans*), with 868 charred remains (854 shell fragments and 14 seed fragments) and the Nymphaeaceae family with 822 uncharred seeds. *Cladium mariscus*,* Persicaria lapathifolia*,* Carex* sp., and *Chenopodium opulifolium* follow with significantly lower numbers; respectively 210, 94, 36 and 31 uncharred seeds. All other taxa found have quantities below 20 units. Categories of primary economic importance (cereals, pulses, and tree fruits) are represented by a total of 95 remains, most of which (67) come from edible plants (Fig. 4).

Among the cereals are caryopsis of emmer (*Triticum dicoccum*), einkorn (*T. monococcum*), naked wheat (*T. aestivum/durum*), and barley (*Hordeum vulgare*), while among the pulses are vetch (*Vicia* sp.), lentils (*Lens culinaris*), and peas (*Pisum* sp.) all documented by few units. It’s worth noting the total absence of chaff remains in the assemblage. Wild edible fruits include hazelnuts (*Corylus avellana*), acorns (*Quercus* sp.), beech nuts (*Fagus sylvestris*), berries (*Rubus* sp. *R. idaeus*, *R. saxatilis*) and other fleshy fruits (*Comarum palustre*,* Cornus mas*,* Sambucus nigra*,* Vitis vinifera* var. *sylvestris*).

A limited number of remains (15) similar to charred crumbs were classified as amorphous charred objects (AOV). They are considered food remains originating from fruit pulp or flour-based dough products^84–86^. As for the unidentified remains (2031), they are mostly charred nutshell fragments (866) and seed fragments (44) without diagnostic anatomical elements. A significant number of charred remains (1115) were too small and poorly preserved to be attributed to a taxon or a specific part of the fruit. Most of the remains in the entire assemblage (70.85%) are charred, while 29.15% are desiccated. This dehydration likely began with peat removal in the 1940s, since the Barfield-Broglio excavations (1969–1972) documented good preservation by waterlogging. Over the next 50 years, organic matter decomposed, but some elements were preserved. Therefore, the carpological record is richer than dry sites but poorer than wet sites.

### Plant micro-remains

To test whether a preservation bias could explain the absence of macroscopic charred chaff remains, we conducted a phytolith survey to check for the presence of pooid cereal phytoliths, which would indicate on-site crop processing. Cereals of the Pooideae grass subfamily such as barley, emmer, einkorn and other wheat species are extremely rich in phytoliths and have been widely studied, from modern reference material (see a review in Ball et al.^87^ as well as from neolithic archaeological sites^88–91^. The husks in the ears produce Elongate Dendritic phytoliths that are considered typical of the chaff of pooid cereals^87,92^, among further morphotypes diagnostic of the grass family. For this purpose, 17 sediment samples have been collected from 11 SU (SI Table S4) that corresponded to the main anthropogenic deposits and the natural substrate of lake marl as control. The results of the phytolith analysis (SI Table S4) shows that Elongate Dendritic phytoliths from chaff are absent in Molino Casarotto. The investigated units correspond to the main anthropogenic deposits, including the ashes from the northern hearth, and the natural substrate of lake marl as control (SU 10). At the site, phytoliths are few and corroded (0.003–0.08 g on 1 g of sediment; 0.8–32.5% of phytoliths in the AIF/Acid Insoluble Fraction, SI Figure S1) and they mostly originated from the leaves of monocotyledons such as common reeds, *Phragmites australis*, Cyperaceae and Poaceae (Blocky and Bulliform Flabellate, SI Figure S2). Sponge spicules are also present (SI Figure S2). The record of siliceous microfossils thus reflects the wild vegetation of the lakeshore humid environment.

### Terrestrial faunal remains

The terrestrial faunal remains will be discussed briefly, as they are few, extremely fragile and in a fragmentary state of preservation. Only 36 remains out of 779 have been anatomically and taxonomically determined—or at least assigned to their respective genus or a higher taxonomic level—(Table 1; SI Table S5) due to the reasons stated in the materials and method section. As for taphonomy, 16.8% of the remains showed burning traces, with evidence of exposure to fire at different temperatures^93^. Only two remains exhibited cut marks. Among the few remains that could be identified are elements of red deer (5 antler pieces, 2 teeth and a metatarsal), roe deer (a mandibular portion and 6 teeth), two Suidae elements identified as wild boar (a humerus and a radius) and cattle (2 teeth). Additionally, there are 4 teeth and a metapodial belonging to unidentified small carnivores (e.g., small canids or mustelids such as badgers), 5 teeth and 3 post-cranial elements of micromammals (mainly small rodents) and one Suidae humerus hardly identifiable as domestic pig or wild boar. The assemblage also includes 2 fragments of a turtle’s plastron and one from a bird. Among the unidentifiable fragments are diaphysis of small and large herbivores and pieces of tooth enamel from large herbivores. As for the rest of the fragments, it has not even been possible to hypothesize the size of the animal they belong to.

**Table 1:** Identified taxa from the site of Molino Casarotto. The table includes the results of archaeozoological analyses from previous excavations, as well as those presented in this study and related to the recent archaeological investigations. As for Jarman’s study, the numbers in brackets refer to specimens that he tentatively assigned to the taxon in question, while the question mark placed next to Rupicapra Rupicapra has been retained as originally indicated by Boyle. The exact quantity of molluscs identified in previous studies is not reported, as the publications do not provide the precise number of fragments recovered. Taxonomic categories are presented using Latin nomenclature, as in Jarman, with slight adjustments and/or groupings made for practical purposes.

TAXON	NISP (Jarman 1976)	NISP (Boyle 2014)	NISP (THIS STUDY)
*Bos taurus*	52 (2)	68	2
*Ovis/Capra*	24 (3)	119	
*Ovis aries*	3 (1)		
*Equus caballus*	1 (2)	6	
*Cervus elaphus*	2947 (166)	1036	8
*Capreolus capreolus*	275 (41)	288	7
*Rupicapra rupicapra* (?)		2	
*Sus scrofa*		467	2
*Sus* sp.	2285 (61)		1
*Vulpes vulpes*	1	21	
*Canis lupus*		3	
*Canis familiaris*		28	
Canidae	1		
*Ursus arctos*		17	
*Felis silvestris*		3	
*Meles meles*	59 (6)	25	
*Lutra lutra*		19	
*Martes martes*		3	
*Mustela* sp.		2	
*Mustela erminea*		4	
*Martes* sp.		1	
Mustelidae indet.		2	
Carnivora indet.	42 (6)		5
*Castor fiber*	4	1	
*Arvicola amphibius*		8	
*Rodentia* indet.	3		
Leporidae		20	
Lagomorpha indet.	2	6	
Micromammalia			8
*Anas platyrhynchos*		1	
*Falco* sp.		1	
Aves	11 (2)		1
Anura indet.	(2)		10
*Emys sp.*	14 (2)		2
*Chelonia* indet.	3 (1)		
*Esox lucius*			48
*Tinca tinca*			8
Cyprinidae sp.			14
Pisces	206 (1)	46	
*Helix* sp.			25
*Rumina decollata*			1
*Lymnea* sp.			2
*Planorbarius corneus*			1
*Unio* sp.			1840
*Viviparus* sp.			7
*Viviparus ater*			17
*Viviparus contectus*			81
Total NISP	5933 (296)	2197	2090
Medium/small-sized mammals			54
Large-sized mammals			11
Unidentifiable			677

The poor state of preservation of these remains is most likely attributable to taphonomic processes and the features of the recovery context rather than reflecting intensive carcass exploitation. The excavated area appears to be a domestic space that was kept relatively clean. The recovered faunal remains do not seem to result from food waste disposal or butchering activities—which were likely carried out and discarded elsewhere—but rather appear to represent residual materials.

### Freshwater faunal remains

**Fig. 5:**
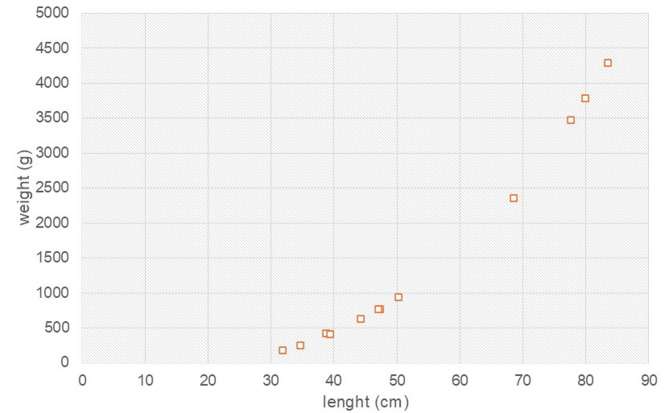
Size reconstruction of pike according to Frezza^172^ for cranial remains and Jelu et al.^173^ for vertebrae. Length is expressed in cm, while weight is expressed in g.

Compared to previous research, fewer fish bones were recovered in absolute terms, likely due to the much smaller excavated area in 2022 (≈ 60 m²) compared with approximately 450 m² in earlier investigations. However, the 2022 assemblage is proportionally richer thanks to targeted recovery and improved sieving techniques (Table 1). Pike is the most frequent taxon, with cyprinid remains also present. Among cyprinids, tench skull bones were identifiable at taxonomic level, but vertebrae could not be further identified. Both pike and tench inhabit shallow, calm waters. Generally, pikes measure around 20 cm in their first year and can grow up to 1 m in length and over 10 kg in weight in adulthood; tench generally measure between 30 and 50 cm and weigh up to 6 kg. Fish bones were distributed among the main archaeological features, with less significant layers included only in the overall record (Table 1). Identified elements mainly include those useful for taxonomic identification, such as cyprinid pharyngeal bones (SI Table S5). 19 fish bones exhibited burn marks, with no species or anatomical elements showing a higher frequency. This data is consistent with the presence of burning structures at the site. Size reconstruction was performed for pike remains (SI Table S6-S7, Fig. 5), but not for tench bones due to their fragmented state. Pike sizes ranged from 31.8 to 83.4 cm, with weight from approximately 182.29 gr to 4.3 kg. Notably, only one individual exhibited significant size and weight, while most others measured between 30 and 40 cm and weighted between 180 and 400 gr. Lastly, seasonality data were obtained from seven pike vertebrae, only one of which was recovered from SU 21, with the remaining refer to SU 20. These data suggest that pikes were caught during the warmer seasons (i.e., spring–summer). Due to the limited number of specimens, this evidence cannot be considered statistically or interpretively significant on its own. The poor preservation of other fish vertebrae, including those of cyprinids, prevents us from drawing further conclusions about seasonality patterns related to fishing. A substantial number of mollusc shells were recovered (Table 1) with *Unio* sp. shells being the most frequent.

A recent study suggests that *Unio elongatulus* is currently prevalent in the Adriatic drainages of the Italian peninsula, whereas *Unio mancus* is more common in the Tyrrhenian and Ionian freshwater courses, making them allopatric *taxa* separated by the Appennines^94^. This could imply that Molino Casarotto shells may belong to *Unio elongatulus*; however, these species distribution during the Neolithic is uncertain and limited information from archaeological sites could be misleading. Although NISP suggests a high frequency of mollusc remains, the MNI points to a lesser significance at the site. Only 11 *Unio* sp. individuals were identified following Girod^95^, confirmed by the presence of 22 umbones, whose laterality could not be determined. For other species, the MNI calculation was facilitated by the intact shells. Unidentified snail remains were also counted, though it was impossible to distinguish whether they were freshwater or terrestrial species. Freshwater mollusc remains dominate the assemblage: Unionidae are commonly found on swamp and river bottoms; Lymnaeidae and *Planorbarius corneus* typically inhabit shallow, calm waters; lastly, various species of the Viviparidae family are frequent in ponds and swamps. Some land species were also recovered, but their presence may be intrusive. The presence of terrestrial gastropods in the assemblage, although minimal, is likely the result of post-depositional intrusion. Land snails are commonly regarded as intrusive elements in archaeological contexts, particularly in sheltered environments, where they may accumulate due to favourable microhabitat conditions such as moisture and shelter provided by stone structures or cavities. Their limited presence in this context suggests passive introduction rather than deliberate human-related deposition and is unlikely to reflect significant paleoenvironmental or behavioural implications^95^. Freshwater mollusc species were further divided into bivalves and gastropods, with unidentified snail fragments also considered to better gauge the quantity of molluscs consumed. These two classes were analysed based on their NISP and weight: the 1840 bivalves weigh 235,78 gr, while the 664 gastropods weigh 45,57 gr. This analysis indicates that, despite their high number, *Unio* sp. shell fragments exhibit a significant degree of fragmentation. It can be concluded that gastropod shells are fewer in number and species but slightly better preserved, while bivalves are more fragmented.

### Statistical analysis

The analyses were conducted on published data from 53 different assemblages from a total of 49 sites, distributed across seven geographical areas and three chronological phases. Archaeobotanical data were available for 28 contexts, faunal data for 37 contexts, and in 12 cases both proxies were available from the same context.

**Fig. 6:**
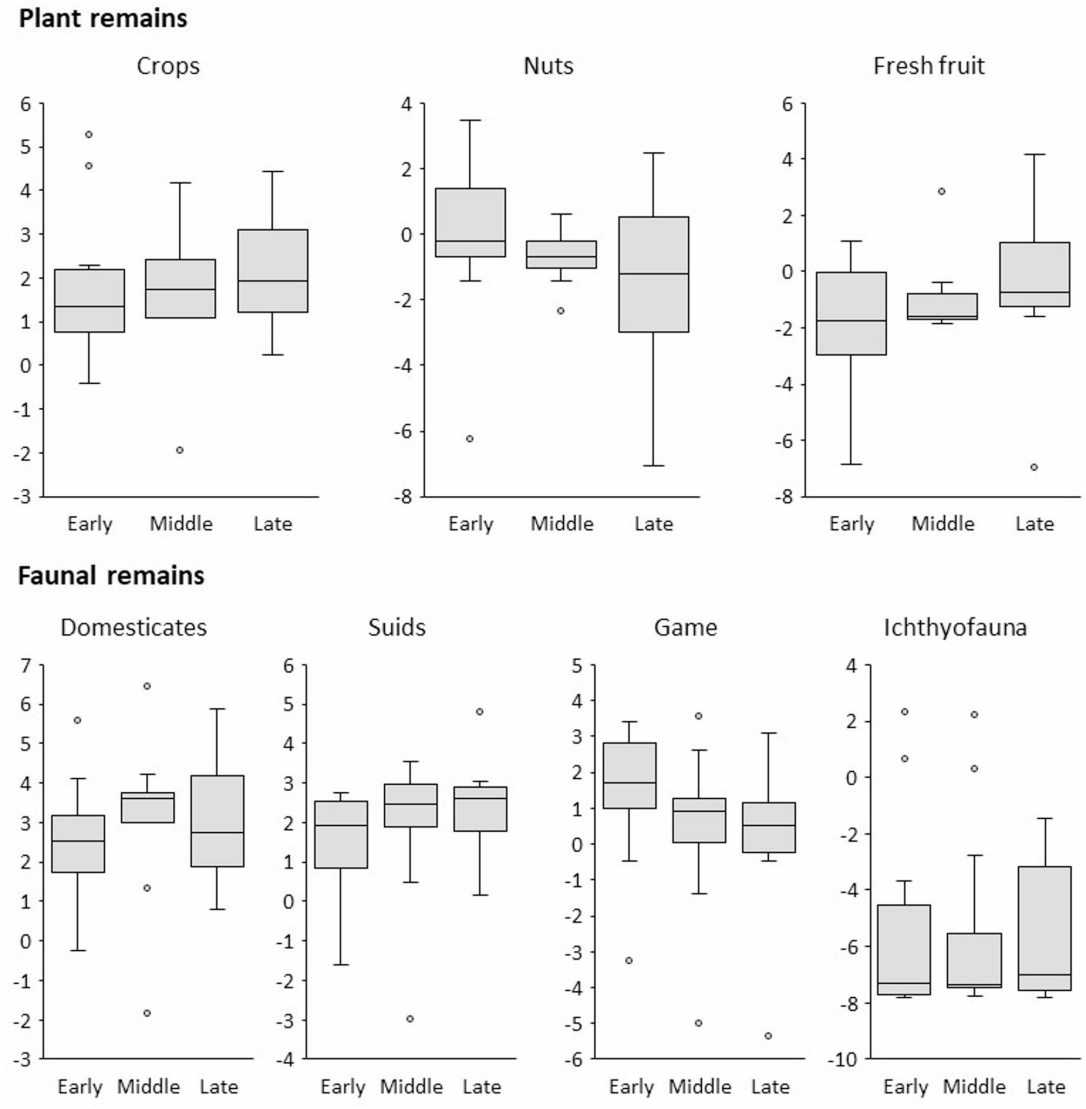
Boxplots of CLR-transformed abundance data of different plant and animal remains, grouping the archaeological sites by chronology (Neolithic phase).

With regard to the most significant chronological trends, the univariate analysis highlights distinct patterns in plant and faunal assemblages across the early, middle, and late Neolithic (Fig. 6). Cultivated crops show a progressive increase over time, reaching their highest values in the late Neolithic. A similar trend is observed for fresh fruits, whereas nuts are more abundant in the early Neolithic and decline markedly in later phases. Animal resources display more variable patterns: domestic species increase progressively, while wild game declines. Suids, however, deviate from this trend, peaking in the middle Neolithic and then remaining stable, whereas fish and mollusc remains, although less abundant, are present throughout the sequence with only minor fluctuations. The results of the stepwise correlation analysis, based on the Pearson coefficient (r) and the associated p-value for each pair of variables, reveal a series of moderate to strong correlations that are statistically significant (Table 2). For plant remains, negative correlations are observed between crops and nuts and between nuts and fresh fruit. Among animal remains, domesticates are positively correlated with suids and negatively correlated with game. Ichthyofauna shows a negative correlation with all other animal variables, with potential sampling biases already noted. Overall, these results confirm the univariate analysis: increasing farming and herding activities are associated with a decline in gathering, hunting, and fishing. The negative correlations between nuts and fresh fruit, as well as game and ichthyofauna, likely reflect differences in environmental contexts or resource availability.

**Table 2:** Stepwise correlation analysis of CLR-transformed abundance data of different plant and animal remains from all the selected archaeological sites; for each pair of variables, the pearson correlation coefficient (first row) and the p-value (second row) are indicated.

Plant remains				
	Crops	Nuts	Fresh fruit	
Crops		− 0.3940	− 0.2650	
		0.0380	0.1729	
Nuts	− 0.3940		− 0.7818	
	0.0380		0.0000	
Fresh fruit	− 0.2650	− 0.7818		
	0.1729	0.0000		

Animal remains				
	Domesti-cates	Suids	Game	Ichthyofauna
Domesticates		0.5801	− 0.3601	− 0.5645
		0.0002	0.0310	0.0003
Suids	0.5801		− 0.0013	− 0.7813
	0.0002		0.9940	0.0000
Game	− 0.3601	− 0.0013		− 0.4852
	0.0310	0.9940		0.0027
Ichthyofauna	− 0.5645	− 0.7813	− 0.4852	
	0.0003	0.0000	0.0027	

**Fig. 7:**
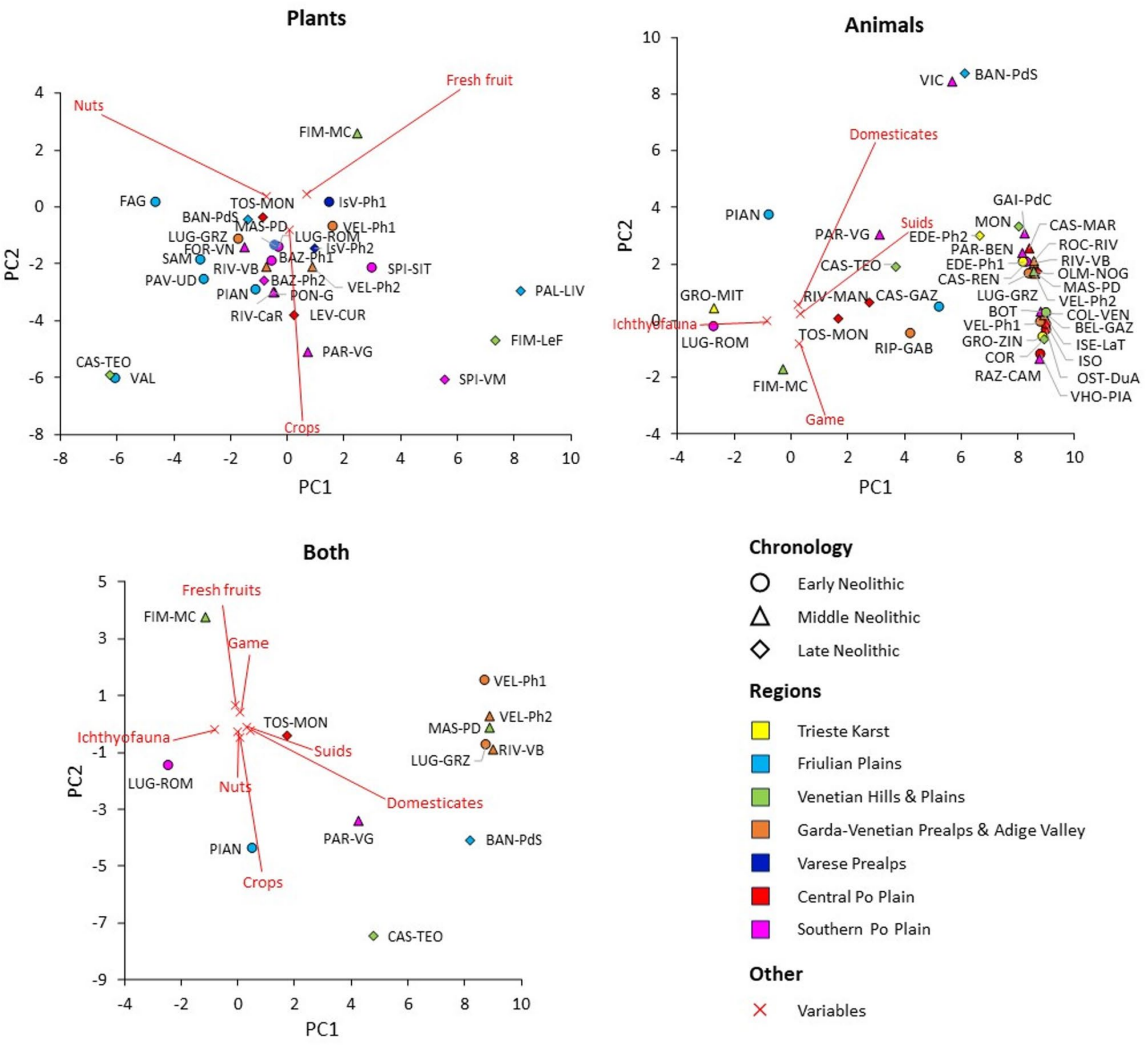
PCA biplots of CLR-transformed abundance data of plant remains (top left), animal remains (top right), and plant and animal remains together from the sites containing both (bottom). PC1 and PC2 account for 100%, 96%, and 90% of total variance, respectively.

**Fig. 8:**
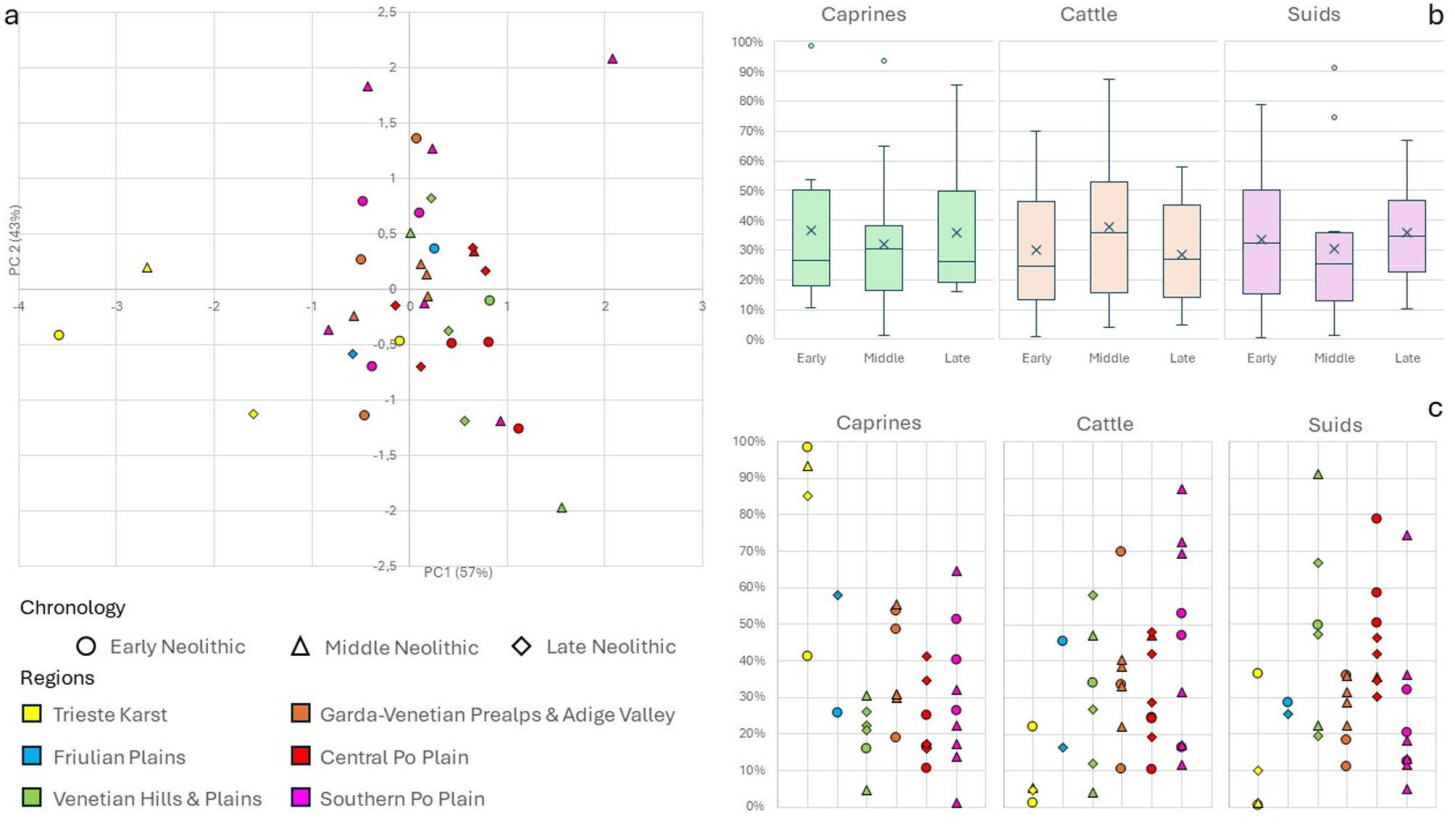
Integrated visualization of livestock exploitation patterns across Neolithic northern Italy. **a** PCA of score plots based on log-ratios between caprine, bovine, and suid remains. **b** Boxplots illustrating chronological trends in the relative abundance of each taxon. **c** Scatter plots showing regional variation in relative abundances, with colors indicating regions and shapes representing chronological phases.

The PCA biplots are shown in Fig. 7. Overall, clear chronology- or geography-based clustering is absent. In the PCA of plant remains, an apparent left-to-right pattern is observed, roughly corresponding to early to late Neolithic sites, with nuts more prevalent on the left and crops and fresh fruits more prevalent on the right. In the PCA of faunal remains, two clusters appear at the right end of the plot, both representing mixed economies: in the lower cluster (mainly early Neolithic sites) wild game is more prominent, while in the upper cluster (mainly middle Neolithic sites) domesticates are more prevalent. In the PCA combining plant and animal remains, a cluster of sites from the Garda-Veneto Prealps and Adige Valley—including one site from the neighboring Venetian area—forms a relatively homogeneous and stable group over time in this region. In this plot, sites can be broadly divided into three groups: at the bottom, sites with predominantly agro-pastoral economies; at the top, Molino Casarotto (Fig. 7-FIM-MC), with an economy largely based on hunting, fishing, and gathering; and in the central band, sites with relatively mixed economies integrating agriculture, gathering, herding, hunting, and fishing. Across all PCA analyses, a large, scattered cluster includes most sites of varying chronology and regions, highlighting the variability of Neolithic economic systems. For the four sites with data from two distinct chronological phases (Isolino Virginia, Fig. 7-IsV, Bazzarola, Fig. 7-BAZ, La Vela, Fig. 7-LaV, Grotta dell’Edera, Fig. 7-EDE), the later phases show a higher incidence of agro-pastoral indicators compared with the earlier phases. Analyses focusing on livestock-related taxa (caprines, cattle, and suids) revealed widespread mixed herding systems, with regional patterns evident in the multivariate analysis (Fig. 8a). These were further explored through univariate analyses of chronological trends (Fig. 8b) and regional variation (Fig. 8c), which highlighted central tendencies and variability across Neolithic phases and diverse regional livestock management strategies that will be discussed in detail below.

## Discussion

### Food choices at Molino Casarotto

**Fig. 9:**
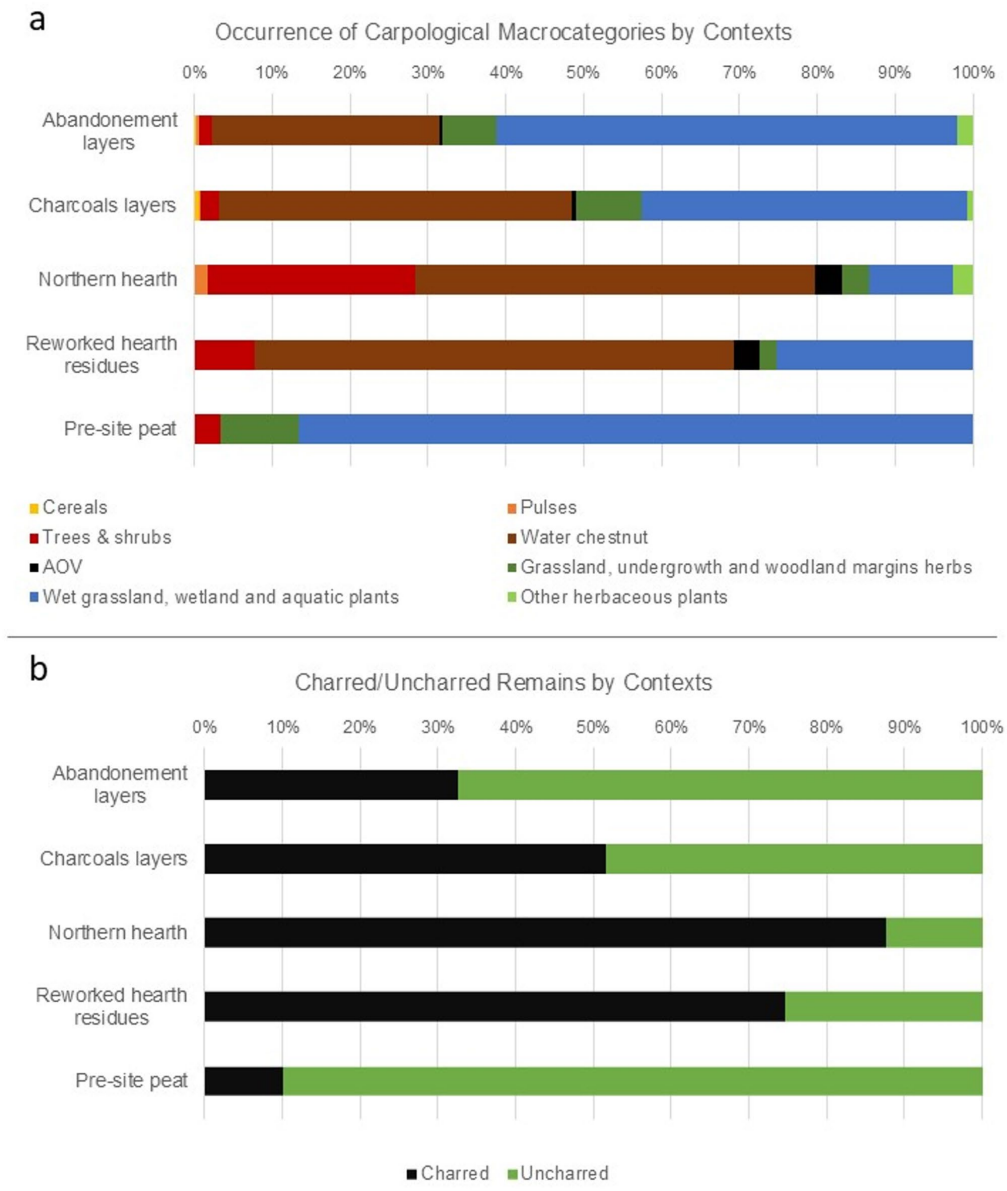
**a** Occurrence of plant macro-remain categories in the contexts analysed; **b** relative proportions of charred and uncharred plant macro-remains in the contexts analysed.

The reconstruction of food supply choices must begin with a taphonomic consideration. The comparison between the occurrence of carpological macro-categories across the analysed contexts reflects the ratio between charred and uncharred remains. (Fig. 9). The graphs clearly show that the sum of seeds/fruits of cereals, pulses, arboreal plants, water chestnut, and AOV almost perfectly matches the proportion of charred remains, whereas that of grassland and undergrowth herbs, other non-edible aquatic/wetland plants, and herbaceous plants of non-specific-habitat matches the uncharred remains. The former thus have a paleo-economic significance mainly related to dietary use, while the latter provide additional information, primarily of paleoenvironmental nature. This interpretation aligns with the results of phytolith analysis indicating a predominance of wetland and aquatic species. The ubiquitous presence of Nymphaeaceae small seeds—particularly abundant in the pre-site peat and in the abandonment layers—suggests the persistence of shallow, stagnant-water or marshy conditions before and after human occupation of the site.

**Fig. 10:**
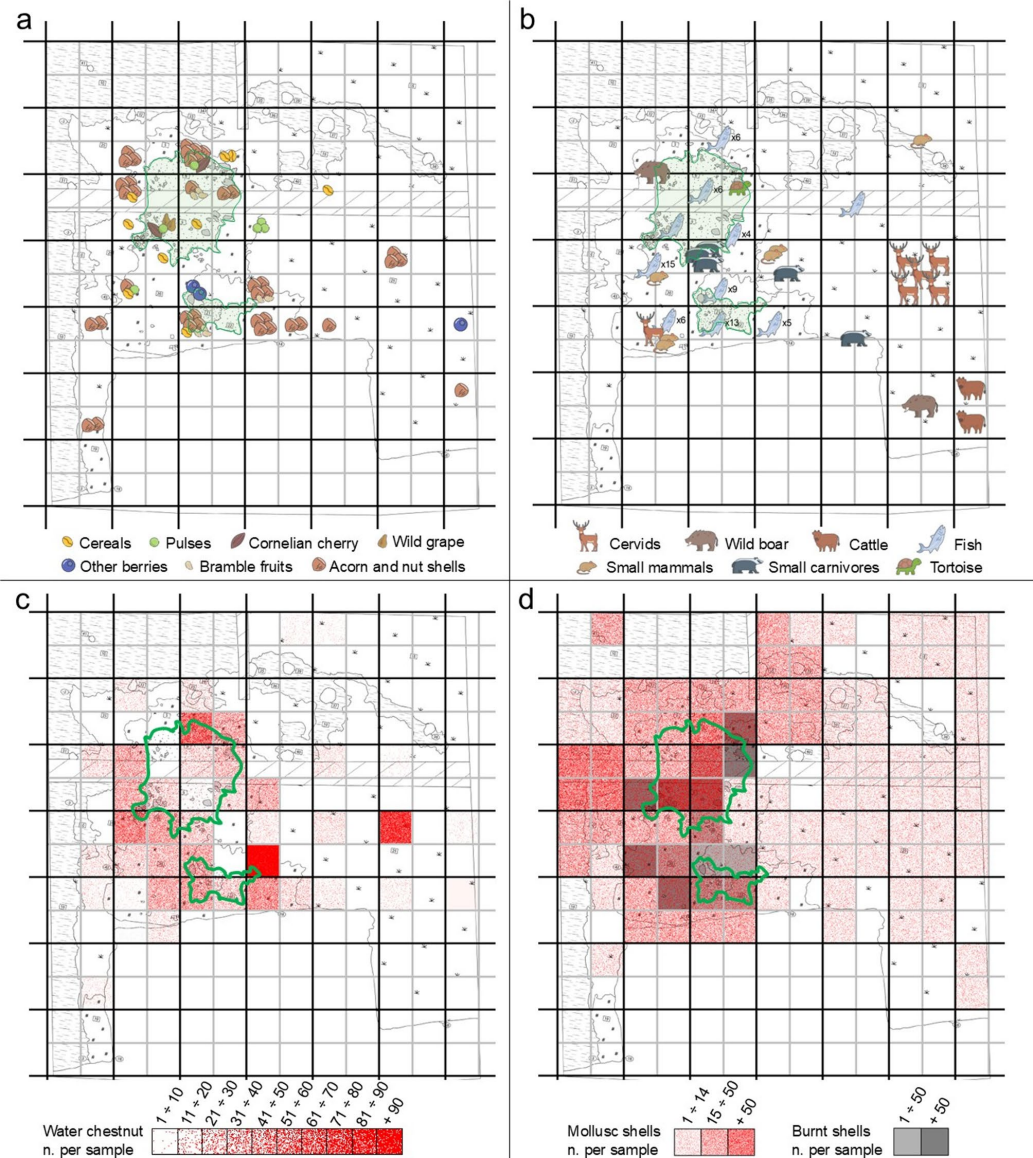
Spatial distribution of plant and bone remains in the samples collected within the 2022 excavation area (the two fire structures identified are highlighted in green): **a** distribution of cereals, pulses, fruits, and nuts (the drawing shows the exact number of remains identified); **b** distribution of mammal, reptile, and fish bone remains. (the drawings indicate the exact number of remains, for fish remains the exact number is shown where the value was too high for a graphical representation); **c** distribution pattern of charred water chestnut (*Trapa natans*) shell fragments; **d** distribution pattern of mollusc shell fragments and burned shells.

The spatial distribution of charred remains shows that cereals, pulses, berries, and arboreal fruits are concentrated near the two fire structures (Fig. 10a) suggesting the simultaneous use of gathered and cultivated plant foods and their accidental combustion through discard near the hearths, thus representing meal residues. Bones of terrestrial fauna and fish (Fig. 10b) show a similar distribution, being highly fragmented and fire-exposed. The scarcity of complete skeletal elements points to the cleaning of domestic spaces, with larger refuse discarded farther away. Small fish bones are ubiquitous around the hearths, while medium- and large-sized mammal bones (suids, cervids, cattle) occur mainly a few meters east of them. Micromammals, small carnivores, and tortoise remains follow the same pattern as fish bones, supporting the interpretation that rake-out deposits around the hearths retained finer material, while bulkier waste was removed. Within this framework, the considerable number of fish bones suggests the primary economic importance of fishing at the site, in line with previous interpretations by Barfield and Broglio. The presence of tench (30–50 cm) and pike (30–40 cm) indicates the use of traps and nets in the nearby lake, while larger pike (70–80 cm) point to additional techniques such as harpoons and hooks, similar to those proposed for Lugo di Romagna^96^.

Mollusc shells are more widely scattered (Fig. 10d), occurring throughout almost the entire excavation area. This distribution may be due to fragmentation and small size, with burned shells found only near cooking structures and absent in contexts without them. This pattern suggests shell disposal around hearths and a possibly post-depositional origin of burning traces^97^. The distribution of water chestnut remains deserves particular attention (Fig. 10c). Although most are concentrated near the hearths, they are also widespread across the site and display the highest ubiquity value (SI Table S9). Nearly all water chestnut remains (98%) are charred shell fragments, even those far from hearths. Since the edible nut must be extracted from the shell and can be consumed raw, boiled, or roasted^98^, the evidence points to deliberate roasting and shelling. Combustion, therefore, was likely part of a specific processing sequence rather than accidental or post-depositional. Water chestnut is by far the most abundant food resource: a conservative estimate indicates at least 213 collected fruits—a remarkable quantity given the limited excavation area—suggesting it served as a staple food. Nutritionally comparable to cereals in starch and protein content^98,99^, this plant likely played a central role in the site’s subsistence. Abrasive stone fragments with flattened surface found near the northern hearth could represent pieces of a grinding tool used to process roasted nuts into flour, a practice documented historically and ethnographically^100^. Some AOVs in the macro-remain assemblage might represent the final product; however, no residue, functional, or petrological analyses have yet been conducted, and this interpretation remains hypothetical.

The predominance of foraging-based strategies at Molino Casarotto is evident. Domestic mammals identified at species level account for only 3.76% of the assemblage (5,680 remains). The “suids” category, representing 35.04%, complicates interpretation, as distinguishing between domestic pigs and wild boar is notoriously challenging in Neolithic contexts^101–103^. However, previous biometric and demographic reanalysis of the Barfield-Broglio faunal assemblage^104^ does not indicate a substantial presence of introduced domestic pigs at the site, suggesting that most suid remains are most likely wild individuals. This, in addition to the high proportion of certainly wild species in the assemblage (61.20%), supports the interpretation of a subsistence economy primarily based on hunting, fishing, mollusc exploitation, and wild fruit gathering—categories that together account for almost the entire archaeobotanical and archaeozoological assemblage. The use of water chestnut as a staple food further reflects a foraging-oriented adaptation, probably linked to the abundant availability of this resource near the site, as observed in other wetland neolithic sites across the Balkans and Central Europe^74,98,100^. This strategy aligns with the broader middle Neolithic pattern of increasing environmental diversification and flexible resource use. The deliberate roasting, shelling, and possibly grinding of the fruits indicate a degree of processing conceptually comparable to that applied to hulled cereals, suggesting structured food preparation practices rather than immediate consumption. Finally, the site’s material culture—including greenstone axes from the Oltrepò pavese (southwestern Lombardy)^46^, flint from the Lessini Mountains^79^, and SMP pottery—demonstrates integration into regional exchange networks of raw materials, suggesting that such interactions could also have involved plant or animal food resources, though further analyses are required to test this hypothesis.

### Neolithic subsistence economies in Northern Italy

The environmental variability of Holocene northern Italy—encompassing plains, forests, coastal zones, hilly areas, mountain, valleys, and wetland or lacustrine environments—had long provided favourable conditions for human settlement and diversified resource exploitation. During the late Mesolithic, this is reflected in the systematic occupation of lowland areas, particularly near watercourses, springs, and wetlands^105–111^. Subsistence economies in these areas were based on hunting and hazelnut gathering, as well as fishing and mollusc collection^105,111^. This is indicated by archaeobotanical and archaeozoological assemblages, along with the frequent recovery of antler harpoons from sites across the Alpine region and northeastern Italy^112^.

The same ecological richness offered early Neolithic farmers a wide range of resource opportunities and may partly explain the persistence of flexible and locally adapted subsistence strategies throughout the Neolithic.

**Fig. 11:**
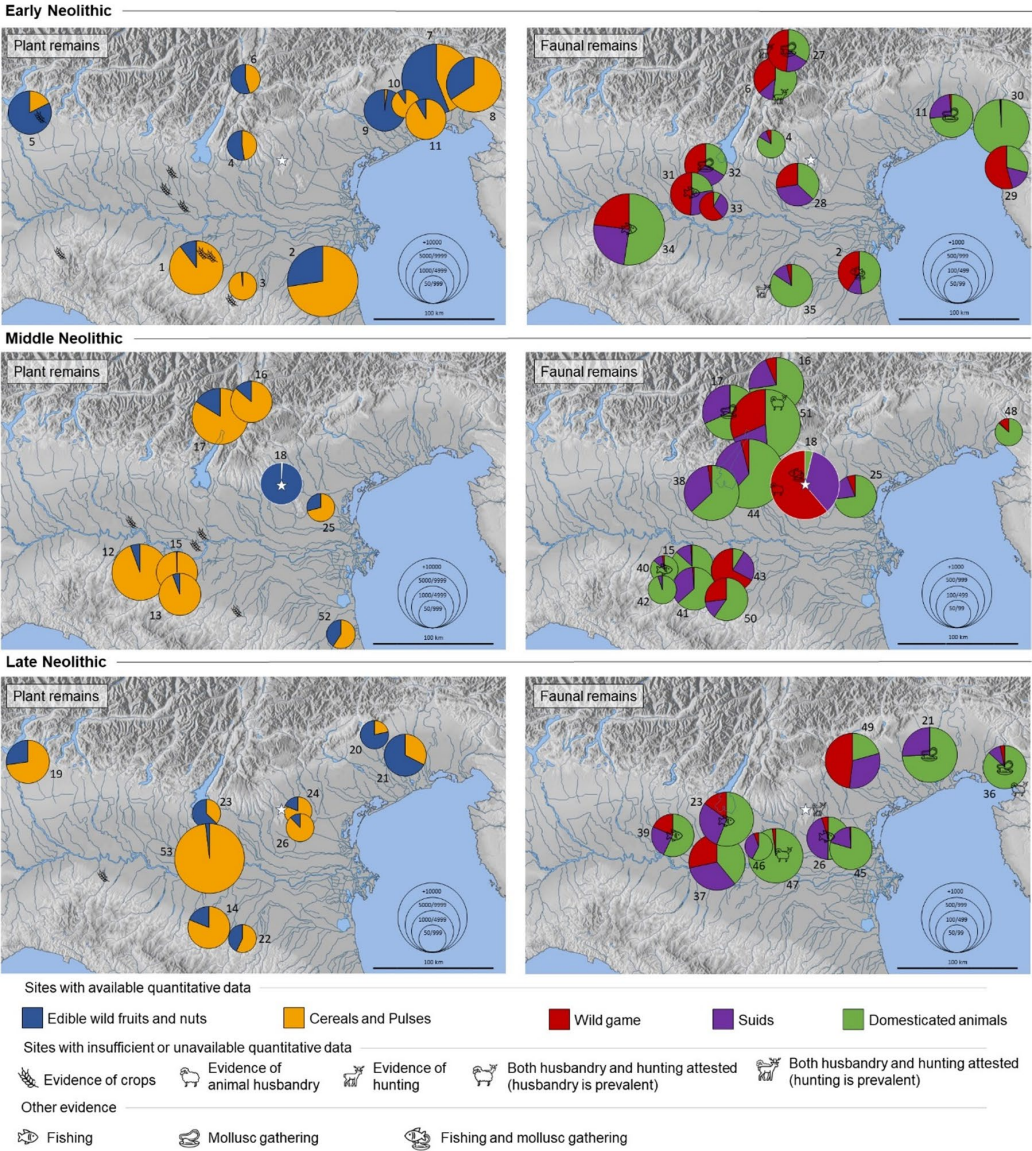
Agriculture/gathering and livestock/hunting in early, middle and late Neolithic northern Italy. Sites with quantitative data are represented by pie charts, their size corresponding to the number of analysed remains. Sites with insufficient or no quantitative data are depicted with a specific icon. Additionally, evidence of fishing and mollusc gathering are indicated with appropriate symbols. Numbers indicate the respective sites (see Tables 3, 4), while the white star and charts outlined in white indicate the site of Molino Casarotto. Map modified from Maps-for-Free/OpenStreetMap (© OpenStreetMap contributors, licensed under ODbL, https://www.openstreetmap.org/copyright).

From the early Neolithic onward, subsistence economies in northern Italy developed along multiple trajectories, likely reflecting both environmental diversity and cultural variability. Our review, based on the available data from 49 sites distributed across seven geographic regions and three chronological phases, together with the related univariate and multivariate analyses, provides a general overview of Neolithic subsistence patterns. In general terms, cultivated crops, fresh fruits, and domesticated animals increased progressively from the early to the late Neolithic, while wild resources such as nuts and game tended to decline (Fig. 6). Aquatic resources, including fish and molluscs, although minoritarian, remained consistently represented throughout the sequence. In contrast, PCA, while showing possible temporal trends and regional clustering, highlights the full complexity of subsistence economies, revealing a broad spectrum of mixed strategies rather than a single, homogeneous model (Fig. 7). To further explore these patterns, Fig. 11 presents six maps of northern Italy, each showing all available sites for a given chronological phase. For each site, pie charts illustrate the relative proportions of plant food sources (edible wild nuts and fruits vs. cultivated crops—Table 3) and meat sources (wild game, suids, domesticates—Table 4). This visualization allows a detailed comparison of site-specific economic strategies.

**Table 3:** Sites with available quantitative data on archaeobotanical analyses divided by chronology (EN = Early Neolithic, MN = Middle Neolithic, LN = Late Neolithic). Cultivated crops include cereals (caryopses and chaff remains) and pulses, while gathered fruits encompass all remains of edible wild fruits, including nutshell fragments.

*n*.	Site	Region	Phase	*n*. remains	Cultivated crops (%)	Gathered fruits (%)
1	Bazzarola—phase 1^115^	South. Po plain	EN	8383	89.54	10.46
2	Lugo di Romagna^116^	South. Po plain	EN	11,088	72.62	27.38
3	Spilamberto—via Macchioni^117^	South. Po plain	EN	153	98.69	1.31
4	Lugo di Grezzana^120^	Venet. plain&hills	EN	807	47.34	52.66
5	Isolino Virginia—phase 1^122,123^	Varese Prealps	EN	3233	17.65	82.35
6	La Vela—phase 1^121^	Adige valley	EN	126	45.24	54.76
7	Sammardenchia^36,38,118^	Friulian plains	EN	10,166	44.04	55.96
8	Pavia di Udine^174,175^	Friulian plains	EN	5852	65.74	34.26
9	Fagnigola^36,38^	Friulian plains	EN	4089	2.03	97.97
10	Valer^36,38^	Friulian plains.	EN	52	90.38	9.62
11	Piancada^176^	Friulian plains	EN	1478	91.47	8.53
12	Ponte Ghiara^115^	South. Po plain	MN	6597	94.25	5.75
13	Rivaltella Cà Romensini^115^	South. Po plain	MN	1000	94.30	5.70
15	Parma—via Guidorossi^177^	South. Po plain	MN	3372	99.61	0.39
16	La Vela—phase 2^121,178^	Adige valley	MN	4656	86.38	13.62
17	Riva del Garda—via Brioni^178^	Adige valley.	MN	6050	83.82	16.18
18	Fimon—Molino Casarotto	Venet. plain&hills	MN	1876	0.80	99.20
25	Maserà di Padova^179^	Venet. plain&hills	MN	312	71.15	28.85
52	Forlì—via Navicella^117^	South. Po plain	MN	397	59.19	40.81
14	Bazzarola—phase 2^115^	South. Po plain	LN	1809	81.04	18.96
19	Isolino Virginia—phase 2^122,123^	Varese Prealps	LN	2220	72.75	27.25
20	Palù di Livenza^124^	Friulian plains	LN	306	21.57	78.43
21	Bannia—Palazzine di Sopra^125^	Friulian plains	LN	2197	32.54	67.46
22	Spilamberto—Site VIII^180^	South. Po plain	LN	194	70.62	29.38
23	Tosina di Monzambano^126^	Central Po plain	LN	137	37.23	62.77
24	Fimon-Le Fratte^181^	Venet. plain&hills	LN	601	81.03	18.97
26	Castelnuovo di Teolo^144^	Venet. plain&hills	LN	302	87.75	12.25
53	Levata di Curtatone^115^	Central Po plain	LN	12,275	98.12	1.82

**Table 4:** Sites with available quantitative data on archeozoological analyses divided by chronology (EN = Early Neolithic, MN = Middle Neolithic, LN = Late Neolithic). The data on domestic (caprines and cattle), suids (pigs/wild boars) and wild fauna are reported as published by the respective authors (only the confirmed identifications were considered). The last two columns indicate the sites that have provided evidence of fishing (fi) and mollusk gathering (mo).

*n*.	Site	Region	Phase	*n*. remains	Domestic (%)	Suids (%)	Wild (%)	fi	mo
27	Riparo Gaban^58^	Adige valley	EN	449	33.60	18.00	48.40		X
6	La Vela—phase 1^102^	Adige valley.	EN	104	51.92	11.54	36.54		
4	Lugo di Grezzana^136^	Adige valley	EN	67	83.58	10.45	5.97		
28	Cologna Veneta^182^	Venet. plain&hills	EN	357	36.97	35.58	27.45		
11	Piancada^132,133^	Friulian plain	EN	307	73.29	25.08	1.63		X
29	Grotta degli Zingar^183^	Trieste Karst	EN	228	28.95	16.67	54.39		
30	Grotta dell’Edera—phase 1^184,185^	Trieste Karst	EN	900	99.12	0.44	0.44		
31	Ostiano Dugali Alti^65^	Central Po plain	EN	236	22.46	28.39	49.15	X	
32	Isorella^67,69^	Central Po plain	EN	189	33.33	29.63	37.04		X
33	Vhò di Piadena^58,103^	Central Po plain	EN	97	8.25	30.93	60.82		
34	Casa Gazza^70^	Southern Po plain	EN	1409	52.45	24.63	22.92	X	
35	Casalecchio di Reno^135^	Southern Po plain	EN	195	84.62	11.79	3.59		
2	Lugo di Romagna^96,141,186–188^	Southern Po plain a	EN	243	49.61	11.11	41.98	X	X
16	La Vela—phase 2^102,189^	Adige Valley	MN	749	72.63	20.83	6.54		
17	Riva del Garda—via Brione^58,145,189^	Adige Valley.	MN	702	68.38	31.48	0.14		X
18	Fimon—Molino Casarotto^62,68,78^	Venet. plain&hills	MN	7862	3.76	35.04	61.20	X	X
38	Casatico di Marcaria^190^	Central Po plain	MN	850	63.06	34.71	2.24		
40	Parma—Benefizio^143^	Southern Po plain	MN	80	86.25	11.25	2.50	X	
41	Gaione, Parco del Cinghio^143^	Southern Po plain	MN	172	63.37	35.47	1.16		
42	Vicofertile^143^	Southern Po plain	MN	99	94.95	5.05	0.00		
15	Parma—via Guidoross^143^	Southern Po plain	MN	394	86.80	12.18	1.02		
43	Razza di Campegine^137^	Southern Po plain	MN	159	8.81	23.90	67.30		
44	Rocca di Rivol^101^	Adige valley	MN	1155	61.56	34.46	3.98		
25	Maserà di Padova^191^	Venet. plain&hills	MN	407	73.00	20.90	6.10		
48	Grotta del Mitreo—Trincea 5^58,134^	Trieste Karst	MN	89	86.52	1.12	12.36		
50	Botteghino^138^	Southern Po plain	MN	218	59.63	13.30	27.06		
51	Isera—La Torretta^139^	Adige Valley	MN	3927	48.76	19.66	31.58		
39	Rivarolo Mantovano^142^	Central Po plain	LN	168	57.14	24.40	18.45	X	
36	Grotta dell’Edera—phase 2^185^	Trieste Karst	LN	493	86.82	9.74	3.44		X
37	Belforte di Gazzuolo^64^	Central Po plain	LN	766	39.03	32.64	28.33		
26	Castelnuovo di Teolo^144^	Venet. plain&hills	LN	319	50.20	44.80	5.00	X	
45	Monselice^191^	Venet. plain&hills	LN	231	79.60	19.50	0.90		
46	Olmo di Nogara^192^	Central Po plain	LN	71	57.70	36.60	5.70		
49	Cornuda^140^	Venet. plain&hills	LN	817	20.70	31.10	48.20		
21	Bannia—Palazzine di Sopra^193,194^	Friulian plain	LN	596	74.30	25.70	0.00		X
47	Gazzo Veronese—Scolo Gelmina^195^	Central Po plain	LN	821	98.00	n/d	2.00		
23	Tosina di Monzambano^146,147^	Central Po plain	LN	662	55.89	29.00	15.11	X	

The introduction and spread of early agriculture in northern Italy likely followed multiple routes, including both maritime and terrestrial paths along the Tyrrhenian and Adriatic coast^113^, as well as from the Balkan area, as suggested by the presence of *Triticum timophevii*^114^, a species widely distributed in the latter region. The main introduced crops were barley, emmer, and einkorn, while free-threshing wheats are less frequent and pulses are generally poorly represented. Lentil, pea, *Lathyrus sativus/cicero*, *Vicia ervilia*, and *V. sativa* are documented, with *V. sativa* being more abundant in some sites^57^. The Adriatic diffusion route of agriculture is visible in the early appearance of crops in the Romagna and southern Po Valley, at Lugo di Romagna, Bazzarola, and Spilamberto^115–117^. While in these areas agriculture was predominant from its introduction, in the eastern Friulian plain, alongside sites with dominant or prevalent agriculture, the gathering of wild fruits—mainly hazelnuts—is well attested at Sammardenchia (56%) and Fagnigola (98%)^36,118,119^. A westward diffusion gradient in the spread of agriculture may be indicated by the progressive decrease in crop percentages, shifting from 87% in the Southern Po Plain and 60% in the Friulian plains, to about 45% at Lugo di Grezzana and La Vela^120,121^, and reaching around 20% at Isolino Virginia^122,123^, further west. During the middle Neolithic, when the SMP culture prevailed in most of the study area, agriculture became predominant across most regions, with crops exceeding 94% in the Southern Po Plain and around 85% in the Garda Prealps and Adige Valley. The importance of free-threshing wheats also seems to increase^57^. Deviating from this pattern are the assemblages from Maserà di Padova (crops = 71%) and Forlì-Via Navicella (60%), where crops are less abundant but still prevalent. Molino Casarotto—as previously discussed—represents the most striking exception, with crop frequencies below 1%. In the late Neolithic—a period characterized by the fragmentation of earlier cultural uniformity—agriculture is widespread, but an apparent increase in the incidence of wild fruits is observed, particularly where abundant remains are preserved under waterlogged conditions. This is evident at Tosina di Monzambano (63%), and Palù di Livenza (78%), but also at the dry site of Bannia-Palazzine di Sopra (67%)^124–126^ While this pattern is likely influenced by preservation bias, it may also reflect an actual greater exploitation of fresh fruits concurrent with the decline of nut gathering, as already noted in the univariate analysis. In particular, wild grape (*Vitis vinifera* ssp. *sylvestris*) becomes frequent, even if it does not constitute a significant dietary component, while hazelnut shell fragments are less common than in earlier phases^57^.

The introduction and spread of animal husbandry in northern Italy followed the same diffusion routes as agriculture, with domesticates such as sheep, goats, and cattle introduced through multiple pathways^127^, including the Tyrrhenian, Adriatic, and Balkan areas. The situation is more complex for pigs. Given that distinguishing between wild boar and domestic pig is often problematic and debated^101^, we opted to use the inclusive category of “suids”, encompassing both taxa. This choice allows for a clearer understanding of suid management practices, regardless of their precise domestic status, since their domestication pathway appears to differ from that of caprines and cattle. The difficulty in distinguishing wild from domestic suids in Neolithic assemblages is not only methodological but also historical and economic. Although it is undisputed that west Asian domestic pigs were introduced into Europe during the Neolithization process, genetic, biometric, and demographic data^104,128,129^ support the hypothesis of a local domestication process of the Italian wild boar during the Neolithic^129–131^. This interpretation, though still under discussion, suggests the emergence of local pig populations retaining morphological traits of the native wild boar, with limited genetic input from introduced stock. Tecce (2020) provides several arguments in favor of this scenario, including the absence of abrupt morphometric changes between Mesolithic and Neolithic suids, the persistence of the typically small Italian wild boar tooth size relative to postcranial bones, and the biometric similarity of Neolithic pigs to local wild boars rather than west Asian specimens^104^. Pending further evidence, this remains a plausible working hypothesis. Univariate analysis shows that suids follow a chronological trend more similar to that of domesticates and inversely related to wild game (Fig. 6). This suggests that—whether locally domesticated or imported—their presence broadly reflects herding practices, although exceptions exist. Examining the distribution patterns across the three chronological phases (Fig. 11), in the early Neolithic livestock management (caprines and cattle) is predominant only in a few sites, such as Grotta dell’Edera (99%) and Piancada (73%) in the Friulian plains^58,132–134^, Casalecchio di Reno (84%) and Casa Gazza (54%) in the southern Po plain^70,135^, and Lugo di Grezzana (84%) in the Garda–Venetian Prealps^136^. Hunting—especially of deer^68^—remained important in most other sites, with game frequencies ranging between 30 and 60%. This indicates a complex and locally variable transition to herding. Suids are well attested in this phase, particularly in the central Po plain (Ostiano–Dugali Alti, Isorella, Vho di Piadena), Casa Gazza (southern Po plain), and Cologna Veneta (Venetian plain), with frequencies of 25–35%, whereas their role is minor in the Adige Valley, southern Po plain, and Friulian plains (average 13%). During the middle Neolithic, animal husbandry becomes the dominant subsistence strategy almost everywhere (average > 70%). The only exceptions are Razza di Campegine (~ 9%)^137^ and Molino Casarotto (~ 4%)^62^, where clear evidence of wild boar hunting has been reported^104^. In these sites, wild game (mainly cervids) reaches 61–67%, though significant proportions are also recorded at Botteghino (27%)^138^ and Isera–La Torretta (~ 32%)^58,139^. This likely reflects opportunistic hunting strategies driven by specialization or local environmental conditions, such as the abundance or concentration of game in specific locations, which could make hunting more efficient in terms of energy investment and yield compared with livestock management^59^. Suids increase both in frequency and spatial distribution compared to the earlier phase: apart from five sites (mostly in the southern Po plain) showing low values (1–13%, mean = 8%), the remaining nine sites yield 19–35% (mean = 28%). During the late Neolithic, the pattern becomes less linear. In the Central Po Plain, hunting regained some relevance (15–28%), as also observed at Cornuda (48%)^140^, the only other case, besides Molino Casarotto, where suid remains may represent hunted wild boar^104^. Nevertheless, wild animals overall likely lost importance as meat providers due to agricultural expansion and habitat reduction^59^. These broader changes could have promoted a shift from a loose free-range system toward more intensive pig management (now ranging between 10 and 45%, mean = 28%), with closer control of herds. This likely caused selective morphological changes resulting in a clearer distinction between wild and domestic forms^104^ and indicating the growing economic importance of pigs within subsistence systems. Naturally, within this scenario, the exploitation of caprines and cattle remained central. Statistical analyses focusing on livestock-related taxa (Fig. 8, SI Table S8) reveal noteworthy patterns. General chronological trends (Fig. 8b) show that caprines were consistently important across all the phases, cattle were most prominent in the middle Neolithic, and suids gradually increased over time, with occasional sites showing particularly high suids abundance (i.e. Molino Casarotto, Vho di Piadena, Razza di Campegine and Cornuda) perhaps reflecting a bias related to wild boar hunting. The PCA (Fig. 8a) and the scatter plots (Fig. 8.c) highlight distinct livestock management patterns across northern Italy. In the Trieste karst, caprines dominate throughout all phases, indicating a strong focus on sheep and goats. In the remaining areas, mixed herding systems are observed, with all three species exploited but showing regional differences. In the southern Po plain, cattle are clearly predominant. In the central Po plain and in the Venetian plains and hills, caprines are generally less abundant, cattle more frequent, and suids predominant, nevertheless in the central plain, cattle surpass suids in the middle Neolithic and the two taxa become comparable in the later phase. In the Garda-Venetian Prealps and Adige valley, early Neolithic sites show lower cattle abundance compared to caprines and suids, whereas middle Neolithic sites display an almost even distribution among the three taxa. Finally, in the Friulian plain, the dataset is too limited to draw general conclusions.

Fishing, while never dominant, remained a persistent and complementary component of Neolithic subsistence in northern Italy, but the data must be interpreted cautiously, as they may be underestimated due to sampling biases and the limited recovery of small remains without fine sieving or flotation. Stepwise correlation analysis shows that ichthyofauna is negatively correlated with other animal categories, suggesting that fishing generally complemented hunting and herding rather than forming the primary subsistence base. Early Neolithic evidence in the central Po plain is limited to a few fish remains^65,70^, reflecting this general pattern. Exceptions are Piancada, where molluscs represent an important food source^132,133^, and especially Lugo di Romagna, where a richer assemblage of marine and freshwater fish, alongside molluscs, indicate that fishing played a fundamental role^96,141^. In the PCA combining faunal and botanical data, Lugo di Romagna occupies a distinct quadrant, highlighting the site’s unique agro-ichthyic economy (Fig. 7-LUG-ROM). In the middle Neolithic, freshwater fish are documented in the central Po Valley and the Venetian hills^142–144^, as well as at Molino Casarotto where pike, tench, and mollusc represent a conspicuous part of the faunal assemblage. Other notable assemblages are reported at Riva del Garda^145^ and, during the late Neolithic, at Tosina di Monzambano^146,147^. Rare fishing hooks suggest occasional tool use^148,149^, but their scarcity indicates simple, archaeologically elusive techniques, consistent with ethnographic evidence^150,151^. Overall, the available evidence depicts a dynamic and locally differentiated Neolithic economy in northern Italy, in which the agro-pastoral model had become more stable and consolidated than at its introduction. Hunting, fishing, mollusc collecting, and the gathering of nuts and fresh fruits remained significant, contributing to mixed economies that exploited all locally available resources. Each site adapted its subsistence strategies to local environmental conditions, selecting the most advantageous activities. Variability in subsistence economies is not fully explained by material culture; it appears more closely linked to environmental factors and community needs, though local non-economic choices—such as traditions, social preferences, symbolic norms, or innovations—may also have played a role. Despite overarching trends, this complexity resists rigid or predictable regional, cultural, or chronological patterns.

## Conclusion

The interdisciplinary study following the recent excavation campaign at the middle Neolithic site of Molino Casarotto offers an opportunity to rethink the concept of the Neolithic in northern Italy in a more flexible and nuanced way. Our results highlight the complexity of subsistence economies across this culturally and geographically diverse macro-region. While previous models of Neolithization often approached the adoption of agriculture and animal husbandry in a dichotomous manner, the present study reveals a spectrum of “mixed economies” in which farming, herding, hunting, fishing, and the gathering of wild fruits, nuts, and molluscs coexisted, with the relative importance of each activity varying between sites.

Statistical analyses, although showing a general trend towards a progressively more consolidated agro-pastoral economy over the course of the Neolithic, also underscore the persistence of foraging-oriented strategies. Sites such as Molino Casarotto exemplify outliers within this trend, highlighting the remarkable variability and flexibility of Neolithic subsistence traditions. The specific drivers behind this mosaic remain uncertain but likely include environmental conditions, economic opportunities, and possibly cultural factors. While our data cannot directly demonstrate cultural intent, the continued exploitation of wild resources may reflect adaptive resilience, helping communities maintain flexibility in resource knowledge and subsistence strategies.

Taken together, these results suggest that Neolithic communities in northern Italy adopted context-specific and adaptive strategies rather than following a single, uniform model of economic change. Future systematic archaeobotanical and archaeozoological research should aim to further disentangle the social, ecological, and cultural interactions that informed subsistence decisions, providing a more comprehensive understanding of the processes of neolithization in the region.

## Materials and methods

### Plant macro-remains

Sediment samples were collected from all excavated stratigraphic units. The number of samples collected from each SU varied according to its extent, defined on the excavation site by a specific grid of 100 × 100 cm, further subdivided into squares of 50 × 50 cm. In total, 99 samples were collected, amounting to 166,15 l. However, excluding the samples from SU potentially contaminated by recent peat extraction, a total of 71 stratigraphically reliable samples from 18 SU were used for this study, amounting to 133 l of analysed sediment (SI Table S2). The extraction of macro remains was carried out during archaeological excavation operations through flotation and water sieving using a flotation machine. The lighter plant material floated to the surface and was collected in a 0,3 mm mesh sieve, while the heavier, non-floating fraction was sieved under a water jet with a 1 mm mesh. Following this operation, the samples were transferred to the laboratory for sorting and identification conducted through observations with a Optika SZA1 stereomicroscope. The identification of seeds and fruit remains was performed by consulting seed atlases and specialized reference literature^84,152–157^.

For each taxon, the ubiquity value was measured based on both the samples and the SU analysed (SI Table S9). The reliability of the analysed assemblage was estimated following Diehl^55^ as very high: with 71 samples analysed, there is a 2.6% (*p* = 0.026) probability of missing taxa that represent less than 5% of the total assemblage present at the site (i.e. taxa with a theoretical ubiquity less than 0.05).

### Plant micro-remains

For the extraction of siliceous microfossils from the main stratigraphic units excavated at Molino Casarotto, the laboratory protocol for sample processing followed Madella et al.^158^, for details see SI Methods. For the calculation of acid insoluble fraction (AIF), the procedure follows Lancelotti^159^, adapted from Albert and Weiner^160^. Phytoliths nomenclature respects ICPN 2.0^92^. Seventeen sediment samples have been processed that came from eleven different stratigraphic units as in SI Table S4.

### Terrestrial faunal remains

Materials were collected through both archaeological excavation operations and the flotation and sieving of sediments. Unlike the faunal findings from previous archaeological research in the area^62,68^ the number of animal bones discussed here is relatively small and characterised by an extremely fragile and fragmentary state of preservation. Most of the remains feature bone flakes that, in fact, detach easily.

Bone identification was carried out using osteological atlases^161,162^ and the reference collection of the PrEcLab (Laboratory of Prehistory, Protohistory, and Ecology) at the Department of Cultural and Environmental Heritage, University of Milan. Measurements were performed following von den Driesch^163^. The distinction between domestic pig and wild boar was based on comparison with reference specimens and observations of available measurements (the humerus from SU 5/sq.C3-C4 shows the following measurements: Bd = 47,2 and SD = 20,7).

A total of 779 remains were examined, with a weight of 465 g. Only a small percentage of the assemblage could be anatomically and taxonomically determined due to the reasons stated above (4.6%, corresponding to 36 remains). The total number, however, is misleading. The size of the undetermined fragments is in fact, in most cases, less than a centimetre and it is likely that many of them belong to the same bone; this would greatly reduce the real number of recovered remains.

### Freshwater faunal remains

Archaeological sediments were wet sifted using mesh sizes of 1 and 2 mm. A total of 70 samples were sifted with the 1 mm mesh, and 26 samples with the 2 mm mesh. The 1 mm samples amounted to 162,08 l of soil, while the 2-mm samples totalled 154,5 l. No significant difference was observed between the faunal materials recovered from the two mesh sizes; therefore, the faunal sample will be discussed as a whole.

Fish bones and mollusc shells have been identified by using works from different specialists as reference, namely: Wilkens^164^, Lazzari^165^ for the latter, Libois & Hallet Libois^166^, Radu^167^, Davis et al.^168^ for the former. For information regarding ecology and species distribution, Bruno & Maugeri^169^ was used as reference for fish and Cossignani & Cossignani^170^ for molluscs.

The Number of Identified Specimens (NISP) was calculated for all *taxa*, while the Minimum Number of Individuals (MNI) was only calculated for molluscs following Girod^95^. Fish bones measurements were recorded using the criteria described in Morales & Rosenlund^171^. The Total Length (TL) and total weight of pike were calculated following Frezza^172^ for cranial remains and Jelu et al.^173^ for vertebrae.

### Statistical analysis

An integrated statistical data analysis was performed on the compositional dataset—i.e., normalized percentages of different classes of plant and animal remains—from 53 assemblages belonging to 49 sites. These sites are distributed across seven geographical areas of northern Italy: Trieste Karst, Friulian plain, Venetian hills and plain, Garda-Venetian Prealps and Adige valley, Varese Prealps, central Po plain, and southern Po plain, and cover the early, middle, and late Neolithic phases (Figs. 1, 2).

To minimize biases related to different sampling intensities, data were normalized by compositional proportion. For simplicity in visualization and comparison, the following macrocategories were defined: crops (cereals and legumes), nuts (hazelnuts, acorns, and beech nuts), and fresh fruits (including water chestnuts) as plant remains; and domesticates (caprines and cattle), suids (both pigs and wild boars), game (cervids and other mammals, birds, reptiles, and amphibians), and ichthyofauna (freshwater and marine fish and mollusks) as animal remains (SI Table S8).

The dataset was pre-processed using a centered log-ratio (CLR) transformation to avoid the data closure effect. The statistical workflow included: (1) univariate analysis based on boxplots and scatter plots; (2) stepwise correlation analysis; and (3) principal component analysis (PCA) using the covariance matrix. The PCA was conducted separately for sites with (a) plant remains, (b) animal remains, and (c) both combined. A fourth PCA was performed on log-ratios of caprine, cattle, and suid remains to explore livestock husbandry patterns. All calculations were performed using Statgraphics Centurion 19. This approach allowed us to explore possible chronological and geographical trends in the relative abundance of archaeological finds and to identify statistical patterns linking the distribution of plant and animal materials.

## Supplementary Information

Below is the link to the electronic supplementary material.

Supplementary Material 1

## Data Availability

All data generated from this research are available in the main text and in the supplementary information.

## References

[1] Lubbock, J. *Pre-Historic Times as Illustrated by Ancient Remains and the Manners and Customs of Modern Savages* (William and Norgate, 1865).

[2] Whitehouse, R. D. Siticulosa Apulia revisited. *Antiquity* **60**, 36–44 (1986).

[3] Zvelebil, M. On the transition to farming in Europe, or what was spreading with the neolithic: a reply to Ammerman (1989). *Antiquity* **63**, 379–383 (1989).

[4] Zohary, D., Hopf, M. & Weiss, E. *Domestication of Plants in the Old World: the Origin and Spread of Domesticated Plants in Southwest Asia, Europe, and the Mediterranean Basin* (Oxford University Press, 2013).

[5] Arranz-Otaegui, A. & Roe, J. Revisiting the concept of the ‘Neolithic founder crops’ in Southwest Asia. *Veg. Hist. Archaeobot.* **32**, 475–499 (2023).

[6] Martin, L., Russell, N., Carruthers, D., Gérard, F. & Thissen, L. Animal Remains from the Central Anatolian Neolithic. In: *The Neolithic of Central Anatolia: Internal Developments and External Relations during the 9th-6th Millennia CAL BC: Proceedings of the International CANeW Table Ronde, Istanbul, 23–24 November* 193–206 (Ege Yayinlari, Istanbul, 2002). 193–206 (Ege Yayinlari, Istanbul, 2002).

[7] Chapman, J. & Müller, J. Early farmers in the mediterranean basin: the dalmatian evidence. *Antiquity* **64**, 127–134 (1990).

[8] Thomas, J. *Rethinking the Neolithic* (Cambridge University Press, 1991).

[9] Pluccienik, M. Deconstructing ‘The neolithic’ in the Mesolithic-Neolithic transition. In: *Understanding the Neolithic of North-Western Europe* (eds Edmonds, M. & Richards) C.) 61–83 (Cruithne Press 1998).

[10] Budja, M. The transition to farming in mediterranean Europe—an indigenous response. *Doc. Praehist.* **26**, 119–141 (1999).

[11] *Europe’s First Farmers* (Cambridge University Press, 2000). 10.1017/CBO9780511607851.

[12] Tringham, R. The continuous house. A view from the deep past. In *Beyond kinship. Social and Material Reproduction in House Societies* (eds Joyce, R. A. & Gillespie, S. D.) 115–134 (University of Pennsylvania, 2000).

[13] Zvelebil, M. & Lillie, M. Transition to agriculture in Eastern Europe. In *Europe’s First Farmers* (ed. Price, T. D.) 57–92 (Cambridge University Press, 2000). 10.1017/CBO9780511607851.004.

[14] Kotsakis, K. Mesolithic to neolithic in Greece. Continuity, discontinuity or change of course? *Doc. Praehist.* **28**, 63–73 (2001).

[15] Gehlen, B. & Schön, W. Das „Spätmesolithikum und Das initiale neolithikum in Griechenland—Implikationen für die neolithisierung der Alpinen und circumalpinen gebiete. *Archäol. Inf.* **26**, 255–273 (2003).

[16] Çilingiroğlu, Ç. The concept of neolithic package: considering its meaning and applicability. *Doc. Praehist.* **32**, 1–13 (2005).

[17] Brami, M. Anatolia. From the origins of agriculture… to the spread of Neolithic economies. In *The Central/Western Anatolian farming frontier* (eds. Brami, M. & Horejs, B.) 17–44 (Austrian Academy of Sciences Press, 2019). 10.2307/j.ctvvh866f.5.

[18] Lipson, M. et al. Parallel palaeogenomic transects reveal complex genetic history of early European farmers. *Nature* **551**, 368–372 (2017). 29144465 10.1038/nature24476 PMC5973800

[19] Furtwängler, A. et al. Ancient genomes reveal social and genetic structure of late neolithic Switzerland. *Nat. Commun.* **11**, 1915 (2020). 32313080 10.1038/s41467-020-15560-x PMC7171184

[20] Sikora, M. et al. Population genomic analysis of ancient and modern genomes yields new insights into the genetic ancestry of the Tyrolean Iceman and the genetic structure of Europe. *PLoS Genet.* **10**, e1004353 (2014). 24809476 10.1371/journal.pgen.1004353 PMC4014435

[21] Mathieson, I. et al. Genome-wide patterns of selection in 230 ancient Eurasians. *Nature* **528**, 499–503 (2015). 26595274 10.1038/nature16152 PMC4918750

[22] Chiang, C. W. K. et al. Population history of the Sardinian people inferred from whole-genome sequencing. **092148** (2016). 10.1101/092148

[23] Hofmanová, Z. et al. Early farmers from across Europe directly descended from neolithic Aegeans. *Proc. Natl. Acad. Sci.* **113**, 6886–6891 (2016). 27274049 10.1073/pnas.1523951113 PMC4922144

[24] Sarno, S. et al. Ancient and recent admixture layers in Sicily and Southern Italy trace multiple migration routes along the mediterranean. *Sci. Rep.* **7**, 1984 (2017). 28512355 10.1038/s41598-017-01802-4 PMC5434004

[25] Raveane, A. et al. Population structure of modern-day Italians reveals patterns of ancient and archaic ancestries in Southern Europe. *Sci. Adv.* **5**, eaaw3492 (2019). 31517044 10.1126/sciadv.aaw3492 PMC6726452

[26] Fontani, F. et al. Bioarchaeological and paleogenomic profiling of the unusual neolithic burial from Grotta Di Pietra sant’angelo (Calabria, Italy). *Sci. Rep.* **13**, 11978 (2023). 37488251 10.1038/s41598-023-39250-y PMC10366206

[27] Antonio, M. L. et al. Ancient rome: a genetic crossroads of Europe and the mediterranean. *Science* **366**, 708–714 (2019). 31699931 10.1126/science.aay6826 PMC7093155

[28] Omrak, A. et al. Genomic evidence establishes Anatolia as the source of the European neolithic gene pool. *Curr. Biol.* **26**, 270–275 (2016). 26748850 10.1016/j.cub.2015.12.019

[29] Posth, C. et al. Palaeogenomics of upper palaeolithic to neolithic European hunter-gatherers. *Nature* **615**, 117–126 (2023). 36859578 10.1038/s41586-023-05726-0 PMC9977688

[30] Bagolini, B. & Broglio, A. Il Ruolo Delle alpi Nei tempi preistorici (dal paleolitico al Calcolitico). In *Studi Di Paletnologia in Onore Di Salvatore M. Pugliesi* (eds Liverani, M. et al.) 663–705 (Università di Roma ‘La Sapienza,’ 1985).

[31] Barker, G., Biagi, P., Castelletti, L., Cremaschi, M. & Nisbet, R. Sussistenza, economia ed ambiente nel Neolitico dell’italia settentrionale. In *Atti Della XXVI Riunione Scientifica Il Neolitico in Italia* (Istituto Italiano di Preistoria e Protostoria, (1987).

[32] Broglio, A. & Lanzinger, M. Considerazioni Sulla distribuzione dei Siti Tra La fine Del paleolitico superiore e l’inizio Del Neolitico nell’italia nord-orientale. In *The neolithisation of the alpine region*. (ed. Biagi P) 53–69 (1990).

[33] Clark, R. The beginnings of agriculture in sub-alpine Italy: some theoretical considerations. In *The neolithisation of the Alpine region* (ed. Biagi, P.) 123–137 (1990).

[34] Riedel, A. Remarks on some neolithic faunas of north-eastern Italy and on the neolithisation process. In *The neolithisation of the Alpine region* (ed. Biagi, P.) 139–146 (1990).

[35] Starnini, E., Biagi, P. & Mazzucco, N. The beginning of the neolithic in the Po plain (northern Italy): problems and perspectives. *Quat. Int.* **470**, 301–317 (2018).

[36] Carugati, M. G., Castelletti, L. & Rottoli, M. L’agricoltura nel primo neolitico nel Friuli. Le ricerche a Sammardenchia, Fagnigola e Valer. In *Sammardenchia e i primi agricoltori del Friuli* (eds. Ferrari, A. & Pessina, A.) 103–112 (1996).

[37] Castelletti, L. & Maspero, A. Analisi Di resti vegetali Di Campo ceresole Del Vhò Di Piadena e Di Altri Siti neolitici Padani. *Nat. Brescia*. **27**, 289–305 (1992).

[38] Castelletti, L. & Carugati, M. G. I resti vegetali del sito neolitico di Sammardenchia di Pozzuolo del Friuli (Udine). In *Preistoria e protostoria del Friuli-Venezia Giulia e dell’Istria. Atti della XXIX Riunione scientifica (Trieste, 28–30 settembre*) 167–183 (Istituto Italiano di Preistoria e Protostoria, Firenze, 1994).) 167–183 (Istituto Italiano di Preistoria e Protostoria, Firenze, 1994). (1990).

[39] Rottoli, M. & Pessina, A. Routledge, Neolithic agriculture in italy: an update of archaeobotanical data with particular emphasis on Northern settlements. In *The Origins and Spread of Domestic Plants in Southwest Asia and Europe* (eds Colledge, S. & Conolly) J.) (2007).

[40] Valsecchi, V., Finsinger, W., Tinner, W. & Ammann, B. Testing the influence of climate, human impact and fire on the holocene population expansion of fagus sylvatica in the Southern prealps (Italy). *Holocene* **18**, 603–614 (2008).

[41] Marchesini, M., Carra, M., Marvelli, S. & Rizzoli, E. Paesaggio vegetale e Sussistenza in Emilia orientale e Romagna nell’età Del Bronzo. In *Preistoria E Protostoria dell’Emilia Romagna* Vol. 2 (ed. Brea, B.) 133–144 (Istituto Italiano di Preistoria e Protostoria, 2018).

[42] Biagi, P., Starnini, E., Borić, D. & Mazzucco, N. Early neolithic settlement of the Po plain (Northern Italy): Vhò and related sites. *Doc. Praehist.* **47**, 192–221 (2020).

[43] Becker, V. La Dolce vita. The early neolithic in Northern Italy and its connections to southeastern Europe. In *From Farmers To Heroes? Archaeological Studies in Honor of Slawomir Kadrow* Vol. 376 (eds Debiec, M. et al.) 17–31 (Dr. Rudolf Habelt, 2022).

[44] Barfield, L. H. Neolithic and Copper Age Flint Exploitation in Northern Italy. in *Prehistoric alpine environment, society and economy, Papers of the international colloquium* (ed. Della Casa, P.) 245–252 (Zurich, 1999).

[45] Barfield, L. H. Commercio e scambio nel Neolitico dell’italia settentrionale. In *La Neolitizzazione Tra Oriente E Occidente: Atti Del Convegno Di studi, Udine 23–24 Aprile 1999* (eds Muscio, G. & Pessina, A.) 55–66 (Edizioni del Museo Friulano di Storia Naturale, 2000).

[46] Biagi, P. & D’Amico, C. The greenstone tools from the middle neolithic site of Fimon and Villa del Ferro in the Berici Hills (Vicenza, northern Italy). *Atti Della Soc. Preistoria E Protostoria Della Reg. Friuli-Venezia Giulia* XVIII, 87–105 (2013).

[47] Ferrari, A. & Mazzieri, P. Fonti e processi di scambio di rocce silicee scheggiabili. In *Settemila anni fa… il primo pane. Ambienti e culture delle società neolitiche* (eds. Pessina, A. & Muscio, G.) 165–169 (Museo Friulano di Storia Naturale, 1999).

[48] Pessina, A. Il primo Neolitico dell’italia settentrionale: problemi generali. In *La Neolitizzazione Tra Oriente E Occidente: Atti Del Convegno Di studi, Udine 23–24 Aprile 1999* (eds Muscio, G. & Pessina, A.) 55–66 (Edizioni del Museo Friulano di Storia Naturale, 2000).

[49] Ferrari, A. Esordi e stabilizzazione Della cultura dei Vasi a Bocca quadrata Di Stile geometrico-lineare. In *Vasi a Bocca quadrata. Evoluzione Delle conoscenze Nuovi approcci Interpretativi*. (ed. Mottes E) 51–70 (2021).

[50] Tirabassi, I. Revisione dei Vecchi ritrovamenti VBQ e analisi dei Nuovi. Verso Una sintesi per Il reggiano. In *Vasi a Bocca Quadrata. Evoluzione Delle conoscenze Nuovi approcci Interpretativi*. (ed. Mottes E) 71–92 (2021).

[51] Baioni, M., Lo Vetro, D. & Poggiani Keller, R. Aggiornamenti Su Siti e materiali VBQ Della Lombardia settentrionale. In *Vasi a Bocca Quadrata. Evoluzione Delle conoscenze Nuovi approcci Interpretativi*. (ed. Mottes E) 93–130 (2021).

[52] Tiné, V. Il Neo­litico in Veneto. In *Preistoria e Protostoria del Veneto* (eds. Leonardi, G. & Tiné, V.) 73–94 (Istituto Italiano di Preistoria e Protostoria, Soprintendenza per i beni archeologici del Veneto, Università degli studi di Padova, Firenze, Padova, (2015).

[53] Pedrotti, A., Keller, P., Banchieri, R., Longhi, C. & D. & Il Neolitico in Lombardia. *Riv Sci. Preistoriche*. **LXXII**, 129–172 (2022).

[54] Bernabò Brea, M., Miari, M. & Steffè, G. Il Neolitico dell’emilia Romagna. in Preistoria E Protostoria dell’Emilia Romagna vol. 1 119–137 (Firenze, (2017).

[55] Diehl, M. W. Paleoethnobotanical sampling adequacy and ubiquity: an example from the American Southwest. *Adv. Archaeol. Pract.* **5**, 196–205 (2017).

[56] Castelletti, L. & Rottoli, M. New data on neolithic agriculture and environment in Northern Italy. *Preist. Alp.* **33**, 57–61 (1997).

[57] Rottoli, M. & Castiglioni, E. Prehistory of plant growing and collecting in Northern Italy, based on seed remains from the early neolithic to the chalcolithic (c. 5600–2100 cal B.C). *Veg. Hist. Archaeobot.* **18**, 91–103 (2009).

[58] Tecchiati, U. et al. Zooarchaeological evidence of functional and social differentiation in Northern Italy between the neolithic and bronze ages. *Quat. Int.* **539**, 105–121 (2020).

[59] Zanetti, A. L., Fontana, A. & Tecchiati, U. Osservazioni Su Ruolo e significato Degli animali selvatici nel Neolitico e nell’Età Del Rame Dell’Italia Nordorientale Alla Luce Dell’archeozoologia. *Preist. Alp.* **50**, 89–100 (2020).

[60] Evett, D. & Renfrew, J. L’agricoltura neolitica italiana: Una nota Sui cereali. *Riv Sci. Preist.* **26**, (1971).

[61] Jarman, M. Culture and economy in the North Italian neolithic. *World Archaeol.* **2**, 255–265 (1971).

[62] Jarman, M. Prehistoric economic development in sub-Alpine Ltaly. in Problems in Economic and Social Archaeology (eds Sieveking, G., de Longworth, G. & Wilson, K. E.) I. H. 523–548 (Gerald Duckworth & Co. Ltd, (1976).

[63] Jarman, M., Bailey, G. N. & Jarman, H. N. *Early Eur. Agric. Its Foundation Development* (1982).

[64] Guerreschi, A., Catalani, P. & Ceschin, N. Belforte Di Gazzuolo (Mantova). Una stazione Con Vasi a Bocca quadrata Del Neolitico superiore. *Preistoria Alp.* **22**, 35–118 (1986).

[65] Clark, G. Museo Civico di Scienze Naturali di Brescia,. Early neolithic subsistence. In *L’insediamento neolitico di Ostiano-Dugali Alti (Cremona) nel suo contesto ambientale ed economico* (ed. Biagi, P.) 99–103 (1995).

[66] Starnini, E., Ghisotti, F., Girod, A. & Nisbet, R. Nuovi Dati Sul Neolitico Antico Della Pianura Padana centrale Dal Sito Di Isorella (Brescia). In *La Neolitizzazione Tra Oriente E Occidente: Atti Del Convegno Di studi, Udine, 23–24 Aprile 1999* (eds Pessina, A. & Muscio, G.) 231–255 (Museo Friulano di Storia Naturale, 2000).

[67] Bon, M., Zampieri, S. & Starnini, E. La fauna del pozzetto neolitico di Isorella (BS). In *Atti del 4° Convegno Nazionale di Archeozoologia, Pordenone, 13–15 Novembre 2003* 177–182 (Museo archeologico del Friuli occidentale, Pordenone, 2005).

[68] Boyle, K. The Italian neolithic red deer: Molino Casarotto. In *Deer and People* (eds Baker, K. et al.) 78–91 (Windgather, 2014). 10.2307/j.ctv13gvgms.11.

[69] Girod, A. & Starnini, E. Mollusc assemblage from the early neolithic site of Isorella (BS, Northern Italy) and its palaeoenvironmental significance. *J. Archaeol. Sci. Rep.* **43**, 103489 (2022).

[70] Messana, C., Bernabò Brea, M. & Bertolini, M. & Thun Hohenstein, U. Faunal exploitation in an Early Neolithic site: the assemblage from Casa Gazza (Travo, Piacenza, Northern Italy). In *Neolithic and Bronze Age studies in Europe. From material culture to territories* vol. 13, 22–30 (Archaeopress Publishing LTD, Oxford, 2021).

[71] Antolín, F. & Jacomet, S. Wild fruit use among early farmers in the neolithic (5400–2300 cal bc) in the north-east of the Iberian peninsula: an intensive practice? *Veg. Hist. Archaeobot.* **24**, 19–33 (2015).

[72] Hosch, S. & Jacomet, S. Ackerbau und Sammelwirtschaft. Ergebnisse der Untersuchung von Samen und Früchten. In *Die jungsteinzeitliche Seeufersiedlung Arbon Bleiche 3. Umwelt und Wirtschaft.* (eds. Jacomet, S., Leuzinger, U. & Schibler, J.) 112–157 (Amt für Archäologie des Kanton Thurgau, Frauenfeld, (2004).

[73] Jacomet, S. Plant economy and village life in neolithic lake dwellings at the time of the alpine Iceman. *Veg. Hist. Archaeobot.* **18**, 47–59 (2009).

[74] Tolar, T., Jacomet, S., Velušček, A. & Čufar, K. Plant economy at a late neolithic lake dwelling site in Slovenia at the time of the alpine Iceman. *Veg. Hist. Archaeobot.* **20**, 207–222 (2011).

[75] Kirleis, W., Klooß, S., Kroll, H. & Müller, J. Crop growing and gathering in the Northern German neolithic: a review supplemented by new results. *Veg. Hist. Archaeobot.* **21**, 221–242 (2012).

[76] Bouby, L. et al. Talkin’ about a Revolution. Changes and continuities in fruit use in Southern France from neolithic to Roman times using archaeobotanical data (ca. 5,800 BCE—500 CE). *Front Plant. Sci.* **13**, (2022). 10.3389/fpls.2022.719406 PMC8859487 35197992

[77] Pessina, A. & Tiné, V. *Archeologia Del Neolitico L’Italia Tra VI e IV Millennio.* Carocci Editore, (2022).

[78] Bagolini, B., Barfield, L. H. & Broglio, A. Notizie preliminari Delle ricerche sull’insediamento Neolitico Di Fimon-Molino Casarotto (Vicenza). *Riv Sci. Preist.* **28**, 161–215 (1973).

[79] Nicosia, C. et al. Nuovi scavi presso Il Sito Del Neolitico medio Di Molino Casarotto Nelle Valli Di Fimon (Arcugnano, Vicenza). *Preist. Alp.* **55**, 53–66 (2025).

[80] Barfield, L. H., Broglio, A., Durante Pasa, M. V. & Magaldi, D. I depositi. In *L’insediamento Neolitico Di Molino Casarotto Nelle Valli Di Fimon (Colli Berici, Vicenza)* (eds Barfield, L. & Broglio, A.) 57–72 (Accademia Olimpica, 1986).

[81] Broglio, A. & Fogolari, G. «Molino Casarotto» Nella Valle Di Fimon (Com. Di Arcugnano, Provo Di Vicenza). *Riv Sci. Preist.* **25**, 413–414 (1970).

[82] Barfield, L. H. & Broglio, A. (eds.)* L’insediamento neolitico di Molino Casarotto nelle valli di Fimon (Colli Berici, Vicenza)* (Accademia Olimpica, 1986).

[83] Nicosia, C., Dal Sasso, G. & Polisca, F. Cooking, cleaning, and tossing: high-resolution analysis of domestic activities at the mid-Neolithic site of Molino Casarotto (Vicenza, NE Italy). *Archaeol Anthropol. Sci.*10.1007/s12520-025-02353-w (2025). 10.1007/s12520-025-02353-w PMC12698799 41393176

[84] Jacomet, S. Identification of cereal remains from archaeological sites. (2006).

[85] Heiss, A. G. et al. State of the (t)art. Analytical approaches in the investigation of components and production traits of archaeological bread-like objects, applied to two finds from the neolithic lakeshore settlement parkhaus Opéra (Zürich, Switzerland). *PLoS One*. **12**, e0182401 (2017). 28771539 10.1371/journal.pone.0182401 PMC5542691

[86] Heiss, A. G. et al. Mashes to mashes, crust to crust. Presenting a novel microstructural marker for malting in the archaeological record. *PLoS One*. **15**, e0231696 (2020). 32379784 10.1371/journal.pone.0231696 PMC7205394

[87] Ball, T. et al. Phytoliths as a tool for investigations of agricultural origins and dispersals around the world. *J. Archaeol. Sci.* **68**, 32–45 (2016).

[88] Dal Corso, M. *Environmental History and Development of the Human Landscape in a Northeastern Italian Lowland during the Bronze Age: A Multidisciplinary Case-Study* (Habelt, 2018).

[89] Madella, M., García-Granero, J. J., Out, W. A., Ryan, P. & Usai, D. Microbotanical evidence of domestic cereals in Africa 7000 years ago. *PLoS One*. **9**, e110177 (2014). 25338096 10.1371/journal.pone.0110177 PMC4206403

[90] Out, W. A. et al. Plant use at funnel beaker sites: combined macro- and microremains analysis at the early neolithic site of Frydenlund, Denmark (ca. 3600 BCE). *Veg. Hist. Archaeobot.* 1–27. 10.1007/s00334-024-01020-9 (2024).

[91] Shillito, L. M. Simultaneous thin section and phytolith observations of finely stratified deposits from neolithic Çatalhöyük, turkey: implications for paleoeconomy and early holocene paleoenvironment. *J. Quat. Sci.* **26**, 576–588 (2011).

[92] International Committee for Phytolith Taxonomy. International code for phytolith nomenclature (ICPN) 2.0. *Ann. Bot.* **124**, 189–199 (2019). 31334810 10.1093/aob/mcz064 PMC6758648

[93] Shipman, P., Foster, G. & Schoeninger, M. Burnt bones and teeth: an experimental study of color, morphology, crystal structure and shrinkage. *J. Archaeol. Sci.* **11**, 307–325 (1984).

[94] Marrone, F. et al. Diversity and taxonomy of the genus Unio Philipsson in Italy, with the designation of a neotype for Unio elongatulus C. Pfeiffer (Mollusca, Bivalvia, Unionidae). *Zootaxa* **4545**, 339–374 (2019). 30790905 10.11646/zootaxa.4545.3.2

[95] Girod, A. Potenziale informativo dei molluschi marini, d’acqua Dolce e terrestri in archeologia. In *Appunti Di Archeomalacologia* (ed. Girod, A.) 11–128 (All’Insegna del Giglio, 2015).

[96] Albertini, D. & Tagliacozzo, A. I resti Di ittiofauna. In *Il Villaggio Neolitico Di Lugo Di Romagna—Fornace Gattelli. Strutture, Ambiente, Culture* (eds Steffè, G. & Degasperi, N.) 351–356 (Istituto Italiano di Preistoria e Protostoria, 2019).

[97] Simões, C. D. & Aldeias, V. Thermo-microstratigraphy of shells reveals invisible fire use and possible cooking in the archaeological record. *Front. Earth Sci.* **10**, (2022).

[98] Karg, S. The water chestnut (Trapa natans L.) as a food resource during the 4th to 1st millennia BC at lake Federsee, bad Buchau (southern Germany). *Environ. Archaeol.* **11**, 125–130 (2006).

[99] Reed, K. Archaeobotanical analysis of Bronze Age Feudvar. in *Die Archäobotanik. Feudvar III.* (eds. Kroll, H. & Reed, K.) 200–291 (Würzburg University Press, 2016).

[100] Borojević, K. Water chestnuts (*Trapa natans* L.) as controversial plants: botanical, ethno-historical and archaeological evidence. In *From Foragers To farmers. Gordon C. Hillman Festschrift* (eds Fairbairn, A. S. & Weiss, E.) 86–97 (OxBow Books, 2012).

[101] Piper, L. Born to be wild? The Problem with Pigs in the North Italian Neolithic: a re-analysis of the animal bone assemblage from Rocca di Rivoli. In *Alle Origini del Territorio di Rivoli. Contributi alla scoperta della paleontologia e archeologia di Rivoli Veronese, Atti della Giornata di Studi—17 maggio 2008* (ed. Dalla Riva, M.) 31–46 (Redaprint, Cavaion Veronese, 2010).

[102] Bazzanella, M. The fauna of La Vela Di trento: preliminary analysis. *Preist. Alp.* **34**, 307–310 (2001).

[103] Barker, G. Early neolithic economy at Vhò. *Preist. Alp.* **12**, 61–70 (1976).

[104] Tecce, S. *The Origins and Evolution of Pig Domestication in Italy: A Regional and Diachronic Study of Husbandry Practices* (British Archaeological Reports Oxford Ltd, 2020).

[105] Franco, C. La fine Del mesolitico in italia: identità culturale e distribuzione territoriale Degli ultimi cacciatori-raccoglitori. in Quaderni-Società Per La Preistoria E Protostoria Della Regione Friuli Venezia Giulia (ed Biagi, P.) (Società per la preistoria e protostoria della Regione Friuli Venezia Giulia, 2011).

[106] Bagolini, B. *Il Trentino Nella Preistoria Del Mondo Alpino, Dagli Accampamenti Sotto Roccia Alla Città Quadrata* (Temi, 1980).

[107] Lunz, R. *Vor-und Frühgeschichte Südtirols. Steinzeit*. vol. 1 (1986).

[108] Broglio, A., Favero, V. & Marsale, S. Ritrovamenti mesolitici Attorno Alla Laguna Di Venezia. *Atti Ist Veneto Sci. Lett. E Arti*. **X**, 195–231 (1987).

[109] Lanzinger, M. Sistemi Di Insediamento mesolitico come Adattamento Agli Ambienti Montani alpini. In *The Mesolithic—Colloquium XIII: Formation of the European Mesolithic complexes. Colloquium XIV: Adaptations To Postglacial Environments* (eds Kozlowski, S. K. & Tozzi) 125–140 (C, 1996).

[110] Peresani, M., Perrone, R. & Zangheri, P. Insediamenti mesolitici Nella Valcalaona (Colli Euganei). *Archeol. Ven.* **XXIII**, 7–22 (2000).

[111] Peresani, M. & Contesti Risorse e variabilità Della presenza Umana nel paleolitico e nel mesolitico Nei Colli Euganei. *Preist. Alp.* **47**, 109–122 (2013).

[112] Cristiani, E. & Borić, D. Appearance and function of harpoons in Northeastern Italy. In *Hunter-Gatherers’ Tool-Kit: A Functional Perspective* (eds Gibaja, J. et al.) 2–27 (Cambridge Scholars Publishing, 2020).

[113] de Vareilles, A. et al. One sea but many routes to Sail. The early maritime dispersal of neolithic crops from the Aegean to the Western mediterranean. *J. Archaeol. Sci. Rep.* **29**, 102140 (2020).

[114] Kohler-Schneider, M. Contents of a storage pit from late bronze age Stillfried, austria: another record of the ‘new’ glume wheat. *Veg. Hist. Archaeobot.* **12**, 105–111 (2003).

[115] Carra, M. *Per Una Storia Della Cerealicoltura in Italia Settentrionale Dal Neolitico all’Età Del Ferro: Strategie Adattive E Condizionamenti Ambientali* (Alma Mater Studiorum-Università di Bologna, 2012).

[116] Rottoli, M. Analisi archeobotaniche: agricoltura, raccolta e Uso Del Legno. In *Il Villaggio Neolitico Di Lugo Di Romagna—Fornace Gattelli. Strutture, Ambiente, Culture* (eds Steffè, G. & Degasperi, N.) 315–337 (Istituto Italiano di Preistoria e Protostoria, 2019).

[117] Gobbo, I. Archeobotanica di siti mesolitici, neolitici ed eneolitici di pianura dell’Emilia Romagna. (Università degli Studi di Ferrara, (2011).

[118] Rottoli, M. I resti vegetali Di Sammardenchia-Cȗeis, Insediamento Del Neolitico Antico. In *Sammardenchia-Cueis. Contributi Per La Conoscenza Di Una comunità Del Primo Neolitico* (eds Ferrari, A. & Pessina, A.) 307–326 (Museo Friulano di Storia Naturale, 1999).

[119] Carugati, M. G. Il Neolitico Antico in Friuli attraverso lo studio dei resti vegetali carbonizzati di tre siti. Fagnigola (PN), Valer (PN) e Sammardenchia (UD). *Quad. Friulani Archeol.* III, 15–22 (1993).

[120] Rottoli, M., Cavulli, F. & Pedrotti, A. L’agricoltura Di Lugo Di Grezzana (Verona): considerazioni preliminari. In *Preistoria E Protostoria Del Veneto* Vol. 1 159–168 (Istituto Italiano di Preistoria e Protostoria, 2015).

[121] Mottes, E. & Rottoli, M. I resti carpologici Del Sito Neolitico de La Vela Di Trento (campagne Di scavo 1975 e 1976). In *Preistoria Dell’Italia settentrionale. Studi in Ricordo Di Bernardino Bagolini Atti Del Convegno, Udine 23–24 Settembre 2005* Vol. 53 (ed. Pessina, A.) 129–142 (Museo Friulano Storia Naturale, 2006).

[122] Steiner, B. L., Antolín, F., Soteras, R., Rottoli, M. & Banchieri, D. G. Isolino Virginia (Lake Varese, Italy): New archaeobotanical research at the earliest pile-dwelling of the circumalpine area. in *Prehistoric wetland sites of southern Europe: Archaeology, dendrochronology, palaeoecology and bioarchaeology* (eds. Ballmer, A., Tinner, W. & Hafner, A.) 267–282 (Springer Nature Switzerland, Cham, 2025). 10.1007/978-3-031-52780-7_16

[123] Banchieri, D. G., Bini, A., Rottoli, M. & Mainberger, M. Le Prealpi varesine e l’alimentazione durante la preistoria. In *Preistoria del cibo. L’alimentazione nella preistoria e nella protostoria* vol. 1 193–202 (2021).

[124] Micheli, R. et al. Nuove ricerche al Palù Di livenza: Lo scavo Del settore 3. In *Preistoria E Protostoria del. Caput Adriae* 481–490 (2018).

[125] Cottini, M. & Rottoli, M. I carboni di legna e le piante coltivate. In *Bannia—Palazzine di Sopra. Una comunità preistorica del V millennio a.C.* (ed. Visentini, P.) 129–145 (2005).

[126] Castiglioni, E. & Rottoli, M. I resti carpologici dall’abitato di Tosina. In *Contadini, allevatori e artigiani a Tosina di Monzambano (Mn) tra V e IV millennio a.C. Una comunità neolitica nei circuiti padani e veneti* (ed. Poggiani Keller, R.) 157–166 (2014).

[127] Vigne, J. D. Exploitation des animaux et néolithisation en Méditerranée nord-occidentale. In *Pont de Roque-Haute (Portiragnes, Hérault).Nouveaux regards sur la néolithisation de la France méditerranéenne* (eds. Guilaine, J., Manen, C. & Vigne, J.-D.) 221–278 (Archives d’écologie préhistorique, Toulouse, 2007).

[128] Albarella, U., Tagliacozzo, A., Dobney, K. & Rowley-Conwy, P. Pig hunting and husbandry in Prehistoric Italy: a contribution to the domestication debate. *Proc. Prehist. Soc.* **72**, 193–227 (2006).

[129] Larson, G. et al. Worldwide phylogeography of wild Boar reveals multiple centers of pig domestication. *Science* **307**, 1618–1621 (2005). 15761152 10.1126/science.1106927

[130] Larson, G. et al. Ancient DNA, pig domestication, and the spread of the Neolithic into Europe. *Proc. Natl. Acad. Sci.* **104**, 15276–15281 (2007). 10.1073/pnas.0703411104 PMC1976408 17855556

[131] Vigne, J. D. *Les mammifères post-glaciaires De Corse. Étude archéozoologique* Vol. 1 (Persée-Portail des revues scientifiques en SHS, 1988).

[132] Petrucci, G., Riedel, A. & Pessina, A. La fauna Del Canale Neolitico Di Piancada (UD). In *Atti Del 2° Convegno Nazionale Di Archeozoologia, Asti, 14–16 Novembre 1997* 193–200 (ABACO, 2000).

[133] Petrucci, G. & Riedel, A. La fauna di Piancada nell’ambito dell’archeozoologia dell’Italia nordorientale. In *Sammardenchia e i primi agricoltori del Friuli* (eds. Ferrari, A. & Pessina, A.) 113–120 (1996).

[134] Petrucci, G. Resti Di fauna Dai Livelli neolitici e post-neolitici Della Grotta Del Mitreo nel Carso Di Trieste (scavi 1967). In *Atti Della Società Per La Preistoria E La Protostoria Del Friuli-Venezia Giulia* Vol. 10 99–118 (Edizioni Svevo, 1996).

[135] Thun Hohenstein, U., Abuhelaleh, B., Petrucci, G. & Steffè, G. La gestione Delle risorse animali in Un Sito Del Neolitico antico: risultati preliminari Dello studio archeozoologico Delle faune Di Casalecchio Di Reno (Bologna). In *Atti Del 6° Convegno Nazionale Di Archeozoologia, Lucca, 21–24 Maggio 2009* (eds De Grossi Mazzorin, J. et al.) 163–166 (Associazione Italiana di Archeozoologia, 2012).

[136] Maccarinelli, A., Marconi, S. & Pedrotti, A. I resti faunistici Dell’insediamento Del Neolitico Antico Di Lugo Di Grezzana (Verona). In *Preistoria E Protostoria Del Veneto* Vol. 1 605–609 (Istituto Italiano di Preistoria e Protostoria, 2015).

[137] Cazzella, A., Cremaschi, M., Moscoloni, M. & Sala, B. Siti neolitici in località Razza Di Campegine (Reggio Emilia). *Preist. Alp.* **12**, 61–70 (1976).

[138] Berto, C., Bon, M. & Zampieri, S. I reperti faunistici provenienti Dal Sito Del Neolitico recente Di Botteghino (Parma). In *Atti Del 6° Convegno Nazionale Di Archeozoologia, Orecchiella, San Romano in Garfagnana—Lucca, 21–24 Maggio 2009* (eds De Grossi Mazzorin, J. et al.) 183–185 (Associazione Italiana di Archeozoologia, 2012).

[139] Depellegrin, V. La fauna del Neolitico recente, tardo e dell’Età del Rame di Isera—La Torretta (TN) (University of Trento, 2013).

[140] Riedel, A. The neolithic animal bones deposit of Cornuda (Treviso) Ferrara: Università di Ferrara. in *Annali dell’Università di Ferrara* vol. 1/6 71–90 (Università di Ferrara, Ferrara, 1988).

[141] Berto, C. La microfauna. In *Il Villaggio Neolitico Di Lugo Di Romagna—Fornace Gattelli. Strutture, Ambiente, Culture* (eds Steffè, G. & Degasperi, N.) 361–363 (Istituto Italiano di Preistoria e Protostoria, 2019).

[142] Catalani, P. Rivarolo mantovano: La fauna. *Preist. Alp.* **20**, 255–260 (1984).

[143] De Grossi Mazzorin, J. L’analisi archeozoologica Di Alcuni Siti Della cultura neolitica dei Vasi a Bocca quadrata Del Parmense. *Riv Stud. Liguri* 87–94 (2014).

[144] Agrostelli, M., Fontana, A. & Tecchiati, U. Castelnuovo Di Teolo (Padova), scavi 2011. I Dati archeobotanici e faunistici. In *Preistoria E Protostoria Del Veneto* Vol. 1 159–168 (Istituto Italiano di Preistoria e Protostoria, 2015).

[145] Girod, A. I molluschi terrestri e Di acqua Dolce Del Sito Della cultura dei Vasi a Bocca quadrata Di Riva Del Garda via Brione, proprietà Dutto. In *Vasi a Bocca Quadrata. Evoluzione Delle conoscenze Nuovi approcci Interpretativi*. (ed. *Mottes E)* 279–289 (2021).

[146] Bona, F. La fauna del sito di Tosina. In *Contadini, allevatori ed artigiani a Tosina di Monzambano (MN) tra V e IV millennio a. C. Una comunità neolitica nei circuiti padani e veneti* (ed. Poggiani Keller, R.) 137–146 (Acherdo Edizioni, Calcinato, 2014).

[147] Bona, F. Earliest evidence of Mus musculus ssp. In Western Europe during the late neolithic (Tosina, Mantova, Northern Italy): new Insights on the house mice migratory waves. *Hystrix Ital. J. Mamm.* **31**, 111–116 (2020).

[148] Borrello, M. A. Le Conchiglie Nella preistoria e Nella Protostoria. *Preist. Alp.* **40**, 19–42 (2005).

[149] Bernabò Brea, M., Maffi, M., Mazzieri, P. & Salvadei, L. Testimonianze funerarie Della gente dei Vasi a Bocca quadrata in Emilia occidentale. Archeologia e antropologia. *Riv Sci. Preist.* **60**, 63–126 (2010).

[150] Bazzanella, M., Trentini, M. & Wierer, U. Pike-fishing in the Adige valley: a research between archaeology and ethnography. In *Proceedings of the 4th Italian Congress of Ethnoarchaeology (Rome, 17–19 May 2006)* (eds. Lugli, F., Stoppiello, A. A. & Biagetti, S.) 62–68 (Archeopress, Oxford, 2011).

[151] Wierer, U. et al. Living near the water. Environment, wetland economy and fishing techniques of the Mesolithic site Galgenbuhel/Dos De La Forca in the Adige Valley (South Tyrol, Italy). In *Au coeur des sites mésolithiques: entre processus taphonomiques et données archéologiques : actes de la table-ronde internationale de Besançon (Doubs, France), Hommages au professeur André Thévenin, 29–30 Octobre 2013* (eds. Cupillard, C., Griselin, S. & Séara, F.) vol. Environnement, sociétés et archéologie 24 241–257 (Presses universitaires de Franche-Comté., Besançon, 2018).

[152] Bertsch, K. *Früchte Und Samen. Ein Bestimmungsbuch Zur Pflanzenkunde Der Vorgeschichtlichen Zeit* (Enke, 1941).

[153] Nesbitt, M. *Identification Guide for Near Eastern Grass Seeds* (Routledge, 2009). 10.4324/9781315427096

[154] Bojnanský, V. & Fargašová, A. *Atlas of Seeds and Fruits of Central and East-European Flora: the Carpathian Mountains Region* (Springer Science & Business Media, 2007).

[155] Marston, J.M., D'Aploim Guedes, J. & Warriner, C.* Method and Theory in Paleoethnobotany* (University Press of Colorado, (2015).

[156] Fuller, D. Q. A start for archaeological Nutters: some edible nuts for archaeologists. (2007).

[157] Sabato, D. & Peña-Chocarro, L. *Maris Nostri Novus Atlas: Seeds and Fruits from the Mediterranean Basin* (Doce Calles, 2021).

[158] Madella, M., Powers-Jones, A. H. & Jones, M. K. A simple method of extraction of opal phytoliths from sediments using a non-toxic heavy liquid. *J. Archaeol. Sci.* **25**, 801–803 (1998).

[159] Lancelotti, C. Not all that burns is wood’. A social perspective on fuel exploitation and use during the indus urban period (2600–1900 BC). *PLoS One* **13**, e0192364 (2018). 29513672 10.1371/journal.pone.0192364 PMC5841642

[160] Albert, R. & Weiner, S. Study of phytoliths in prehistoric Ash layers from Kebara and Tabun caves using a quantitative approach: 2nd international meeting on phytolith research. *Phytoliths Appl. Earth Sci. Hum. Hist* 251–266 (2001).

[161] France, D. L. *Human and Nonhuman Bone Identification: A Color Atlas* (CRC, 2008). 10.1201/9781420062878

[162] Schmid, E. *Atlas of Animal Bones. For Prehistorians, Archaeologists and Quaternary Geologists* (Elsevier Publishing Company, 1972).

[163] von den Driesch, A. den. *A Guide to the Measurement of Animal Bones from Archaeological Sites, as Developed by the Institut Für Palaeoanatomie, Domestikationsforschung Und Geschichte Der Tiermedizin of the University of Munich* (Peabody Museum of Archaeology and Ethnology, Harvard University, Cambridge, 1976).

[164] Wilkens, B. *Archeozoologia. Manuale Per Lo Studio Dei Resti Faunistici Dell’area Mediterranea* (Edes, 2012).

[165] Lazzari, G. *Conchigliando, Atlante Delle Conchiglie Della Costa Romagnola—Shell Atlas of Romagna’s Coast (Ravenna, Italy)* (Tipolitografia Scaletta, 2018).

[166] Libois, R. & Hallet, C. Eléments pour l’identification des restes crâniens des Poissons dulçaquicoles de Belgique et du Nord de La France. 2. Cypriniformes. *Fiches Ostéologie Anim. Pour Archéologie Sér Poissons* **4**, (1988).

[167] Radu, V. *Atlas for the Identification of Bony Fish Bones from Archaeological Sites* (Contrast, 2005).

[168] Davis, I., Sykes, N., Hochmuth, M. & Outram, A. K. & Roffet-Salque, M. A photographic atlas for European freshwater and migratory fish remains and key considerations for their analysis. *Int J. Osteoarchaeol* **34**, (2024).

[169] Bruno, S. & Maugeri, S. *Pesci D’acqua Dolce* (Giorgio Mondadori, 1992).

[170] Cossignani, T. & Cossignani, V. *Atlante delle conchiglie terrestri e dulciacquicole italiane*Ancona,. (1995).

[171] Morales-Muñiz, A. & Rosenlund, K. *Fish Bone Measurements. An Attempt To Standardize the Measurement of Fish Bones from Archaeological Sites* (Steenstrupia., 1979).

[172] Frezza, A. M. *Studio Morfologico E Morfometrico anatomo-scheletrico Di Pagellus Erythrinus Ed Esox Lucius Attuali Per L’interpretazione Archeozoologica Di Resti Ittiologici* (Department of Comparative and Evolutionary Biology, University of Naples Federico II, 1997).

[173] Jelu, I., Wouters, W. & Van Neer, W. The use of vertebral measurements for body length and weight reconstruction of Pike (Esox lucius) from archaeological sites. *Archaeol. Anthropol. Sci.* **13**, 72 (2021).

[174] Pessina, A., Fiappo, G. C. & Rottoli, M. Un Sito Neolitico a Pavia Di Udine. Nuovi Dati sull’inizio dell’agricoltura in Friuli. *Gortania* **25**, 73–94 (2003).

[175] Pessina, A., Fiappo, G. C. & Rottoli, M. Il Sito Neolitico Di Pavia Di Udine. Una sintesi Delle ricerche. *Gortania* **41**, 61–91 (2019).

[176] Pessina, A., Fontana, A., Rottoli, M., Occhini, E. & Salvador, S. Il Neolitico Della Bassa Pianura Friulana. Aspetti culturali, geoarcheologici e paleobotanici. In *Preistoria E Protostoria del. Caput Adriae* 135–146 (2018).

[177] Rottoli, M. & Regola, E. L’agricoltura in Italia settentrionale nel V millennio a.C.: nuovi dati dal sito di via Guidorossi a Parma. In *Il pieno sviluppo del Neolitico in Italia* (eds. Bernabò Brea, M., Maggi, R. & Manfredini, A.) 55–61 (2014).

[178] Rottoli, M. & Cottini, M. Vegetazione e agricoltura al tempo dei vasi a bocca quadrata. Nuove indicazioni dai siti di Vela II, III, VIII e Riva del Garda via Brione (Trento). In *Vasi a Bocca Quadrata. Evoluzione delle conoscenze e nuovi approcci interpretativi* (ed. Mottes, E.) 227–248 (Provincia Autonoma di Trento, Trento, 2021).

[179] Castiglioni, E. I carboni Di Legna e i resti carpologici dall’insediamento Neolitico Di Maserà Di Padova, via Bolzani. In *Vasi a Bocca Quadrata. Evoluzione Delle conoscenze Nuovi approcci Interpretativi*. (ed. Mottes E) 249–255 (2021).

[180] Castelletti, L., Castiglioni, E., Leoni, L. & Rottoli, M. Resti botanici Dai contesti Del Neolitico medio-recente, appendice 3. *Bull. Paletnol. Ital.* **89**, 191–200 (1998).

[181] Pini, R., Castellano, L., Perego, R., Ravazzi, C. & Rizzi, A. Un insediamento perilacustre del Tardo Neolitico—Età del Rame a Fimon Le Fratte. Successione sedimentaria, stratigrafia pollinica e macroresti vegetali. In *Nuove ricerche nelle Valli di Fimon. L’insediamento del tardo Neolitico de Le Fratte di Arcugnano* (ed. Bianchin Citton, E.) 169–194 (Editrice Veneta, Vicenza, 2016).

[182] Zanetti, A. L. & Tecchiati, U. I resti faunistici provenienti Da Una fossa Della cultura Di Fiorano (Neolitico antico) scavata in località S. Andrea Di Cologna Veneta (Verona). Dati preliminari. *Atti Dell8° Convegno Naz. Archeozool.* **1**, 55–62 (2019).

[183] Bon, M. La fauna neolitica Della Grotta Degli Zingari nel Carso Triestino. *Atti Della Soc. Preistoria E Protostoria Della Reg. Friuli Venezia Giulia*. **10**, 127–135 (1996).

[184] Boschin, F. & Riedel, A. The late mesolithic and neolithic fauna of the Edera cave (Aurisina, Trieste Karst): a preliminary report. In *Atti Della Società Per La Preistoria E La Protostoria Del Friuli-Venezia Giulia* Vol. 8 73–90 (Edizioni Svevo, 2000).

[185] Boschin, F. Holocene macromammal remains from Grotta dell’Edera/Stenašca, Trieste karst (excavations in 1990–2001). *Arheol Vestn*. **71**, 321–357 (2020).

[186] Girod, A. La malacofauna. In *Il Villaggio Neolitico Di Lugo Di Romagna—Fornace Gattelli. Strutture, Ambiente, Culture* (eds Steffè, G. & Degasperi, N.) 339–350 (Istituto Italiano di Preistoria e Protostoria, 2019).

[187] Boscato, P. & Crezzini, J. Allevamento e caccia: i resti Di macrofauna. In *Il Villaggio Neolitico Di Lugo Di Romagna—Fornace Gattelli. Strutture, Ambiente, Culture* (eds Steffè, G. & Degasperi, N.) 365–378 (Istituto Italiano di Preistoria e Protostoria, 2019).

[188] Gala, M. & Tagliacozzo, A. I resti Ossei Di avifauna. In *Il Villaggio Neolitico Di Lugo Di Romagna—Fornace Gattelli. Strutture, Ambiente, Culture* (eds Steffè, G. & Degasperi, N.) 357–360 (Istituto Italiano di Preistoria e Protostoria, 2019).

[189] Fontana, A., Marrazzo, D. & Spinetti, A. Studio Dello Sfruttamento Delle risorse animali tramite Le analisi archeozoologiche Delle faune Dai Siti Di Riva Del Garda—via Brione e La Vela Di Trento. In *Vasi a Bocca Quadrata. Evoluzione Delle conoscenze Nuovi approcci Interpretativi*. (ed. Mottes E) 65–86 (2021).

[190] Barker, G. Neolithic subsistence in the central Po plain. In *La Stazione Di Casatico Di Marcaria (Mantova) Nel Quadro Paleoambientale Ed Archeologico dell’Olocene Antico Della Val Padana Centrale* (eds Biagi, P. et al.) 45–72 (Istituto universitario, 1983).

[191] Tecchiati, U. I resti faunistici del Neolitico recente (III fase VBQ) di Maserà e Monselice (Padova). in *Dinamiche insediative nel territorio dei Colli Euganei dal Paleolitico al Medioevo. Atti del convegno di studi, Este—27 Novembre 2009, Monselice—28 Novembre 2009* (eds. Bianchin Citton, E., Rossi, S. & Zanovello, P.) 107–120 (Edizioni La Torre, Monselice, 2015).

[192] Riedel, A. La fauna neolitica Di Olmo Di Nogara (VR). *Quad. Archeol. Venetol.* **11**, 56–63 (1995).

[193] Petrucci, G. La fauna. in *Bannia-Palazzine di Sopra. Una comunità preistorica del V millennio a.C.* (ed. Visentini, P.) 146–170 (2005).

[194] Ottomano, C. Modalità Di Uso Del Suolo ed evoluzione paleoambientale Del sito. Le evidenze micromorfologiche. In *Bannia—Palazzine Di Sopra. Una comunità Preistorica Del V Millennio a.C* 31–41 (Comune di Pordenone Editore, 2005).

[195] Petrucci, G. & De March, M. & Thun Hohenstein, U. I resti di fauna dai pozzetti neolitici di Gazzo Veronese—Loc. Scolo Gelmina. Risultati preliminari dell’analisi tafonomica. In *Atti del 7° Convegno Nazionale di Archeozoologia 22–24 novembre 2012* (eds. Thun Hohenstein, U., Cangemi, M., Fiore, I. & De Grossi Mazzorin, J.) 39–46 (Ferrara, 2015).

