# Supplementary material for: Reassessing neolithic subsistence in Northern Italy through a critical review and new evidence from Molino Casarotto
Source: Sci Rep. 2025 Nov 28;15:44494. doi: 10.1038/s41598-025-28005-6 (PMC12738701; doi:10.1038/s41598-025-28005-6)
Supplement: Supplementary file 1 — Supplementary Material 1 [file 41598_2025_28005_MOESM1_ESM.pdf]

Supplementary Information for

Reassessing Neolithic subsistence in northern Italy through a critical review and new evidence from Molino Casarotto

Francesco Breglia[1*], Vito Giuseppe Prillo[2], Marta Dal Corso[1], Maria Sofia Manfrin[3], Silvia D'Aquino[2], Federico Polisca[1], Luigi Germinario[1], Giorgio Piazzalunga[2], Paola Salzani[4], Cristiano Nicosia[1]

[1] Dipartimento di Geoscienze, Università di Padova, Padova, Italy - 35131

[2] Dipartimento di Beni Culturali, Università di Padova, Padova, Italy - 35139

[3] Dipartimento di Beni Culturali e Ambientali, Laboratorio di Preistoria, Protostoria ed Ecologia Preistorica, Università degli Studi di Milano, Milano, Italy - 20141

[4] Soprintendenza Archeologia, Belle Arti e Paesaggio per le province di Verona. Rovigo e Vicenza, Verona, Italy - 37121

*corresponding author

Email: francesco.breglia@unipd.it

This PDF file includes:

Supporting text on methods for phytolith analysis
Figures S1 to S2
Tables S1 to S9
SI References

Supporting Information Text

Supplemental Method - Phytolith analysis

For the extraction of siliceous microfossils from the main stratigraphic units excavated at Molino Casarotto (Arcugnano, Vicenza, Italy) the laboratory protocol for sample processing followed Madella et al.[1]. The protocol includes:

- digestion of carbonates with HCl 5% until the end of reaction and 3x washing with H_2O and centrifuging;
- dissolution of clays with $NaPO_3$ overnight and multiple washing with H_2O and centrifuging until surnatant is clear of clays;
- digestion of organics with H_2O_2 on the hot plate for 8 hours and 3x washing with H_2O and centrifuging;
- sieving on a 250 mμ mesh to remove macro-remains and large sediment particles;
- heavy liquid separation with SPT 2.35 g/ml: 15 min centrifuging of sample and heavy liquid mixed with vortex, absorption of floating particles with a pipette and transferring into a new centrifuge tube (repeated twice), multiple washing with H_2O of the samples for the removal of heavy liquid residues;
- transferring of the sample with ethanol in a glass vial and drying in oven at 60 degrees. Storing as dry powder in the glass vial.

For the calculation of acid insoluble fraction (AIF), the procedure follows Lancelotti[2], adapted from Albert and Weiner[3]. Phytoliths nomenclature respects ICPN 2.0[4]. Seventeen sediment samples have been processed that came from eleven different stratigraphic units as in Table S3 and Table S4.

Supplementary figures and tables

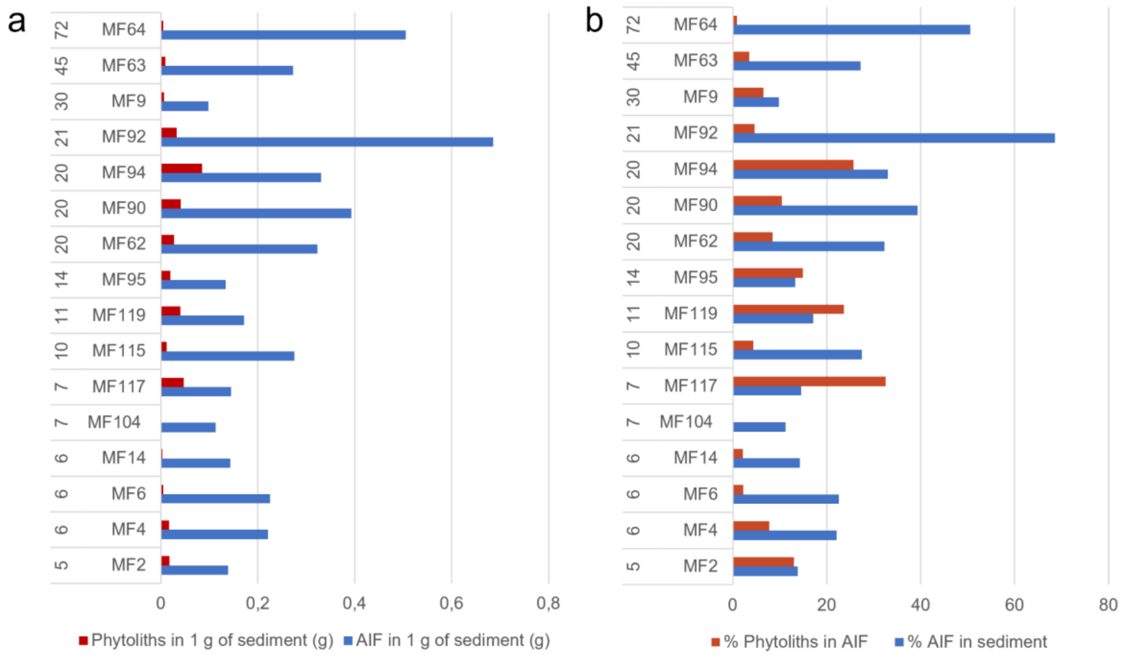

Figure S1. a) Weight of phytoliths and AIF in sediment; b) percentage of phytoliths and AIF in sediment. Sample labels indicate, from right to left, the number of the stratigraphic units and the microfossils samples (MF).

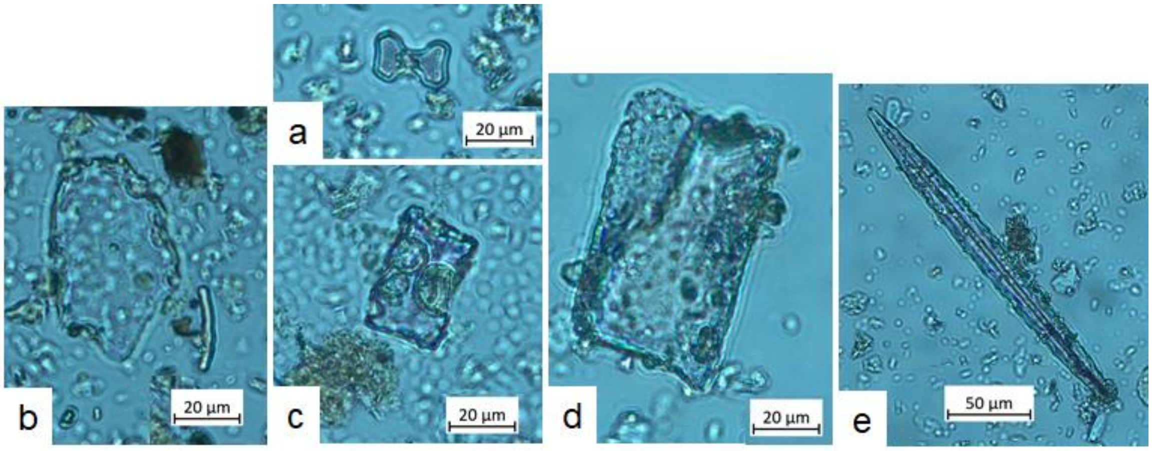

Figure S2. a) BILOBATE from MF90; b) BULLIFORM FLABELLATE from MF2; c) BLOCKY from MF6; d) BLOCKY from MF64; e) sponge spicule from MF64.

Table S1. Radiocarbon determinations available for each site considered in this study. Pre-AMS dates were excluded from the definition of the chronological range (spanning from the earliest to the latest available date). Where possible, only determinations referring to the archaeological layers analyzed in this study were used. All radiocarbon dates were recalibrated in OxCal to obtain calibrated age ranges. Archaeological phases and references are provided for each site, including those lacking radiocarbon determinations.

Site	n. 14C dates available	absolute dating uncal BP	absolute dating cal BCE (2s) recalibrated (p. 95,4%)	Chrono-cultural facies
Lugo di Romagna[5,6]	9	6585±87 (R-2748)/ 6131±50 (LTL-13440A)	5665-5369/5215-4935	Fiorano Culture
Grotta dell'Edera - layer 2a[7]	11 (5 pre-AMS, not considered)	6620±60 (GrA-19912)/ 6410±50 (GrN-26795)	5641-5475/ 5477-5231	Vlaska Group
Fagnigola[8]	7	6570±75 (R-2550)/ 6050±90 (R-1544a)	5631-5375/5212-4728	Fagnigola Group
Sammardenchia [8,9]	21	6570±74 (R-2547)/ 5684±58 (R-2735)	5631-5375/4681-4367	Sammardenchia Group
Valer[8]	3	6557± 71 (Bln-4436)/ 6504±59 (Bln-4431)	5626-5374/5613-5333	Sammardenchia Group
Isera - La Torretta[10]	5	6530±75 (R-723)/ 5440±55 (ETH-12494)	5622-5336/4442-4058	SMP III
Lugo di Grezzana[11]	13	6524±76 (R-2745)/ 5946±24 (DSH-462)	5621-5331/4901-4726	Fiorano Culture-SMP I
Piancada[8]	9	6410±70 (OZB-948)/ 5869±71 (OZB-654)	5481-5217/4931-4545	Sammardenchia Group
Cologna Veneta [12,13]	0	x	x	Fiorano Culture
Spilamberto - via Macchioni [14]	0	x	x	Fiorano Culture
Parma, Benefizio[15,16]	2	6394±45/5407±45 (LTL-5036a)	5475-5229/4349-4060	SMP I
Bazzarola – phase 1[17]	8	6390±40 (BAZZ-14)/ 6170±40 (BAZZ-17)	5474-5229/5217-4997	Fiorano Culture
Casa Gazza[18,19]	2	6130±160 (I-13798)/ 5830±210 (I-13799)	5471-4702/5289-4264	Vhò Group
Fimon - Molino Casarotto[18,20]	32	5800±50 (R-757a)/ 5140±50 (R-750a)	4789-4537/4047-3797	SMP I
Casalecchio di Reno[21,22]	2	6312± 50 (LTL-2436)/ 6189± 50 (LTL-2435a)	5469-5083/5299-5000	Fiorano Culture
Isorella[23]	4 (1 pre-AMS, not considered)	6290±34 (OxA-35333)/ 5850±34 (OxA-23072)	5359-5208/4797-4609	Vhò Group
Rivaltella - Cà Romensini[24]	6 (1 pre-AMS, not considered)	6055±65 (CRG-1056)/ 5794±50 (LTL-4559A)	5208-4793/4785-4506	Initial SMP

Vhò di Piadena[23]	8 (2 pre-AMS, not considered)	6190±25 (GrM-15259)/ 5895±35 (GrM-12690)	5216-5045/4846-4688	Vhò Group
Ostiano Dugali Alti[23]	3 (1 pre-AMS, not considered)	6184±18(GrM-12416)	5213-5052	Vhò Group
Pavia di Udine[25]	10	6127±40 (LTL4247A)/ 5560±45 (LTL4254A)	5209-4951/4493-4336	Sammardenchia Group
Isolino Virginia[26,27]	7	6068±29 (ETH-96188)/ 5879±29 (ETH-96186)	5200-4849/4835-4687	Isolino Group
Grotta degli Zingari[28]	0	x	x	Vlaška Group
Riparo Gaban[29]	4	6030±45 (Bln-1777)/ 5650±150 (Gif-3766)	5041-4797/4888-4077	Gaban Culture
La Vela - Sito I-II-III[30]	3	6038±31 (KIA-30554)/ 5914±44 (UtC-10550)	5026-4841/4930-4691	Gaban Culture
Bannia - Palazzine di Sopra[31]	10	5880±110 (GrN25736)/ 5240±110 (GrN25996)	5026-4460/4331-3801	SMP III
Razza di Campegine[32,33]	3	5940±40 (GX-29087)/ 5530±150 (Birm-829)	4934-4718/4710-4000	Fiorano Culture and SMP II-III
Rocca di Rivoli[34,35]	4	5670±130 (Birm-104)/ 4975±40 (Hd-23109)	4834-4256/3936-3648	SMP II-III
Forlì - Via Navicella[14,36]	2	5850±30 (UGAMS-4256)/5410±30 (UGAMS-4255)	4795-4612/4342-4171	Ripoli/Diana
Ponte Ghiara[37]	1	5842±40 (LTL-4572a)	4795-4554	SMP I
Riva del Garda - via Brione[38,39]	1	5832±28 (DSH8312_C)	4787-4608	SMP II
Grotta del Mitreo - trench 5[40,41]	1	5770±50 (R-904a)	4773-4462	Danilo Culture
Vicofertile[15,42]	2	5740±45(LTL-4571a)/ 5357±50 (LTL-4569a)	4704-4460/4331-4051	SMP II
La Vela VIII[39]	4	5731±35 (DSH8300)/ 5458±28 (KIA30557)	4684-4461/4355-4251	SMP II
Gaione, Parco del Cinghio[15,22]	1	5656±30 (Hd-25829)	4550-4369	SMP II
Maserà di Padova [43–45]	1	5630±33 (Hd-22563)	4539-4363	Initial SMP III
Parma - Via Guidorossi[46]	3	5544±60 (LTL-5035b)/ 5488±45 (LTL-4570a)	4532-4259/4446-4250	SMP II
Casatico di Marcaria[47]	1	5500±40 (GrN-27947)	4446-4258	SMP II

Botteghino[48]	1	5456±25 (Hd-25299)	4352-4253	Chassey Culture
Isolino Virginia[27]	4	5384±41 (ETH-96193)/ 5017±28 (ETH-96190)	4338-4057/3946–3662	SMP/Chssey Culture
Monselice[49]	0	x	x	Initial SMP III
Rivarolo Mantovano[50]	1	5380±40 (GrN-27949)	4336-4056	SMP III
Castelnuovo di Teolo[51,52]	4	5360±40 (LTD 13667)/ 5228±45 (LTD 13666)	4330-4053/4229-3959	SMP III/Chssey Culture
Fimon-Le Fratte[53]	4	5333±45 (UBA-18366)/ 4790±31 (LTL 14338A)	4326-4045/3640-3523	SMP III/Chassey-Lagozza Culture
Tosina di Monzambano[54]	3 (1 excluded)	5199±45 (LTL 14338A)/ 4974±45 (LTL 14339A)	4227-3819/3941-3646	SMP/Chssey Culture
Spilamberto - Sito VIII[55]	1	4995±100 (I-11817)	4036-3538	Chassey-Lagozza Culture
Grotta dell'Edera - layer 2[7,56]	5 (3 pre-AMS)	5080±40 (GrN-26797)/ 4960±90 (GrN-26796)	3969-3781/3965-3534	Danilo-Hvar Culture
Palù di Livenza - sector 3[57]	3	5021±40 (LTL16158A)/ 4952±40 (LTL16159A)	3949-3661/3900-3643	Chassey-Lagozza Culture
Bazzarola – phase 2[55]	0	x	x	SMP III
Gazzo Veronese - Scolo Gelmina[58]	1	4975± 40 (Hd-23109)	3936-3648	SMP III
Belforte di Gazzuolo[59]	0	x	x	SMP III
Cornuda[60]	0	x	x	SMP III/Chassey-Lagozza Culture
Levata di Curtatone[55]	0	x	x	SMP III/Chassey-Lagozza Culture
Olmo di Nogara[61]	0	x	x	SMP III/Chassey-Lagozza Culture

Table S2. Comprehensive list of sediment samples collected and floated, indicating the stratigraphic unit, the square and sub-square of origin.

Site	Mesh size for flotation / sieve mesh size for residue	Sample n.	SU	Square	Sub-square	Volume (l)	Analysed
Molino Casarotto	0.3 mm/1 mm	MR1	-			4	NO
Molino Casarotto	0.3 mm/1 mm	MR2	4 base	D6		0.5	NO
Molino Casarotto	0.3 mm/1 mm	MR4				1	NO
Molino Casarotto	0.3 mm/1 mm	MR5				0.7	NO
Molino Casarotto	0.3 mm/1 mm	MR7				1.25	NO
Molino Casarotto	0.3 mm/1 mm	MR8	4 base	C5	SW	2.75	NO
Molino Casarotto	0.3 mm/1 mm	MR9	4 base	D4/D5		1.6	NO
Molino Casarotto	0.3 mm/1 mm	MR10	4 base	E4/E5		2.15	NO
Molino Casarotto	0.3 mm/1 mm	MR11	4 base	D5		1.5	NO
Molino Casarotto	0.3 mm/1 mm	MR12	4 base	D5		0.95	NO
Molino Casarotto	0.3 mm/1 mm	MR13	4	E6		1.1	NO
Molino Casarotto	0.3 mm/1 mm	MR14	4 base	D7		0.75	NO
Molino Casarotto	0.3 mm/1 mm	MR15	11 base	F5		0.4	NO
Molino Casarotto	0.3 mm/1 mm	MR16	11 base	G4		1	YES
Molino Casarotto	0.3 mm/1 mm	MR17	7	C4	NW	2	YES
Molino Casarotto	0.3 mm/1 mm	MR18	7	B4	NE	2	YES
Molino Casarotto	0.3 mm/1 mm	MR19	7	E3		2	NO
Molino Casarotto	0.3 mm/1 mm	MR20	7	D3	NW	2	YES
Molino Casarotto	0.3 mm/1 mm	MR21	7	C3	NW	2	YES
Molino Casarotto	0.3 mm/1 mm	MR22	7	B3	NE	2	NO
Molino Casarotto	0.3 mm/1 mm	MR23	7	B2	NE	2	NO
Molino Casarotto	0.3 mm/1 mm	MR24	7	E5		0.25	YES
Molino Casarotto	0.3 mm/1 mm	MR25	7	F2	NW	2	YES
Molino Casarotto	0.3 mm/1 mm	MR26	7	E2	NE	2	YES
Molino Casarotto	0.3 mm/1 mm	MR27	7	H2	NW	2	NO
Molino Casarotto	0.3 mm/1 mm	MR28	7	G3	NW	2	YES
Molino Casarotto	0.3 mm/1 mm	MR29	7	F3	NE	2	YES
Molino Casarotto	0.3 mm/1 mm	MR30	7	F4	NW	2	YES
Molino Casarotto	0.3 mm/1 mm	MR31	9	E5	NW	2	YES
Molino Casarotto	0.3 mm/1 mm	MR32	7	G2		2	YES
Molino Casarotto	0.3 mm/1 mm	MR33	9	D4	SW	1	YES
Molino Casarotto	0.3 mm/1 mm	MR34	9	F5	NW	2	YES
Molino Casarotto	0.3 mm/1 mm	MR35	7	G3	NW	1	YES
Molino Casarotto	0.3 mm/1 mm	MR36	9	E4	SW	2	YES
Molino Casarotto	0.3 mm/1 mm	MR37	7	G4	SW-SE	2	YES
Molino Casarotto	0.3 mm/1 mm	MR38	7	E6	NE	2	YES
Molino Casarotto	0.3 mm/1 mm	MR39	7	F6	NW	2	YES
Molino Casarotto	0.3 mm/1 mm	MR40	7	G6		2	YES

Molino Casarotto	0.3 mm/1 mm	MR41	7	C4	SW	2	YES
Molino Casarotto	0.3 mm/1 mm	MR42	7	G5	NW	2	YES
Molino Casarotto	0.3 mm/1 mm	MR43	7	C5	NW	2	YES
Molino Casarotto	0.3 mm/1 mm	MR44	7	E8		0.5	YES
Molino Casarotto	0.3 mm/1 mm	MR45	7	B5	SE	2	YES
Molino Casarotto	0.3 mm/1 mm	MR46	7	D4	SW	2	YES
Molino Casarotto	0.3 mm/1 mm	MR47	7	B6	NE	2	YES
Molino Casarotto	0.3 mm/1 mm	MR48	7	G3	SE	1	NO
Molino Casarotto	0.3 mm/1 mm	MR49	7	H5	NW	2	YES
Molino Casarotto	0.3 mm/1 mm	MR50	7	H6	NW-SW	2	YES
Molino Casarotto	0.3 mm/1 mm	MR51	7	B7	NE	2	NO
Molino Casarotto	0.3 mm/1 mm	MR52	7	H7	NW-SW	2	YES
Molino Casarotto	0.3 mm/1 mm	MR53	5	D4	NE	0.35	YES
Molino Casarotto	0.3 mm/1 mm	MR54	20	C5	NE	2	YES
Molino Casarotto	0.3 mm/1 mm	MR55	20	C5	SE	2	YES
Molino Casarotto	0.3 mm/1 mm	MR56	20	C5	NW	1	NO
Molino Casarotto	0.3 mm/1 mm	MR57	20	C5	SW	2	YES
Molino Casarotto	0.3 mm/1 mm	MR58	5	D3	SE	1	YES
Molino Casarotto	0.3 mm/1 mm	MR59	5	D3	SW	1	YES
Molino Casarotto	0.3 mm/1 mm	MR60	20	C6	NE	2	YES
Molino Casarotto	0.3 mm/1 mm	MR61	25	G2	NW	2	YES
Molino Casarotto	0.3 mm/1 mm	MR62	20	C6	CENTRE-EAST	2	YES
Molino Casarotto	0.3 mm/1 mm	MR63	20	C6	NW	1	YES
Molino Casarotto	0.3 mm/1 mm	MR64	20	C6	CENTRE-WEST	2	YES
Molino Casarotto	0.3 mm/1 mm	MR65	42	C5	NE	0.1	YES
Molino Casarotto	0.3 mm/1 mm	MR66	6	C5	NE	0.2	YES
Molino Casarotto	0.3 mm/1 mm	MR67	6	C4	SE	3.5	YES
Molino Casarotto	0.3 mm/1 mm	MR68	6	D4	NW	4	YES
Molino Casarotto	0.3 mm/1 mm	MR69	25	G4	SW-SE	2	NO
Molino Casarotto	0.3 mm/1 mm	MR70	6	D3	SW	4.75	YES
Molino Casarotto	0.3 mm/1 mm	MR71	25	G5	NW	2	YES
Molino Casarotto	0.3 mm/1 mm	MR72	27	E7/E8		0.5	YES
Molino Casarotto	0.3 mm/1 mm	MR73	26	E7		0.5	NO
Molino Casarotto	0.3 mm/1 mm	MR74	19	B8		1.6	YES
Molino Casarotto	0.3 mm/1 mm	MR75	19	B7		3.5	YES
Molino Casarotto	0.3 mm/1 mm	MR76	20	D6	NE-NW	5.8	YES
Molino Casarotto	0.3 mm/1 mm	MR77	20	D5	SE-SW	3.5	YES
Molino Casarotto	0.3 mm/1 mm	MR78	20	E5	SW	5	YES
Molino Casarotto	0.3 mm/1 mm	MR79	20	E6	NW	3.5	YES
Molino Casarotto	0.3 mm/1 mm	MR80	22	D6		0.5	NO
Molino Casarotto	0.3 mm/1 mm	MR81	45	C4	SE	0.15	NO
Molino Casarotto	0.3 mm/1 mm	MR82	72	D6		0.2	YES
Molino Casarotto	0.3 mm/1 mm	MR83	60	D3	SE-SW	1	YES
Molino Casarotto	0.3 mm/1 mm	MR84	60	D4	NW	0.1	YES

Molino Casarotto	0.3 mm/1 mm	MR85	53	sez AA'			NO
Molino Casarotto	0.3 mm/1 mm	MR86	67	D6		0.5	YES
Molino Casarotto	0.3 mm/1 mm	MR87	46	C4		0.5	YES
Molino Casarotto	0.3 mm/1 mm	MR88	60	?	SW	0.5	NO
Molino Casarotto	0.3 mm/1 mm	MR89	21	C6		1.35	YES
Molino Casarotto	0.3 mm/1 mm	MR90	21	D6		1.25	YES
Molino Casarotto	0.3 mm/1 mm	MR91	21	D6		4.5	YES
Molino Casarotto	0.3 mm/1 mm	MR92	48	C4		0.6	YES
Molino Casarotto	0.3 mm/1 mm	MR93	66	D4	NW	2	YES
Molino Casarotto	0.3 mm/1 mm	MR94	73	C6		0.1	YES
Molino Casarotto	0.3 mm/1 mm	MR95	73	D6		0.3	YES
Molino Casarotto	0.3 mm/1 mm	MR96	25	D4	NW	2	YES
Molino Casarotto	0.3 mm/1 mm	MR97	25	D3	SW	3	YES
Molino Casarotto	0.3 mm/1 mm	MR98	14	C6		0.1	NO
Molino Casarotto	0.3 mm/1 mm	MR99	14	D6		0.3	NO
Molino Casarotto	0.3 mm/1 mm	MR100	25	D3	NW	1.5	NO
Molino Casarotto	0.3 mm/1 mm	MR101	20	D5	NW	1.5	YES
					**Tot. volume**	**166.15**	

Table S3. Archaeobotanical analysis results.

SU interpretation			Abandonement layers		Charcoal layers		Northern hearth						Reworked hearth residues				Pre-site peat	Other			
**SU**			**11 base**	**7**	**9**	**20**	**5**	**6**	**42**	**46**	**48**	**60**	**66**	**21**	**67**	**72**	**73**	**25**	**19**	**27**	**Sez. fac A-A1**	**TOT**
**Sample volume (l)**			1	48	7	32.3	2.35	12.45	0.4	0.5	0.6	1.1	2	7.1	0.5	0.2	0.4	11	5.1	0.5	0.5	133
**Categories**	**Plant part**	**Cons**																				
**Cereals**																						
Cerealia	grain	c					1															**1**
Hordeum vulgare	grain	c		1																		**1**
Triticum aestivum/durum	grain	c		1		1																**2**
Triticum dicoccum	grain	c			1		2															**3**
Triticum monococcum	grain	c				2																**2**
**Pulses**																						
Vicia sp.	seed	c			3			1														**4**
Lens culinaris	seed	c						1														**1**
Pisum sp.	seed	c				1																**1**
**Trees & shrubs**																						
Acer sp.	pericarp	c					1															**1**
cfr. *Anagyris foetida*	seed	c				1																**1**
Corylus avellana	pericarp	c		2			1															**3**
	seed	c		8		4	2					1									1	**16**
Cornus mas	seed	c						1				1										**2**
Euonymus latifolius	seed	c						3				3										**6**
Fagus sylvatica	pericarp	c		1																		**1**
	seed	c		1										2						1		**4**
Hedera helix	seed	c				1	4	3		1	3	2						1				**15**

SU interpretation			Abandonement layers			Charcoal layers		Northern hearth						Reworked hearth residues				Pre-site peat	Other			
**SU**			**11 base**	**7**	**9**	**20**	**5**	**6**	**42**	**46**	**48**	**60**	**66**	**21**	**67**	**72**	**73**	**25**	**19**	**27**	**Sez. fac A-A1**	**TOT**
**Sample volume (l)**			1	48	7	32.3	2.35	12.45	0.4	0.5	0.6	1.1	2	7.1	0.5	0.2	0.4	11	5.1	0.5	0.5	133
Ilex aquifolium	seed	c						1														**1**
Ligustrum vulgare	seed	c				1		1														**2**
Paliurus spina-christi	pericarp	c				1																**1**
Quercus sp.	pericarp	c		1				1														**2**
	seed	c		3		5						4		2					2			**16**
Rubus sp.	seed	c				1		2						3								**6**
Rubus idaeus	seed	c						1														**1**
Rubus saxatilis	seed	c				1																**1**
Sambucus nigra	seed	uc				1																**1**
Sambucus racemosa	seed	uc		1																		**1**
Tilia platyphyllos	seed	c		1																		**1**
Vitis vinifera subsp. *sylvestris*	seed	c						2														**2**
**Grassland. undergrowth and woodland margins herbs**																						
Agrimonia procera	seed	c												1								**1**
Ajuga chamaepitys	seed	uc				1													1			**2**
Brassica sp.	seed	c				1		2														**3**
Chenopodium opulifolium	seed	uc		22	2	6	1															**31**
Cruciata laevipes	mericarp	c				2																**2**
Erodium moschatum	seed	c					2															**2**
Galeopsis angustifolia	seed	uc				1																**1**
Galeopsis laudanum	seed	uc		1																		**1**

**SU interpretation**			**Abandonement layers**			**Charcoal layers**		**Northern hearth**						**Reworked hearth residues**				**Pre-site peat**	**Other**			
**SU**			**11 base**	**7**	**9**	**20**	**5**	**6**	**42**	**46**	**48**	**60**	**66**	**21**	**67**	**72**	**73**	**25**	**19**	**27**	**Sez. fac A-A1**	**TOT**
**Sample volume (l)**			1	48	7	32.3	2.35	12.45	0.4	0.5	0.6	1.1	2	7.1	0.5	0.2	0.4	11	5.1	0.5	0.5	133
Galium odoratum	seed	c				1																**1**
Melittis melissophyllum	seed	c					2	1														**3**
Moehringia trinervia	seed	nc				1												1				**2**
Myosotis sylvatica	seed	c				1																**1**
Ononis natrix	seed	uc		13																		**13**
Parietaria officinalis	seed	c				1																**1**
Persicaria lapathifolia	seed	uc		36		56												2				**94**
Plantago sp.	seed	uc				1																**1**
Polygonatum multiflorum	seed	c						1														**1**
Setaria pumila	floret	uc				1																**1**
Stachys alopecuros	seed	uc		1																		**1**
Urtica sp.	seed	c												1								**1**
Vincetoxicum sp.	seed	c				1																**1**
Viola sp.	seed	c				2																**2**
**Wet grassland. wetland and aquatic plants**																						
Bromus racemosus	seed	c		1																		**1**
Carex sp.	seed	c		5		6												2				**13**
		uc		11	6	2		2						1								**22**
	utricle	uc		1																		**1**
Cladium mariscus	seed	uc	1	136	27	34	2					2		1	1			6	1			**211**
Comarum palustre	seed	c				1																**1**
Eriophorum vaginatum	seed	uc		1																		**1**
Euphorbia helioscopia	seed	uc		3																		**3**

**SU interpretation**			**Abandonement layers**			**Charcoal layers**		**Northern hearth**						**Reworked hearth residues**				**Pre-site peat**	**Other**			
**SU**			**11 base**	**7**	**9**	**20**	**5**	**6**	**42**	**46**	**48**	**60**	**66**	**21**	**67**	**72**	**73**	**25**	**19**	**27**	**Sez. fac A-A1**	**TOT**
**Sample volume (l)**			1	48	7	32.3	2.35	12.45	0.4	0.5	0.6	1.1	2	7.1	0.5	0.2	0.4	11	5.1	0.5	0.5	133
Nymphaeacea	seed	uc	15	363	65	336	14	5				1	1	17		3		2	2		4	**828**
Najas major	seed	c				1																**1**
Najas marina	seed	c				1																**1**
Persicaria minor	seed	c		1																		**1**
		uc		1	5																	**6**
Potamogeton sp.	seed	c						1														**1**
Rumex conglomeratus	seed	uc				2																**2**
Rumex sanguineus	seed	uc				3																**3**
Schoenoplectus sp.	seed	c				1																**1**
Stachys palustris	seed	uc			1																	**1**
Trapa natans	seed	c		10		1		1						2								**14**
	pericarp	c	8	243	57	335	100	36	1			17	3	28	7	10	9	16	3	6	1	**880**
Trichophorum cespitosum	seed	uc				1																**1**
**Other herbaceous plants**																						
Armeria sp.	seed	uc				1																**1**
Asparagaceae	seed	c				1																**1**
Eryngium sp.	pericarp	uc				1																**1**
Fabaceae	seed	c			2	2																**4**
Lamium amplexiculae	seed	uc		12																		**12**
Muscari sp.	seed	c				1																**1**
Phlomis sp.	seed	uc						1														**1**
Poaceae	seed	c		1																		**1**
Ranunculus sp.	seed	uc					1															**1**

**SU interpretation**			**Abandonement layers**			**Charcoal layers**		**Northern hearth**						**Reworked hearth residues**				**Pre-site peat**	**Other**			
**SU**			**11 base**	**7**	**9**	**20**	**5**	**6**	**42**	**46**	**48**	**60**	**66**	**21**	**67**	**72**	**73**	**25**	**19**	**27**	**Sez. fac A-A1**	**TOT**
**Sample volume (l)**			1	48	7	32.3	2.35	12.45	0.4	0.5	0.6	1.1	2	7.1	0.5	0.2	0.4	11	5.1	0.5	0.5	133
Rumex sp.	seed	uc		6	1	1		2														**10**
**Other evidence**																						
AOV		c		1	3	4						4				3						**15**
n/d	other (leaves. spike. etc)	uc				2		1														**3**
	mesocarp	c				0										0						**0**
	pericarp	c		421	118	186	123									9		9				**866**
	seed	c		25	2	11	1	4										1				**44**
	n/d	c		158		453	121	93	2	4		66		95	21	41	15	21	6	19		**1115**
		uc		1															2			**3**
	**TOT**		**24**	**1494**	**293**	**1483**	**378**	**167**	**3**	**5**	**3**	**101**	**4**	**153**	**29**	**66**	**24**	**61**	**17**	**26**	**6**	**4337**

Table S4. Information about phytolith analysis: sampled contexts. AIF (Acid Insoluble Fraction) and phytoliths in sediment (see methods).

SU	Square	Unit description	Field sample n.	Lab sample n.	Dry sediment analysed (g)	AIF in 1 g of sediment (g)	Phytoliths in 1 g of sediment (g)	% AIF in sediment	% Phytoliths in AIF
5	D4	Charcoal-rich sediment surrounding the northern hearth SU 6 in squares C3. C4. D3. D4	MF2	PHY20	2.3007	0.1385	0.0180	13.852	12.990
6	D4	Ash from northern hearth in squares C3. C4. D3. D4	MF4	PHY21	2.6112	0.2210	0.0171	22.101	7.728
6	D4		MF6	PHY36	2.6873	0.2252	0.0049	22.521	2.198
6	D3		MF14	PHY22	2.5781	0.1426	0.0031	14.262	2.148
7	F2	Abandonment layer	MF104	PHY28	1.9489	0.1128	n.a.	11.278	n.a.
7	G7		MF117	PHY25	2.2924	0.1450	0.0471	14.496	32.501
10	G7	Substrate of carbonatic lake marl	MF115	PHY33	3.1883	0.2749	0.0120	27.488	4.382
11	G7	Clayey deposit above the anthropogenic units	MF119	PHY31	2.789	0.1713	0.0405	17.132	23.629
14	C6	Shell midden below SU 21	MF95	PHY32	3.046	0.1331	0.0198	13.313	14.846
20	D6	Extensive charcoal concentration S. SW and SE of SU 6 comparable to SU 5	MF62	PHY30	1.8104	0.3225	0.0273	32.247	8.479
20	D6		MF90	PHY23	2.7425	0.3929	0.0409	39.289	10.422
20	D5		MF94	PHY29	2.7549	0.3303	0.0847	33.028	25.651
21	D6	Small fired area in square D6. below SU 20	MF92	PHY26	2.5183	0.6855	0.0320	68.550	4.663
30	C4	Circular structure of calcinate stones above SU6 and below SU 5	MF9	PHY24	2.8628	0.0981	0.0064	9.809	6.553
45	C4	Silty-clayey layer below SU 6 on the SW	MF63	PHY35	2.6004	0.2724	0.0094	27.238	3.445
72	D6	Grey silt in square D6. below the ash layer of SU 22	MF64	PHY34	2.7757	0.5055	0.0044	50.553	0.869

Table S5. Identified taxa from the recent excavations categorized by anatomical and taxonomic groups, with corresponding quantities.

**Skeletal element**	*Cervus elaphus*	*Capreolus capreolus*	*Sus scrofa*	*Sus* sp.	*Bos taurus*	*Carnivora* (small carnivores)	*Micro-mammalia*	*Aves* sp.	*Emys* sp.	*Esox lucius*	*Tinca tinca*	*Cyprinidae* sp.
Palatine										1		
Dentary										15		
Pharyngeal bone											8	
Cleithrum										2		
Vertebrae										17		13
Cranium/ antler	5											
Maxilla												
Mandible		1										
Teeth	2	6			2	4	5					
Scapula												
Humerus			1	1								
Radius			1									
Carpal												
Metacarpal												
Phalanges												
Pelvis												
Femur												
Tibia								1				
Tarsal												
Metatarsal	1											
Metapodial						1						
Diaphyses							3					
Plastron									2			

Table S6. Size reconstruction of pike according to Frezza[62] for cranial remains and Jelu et al[63] for vertebrae with reference to their SU and square. Total Length (TL) is expressed in cm. while weight is expressed in gr.

Skeletal element	SU/Q	TL (Total Length)	Weight
Articular	4 Q. D5	68,5	2362,27
Dentary	5 Q. D4 NE	44,3	182,29
Dentary	20 Q. D6 NE/NW	31,8	253,86
Dentary	20 Q. C6 NE	34,6	415,65
Dentary	20 Q. D5	39,4	628,41
Dentary	20 Q. C6 NE	47	774,74
1st vertebra	20 Q. C5 NE	38,7	424,84
1st vertebra	20 Q. C5 NE	77,6	3474,73
1st vertebra	20 Q. C5 SE	79,9	3787,41
3rd vertebra	20 Q. C5 SW	47,4	768,13
5th vertebra	20 Q. D5	50,2	940,8
1st vertebra	21 Q. D6	83,4	4289,13

Table S7. Total Lenght (TL) and weight of pike expressed in their lowest and highest value. arithmetic mean and standard error.

Measurement	N.	Lowest	Arithmetic mean	Highest	Standard error
Total Lenght	12	31.8	53.6	83.4	18.62
Weight	12	182.29	1525.18	4289.13	1519.62

Table S8. Percentages of plant and animal remains in the assemblages from the Neolithic sites analyzed. Plant categories: Cereals – caryopses and chaff from certainly cultivated taxa: einkorn, emmer, other hulled and naked wheats, barley (grouped due to preservation and quantification biases, preventing detailed assessment of agricultural strategies); Pulses; Fresh fruits and berries – only edible species. Animal categories: Caprines – domestic sheep and goats; Suids – domestic pigs, wild boar, and unidentified suids; Cervids – red deer and roe deer; Large-sized mammals – large game other than cervids, less frequent at the sites, including bear, wolf, aurochs, and chamois; Small-sized mammals – foxes, mustelids, lagomorphs, and rodents; Reptiles/Amphibians – turtles, toads, or undetermined anurans; Freshwater fish – including catadromous migratory species such as European eel; Marine fish – including anadromous species such as sturgeons; Molluscs – terrestrial, freshwater, and marine molluscs, grouped due to limited taxonomic resolution, with presence noted regardless of species or environment. Macro-categories used in the statistical analyses are also indicated, combining several subcategories of remains for clarity in data presentation.

Macro-categories			Crops		Nuts		Fresh fruits		Domesticates				Wild game					Ichtyofauna		
Sites	Region	Chronology	Cereals %	Legumes %	Hazelnuts %	Acorns and beech nuts %	Fresh fruits and berries %	Water chestnut %	Caprines %	Cattle%	Suids %	Dog %	Cervids %	Large sized mammals %	Small sized mammals %	Avifauna %	Reptiles/ amphibians %	Freshwater fish %	Marine fish %	Mollusks %
Lugo di Romagna	Southern Po Plains	EN	72,50	0,12	10,41	6,85	9,91	0,21	2,21	4,43	1,72	0,37	2,03	0,00	2,34	0,98	0,00	5,10	27,80	53,01
Lugo di Grezzana	Garda-Venetian Prealps and Adige Valley	EN	47,34	0,00	3,84	44,98	3,84	0,00	17,91	65,67	10,45	0,00	4,48	0,00	1,49	0,00	0,00	0,00	0,00	0,00
La Vela - phase 1	Garda-Venetian Prealps and Adige Valley	EN	43,65	1,59	5,56	0,00	49,21	0,00	30,77	21,15	11,54	0,00	35,58	0,00	0,00	0,96	0,00	0,00	0,00	0,00
Piancada	Friulian Plains	EN	90,66	0,81	6,16	1,22	1,15	0,00	20,38	35,77	22,69	1,92	0,38	0,00	0,00	0,00	0,00	0,00	0,00	18,85
Bazzarola - phase 1	Southern Po Plains	EN	84,12	5,42	8,03	0,44	1,99	0,00	n/d	n/d	n/d	n/d	n/d	n/d	n/d	n/d	n/d	n/d	n/d	n/d
Spilamberto - Via Macchioni	Southern Po Plains	EN	24,18	74,51	0,00	0,00	1,31	0,00	n/d	n/d	n/d	n/d	n/d	n/d	n/d	n/d	n/d	n/d	n/d	n/d
Isolino Virginia - phase 1	Varese Prealps	EN	22,33	0,12	4,67	3,59	68,14	1,14	n/d	n/d	n/d	n/d	n/d	n/d	n/d	n/d	n/d	n/d	n/d	n/d

Sammardenchia	Friulian Plains	EN	43,07	0,96	55,25	0,09	0,62	0,00	n/d	n/d	n/d	n/d	n/d	n/d	n/d	n/d	n/d	n/d	n/d	n/d
Pavia di Udine	Friulian Plains	EN	65,35	0,39	33,85	0,00	0,41	0,00	n/d	n/d	n/d	n/d	n/d	n/d	n/d	n/d	n/d	n/d	n/d	n/d
Fagnigola	Friulian Plains	EN	2,03	0,00	97,82	0,00	0,15	0,00	n/d	n/d	n/d	n/d	n/d	n/d	n/d	n/d	n/d	n/d	n/d	n/d
Valer	Friulian Plains	EN	90,38	0,00	9,62	0,00	0,00	0,00	n/d	n/d	n/d	n/d	n/d	n/d	n/d	n/d	n/d	n/d	n/d	n/d
Riparo Gaban	Garda-Venetian Prealps and Adige Valley	EN	n/d	n/d	n/d	n/d	n/d	n/d	26,95	5,12	18,04	1,56	42,76	1,56	2,90	0,67	0,22	0,00	0,00	0,22
Cologna Veneta	Venetian Hills and Plains	EN	n/d	n/d	n/d	n/d	n/d	n/d	11,48	24,37	35,57	1,12	26,33	0,00	0,56	0,28	0,28	0,00	0,00	0,00
Grotta degli Zingari	Trieste Karst	EN	n/d	n/d	n/d	n/d	n/d	n/d	18,86	10,09	16,67	0,00	17,11	1,32	35,96	0,00	0,00	0,00	0,00	0,00
Grotta dell'Edera - Layer 2a	Trieste Karst	EN	n/d	n/d	n/d	n/d	n/d	n/d	97,89	1,11	0,44	0,11	0,33	0,00	0,11	0,00	0,00	0,00	0,00	0,00
Ostiano Dugali Alti	Central Po Plain	EN	n/d	n/d	n/d	n/d	n/d	n/d	9,13	13,46	32,21	2,88	38,46	0,00	0,96	2,40	0,48	0,00	0,00	0,00
Isorella	Central Po Plain	EN	n/d	n/d	n/d	n/d	n/d	n/d	14,74	14,21	29,47	4,21	19,47	0,00	10,53	0,53	6,84	0,00	0,00	0,00
Vhò di Piadena	Central Po Plain	EN	n/d	n/d	n/d	n/d	n/d	n/d	4,17	4,17	31,25	0,00	57,29	0,00	2,08	1,04	0,00	0,00	0,00	0,00
Casa Gazza	Southern Po Plains	EN	n/d	n/d	n/d	n/d	n/d	n/d	39,32	12,42	24,63	0,71	20,01	0,07	1,28	1,35	0,14	0,07	0,00	0,00
Casalecchio di Reno	Southern Po Plains	EN	n/d	n/d	n/d	n/d	n/d	n/d	38,83	45,21	12,23	0,00	3,72	0,00	0,00	0,00	0,00	0,00	0,00	0,00
Parma - Via Guidorossi	Southern Po Plains	MN	98,22	1,39	0,15	0,00	0,24	0,00	15,95	64,30	12,15	6,33	0,76	0,00	0,00	0,25	0,00	0,25	0,00	0,00
La Vela - phase 2	Garda-Venetian Prealps and Adige Valley	MN	85,67	0,71	3,41	0,15	10,05	0,00	51,94	20,56	20,83	0,13	6,14	0,00	0,40	0,00	0,00	0,00	0,00	0,00
Riva del Garda - via Brione	Garda-Venetian Prealps and Adige Valley	MN	82,48	1,34	12,53	0,07	3,59	0,00	28,38	36,70	30,15	0,41	0,00	0,00	4,23	0,14	0,00	0,00	0,00	0,00

Fimon - Molino Casarotto	Venetian Hills and Plains	MN	0,48	0,32	1,01	1,23	0,80	96,16	1,44	1,20	27,17	0,28	44,98	0,20	1,94	0,14	0,19	2,99	0,00	19,47
Maserà di Padova	Venetian Hills and Plains	MN	70,83	0,32	13,78	5,77	9,29	0,00	28,82	44,33	20,94	0,00	3,69	0,00	0,49	0,99	0,74	0,00	0,00	0,00
Ponte Ghiara	Southern Po Plains	MN	93,36	0,89	4,11	0,05	1,59	0,00	n/d	n/d	n/d	n/d	n/d	n/d	n/d	n/d	n/d	n/d	n/d	n/d
Rivaltella - Cà Romensini	Southern Po Plains	MN	93,40	0,90	3,00	1,20	1,50	0,00	n/d	n/d	n/d	n/d	n/d	n/d	n/d	n/d	n/d	n/d	n/d	n/d
Forlì - Via Navicella	Southern Po Plains	MN	59,19	0,00	0,00	37,03	3,78	0,00	n/d	n/d	n/d	n/d	n/d	n/d	n/d	n/d	n/d	n/d	n/d	n/d
Casatico di Marcaria	Central Po Plain	MN	n/d	n/d	n/d	n/d	n/d	n/d	16,82	45,88	34,71	0,35	2,12	0,00	0,12	0,00	0,00	0,00	0,00	0,00
Parma, Benefizio	Southern Po Plains	MN	n/d	n/d	n/d	n/d	n/d	n/d	1,25	85,00	11,25	0,00	0,00	2,50	0,00	0,00	0,00	0,00	0,00	0,00
Gaione, Parco del Cinghio	Southern Po Plains	MN	n/d	n/d	n/d	n/d	n/d	n/d	31,40	30,81	35,47	1,16	0,00	0,00	1,16	0,00	0,00	0,00	0,00	0,00
Vicofertile	Southern Po Plains	MN	n/d	n/d	n/d	n/d	n/d	n/d	22,22	72,73	5,05	0,00	0,00	0,00	0,00	0,00	0,00	0,00	0,00	0,00
Parma, via Guidorossi	Southern Po Plains	MN	n/d	n/d	n/d	n/d	n/d	n/d	15,95	64,30	12,15	6,33	0,76	0,00	0,00	0,25	0,00	0,25	0,00	0,00
Razza di Campegine	Southern Po Plains	MN	n/d	n/d	n/d	n/d	n/d	n/d	4,40	3,77	23,90	0,63	32,08	34,59	0,63	0,00	0,00	0,00	0,00	0,00
Rocca di Rivoli	Garda-Venetian Prealps and Adige Valley	MN	n/d	n/d	n/d	n/d	n/d	n/d	29,70	31,86	34,46	0,00	3,81	0,17	0,00	0,00	0,00	0,00	0,00	0,00
Grotta del Mitreo - trench 5	Trieste Karst	MN	n/d	n/d	n/d	n/d	n/d	n/d	26,47	1,47	0,37	0,37	2,21	0,00	1,10	0,74	0,00	0,00	0,00	67,28
Botteghino	Southern Po Plains	MN	n/d	n/d	n/d	n/d	n/d	n/d	47,25	12,39	13,30	0,00	27,06	0,00	0,00	0,00	0,00	0,00	0,00	0,00
Isera - La Torretta	Garda-Venetian Prealps and Adige Valley	MN	n/d	n/d	n/d	n/d	n/d	n/d	21,06	27,65	19,66	0,05	30,30	0,76	0,48	0,03	0,00	0,00	0,00	0,00
Tosina di Monzambano	Central Po Plain	LN	36,50	0,73	45,26	3,65	13,87	0,00	11,05	30,38	21,86	1,15	8,06	0,12	20,48	0,23	2,30	4,37	0,00	0,00

Castelnuovo di Teolo	Venetian Hills and Plains	LN	49,67	38,08	12,25	0,00	0,00	0,00	24,76	25,39	44,83	0,00	3,76	0,63	0,00	0,00	0,31	0,31	0,00	0,00
Bannia - Palazzine di Sopra	Friulian Plains	LN	28,04	4,51	58,99	0,41	8,06	0,00	58,05	16,28	25,67	0,00	0,00	0,00	0,00	0,00	0,00	0,00	0,00	0,00
Bazzarola - phase 2	Southern Po Plains	LN	80,60	0,44	2,05	11,66	5,25	0,00	n/d	n/d	n/d	n/d	n/d	n/d	n/d	n/d	n/d	n/d	n/d	n/d
Isolino Virginia	Varese Prealps	LN	72,12	0,63	1,98	4,37	19,46	1,44	n/d	n/d	n/d	n/d	n/d	n/d	n/d	n/d	n/d	n/d	n/d	n/d
Palù di Livenza - Sector 3	Friulian Plains	LN	21,24	0,33	0,00	0,00	78,43	0,00	n/d	n/d	n/d	n/d	n/d	n/d	n/d	n/d	n/d	n/d	n/d	n/d
Spilamberto - Sito I-VIII	Southern Po Plains	LN	68,56	2,06	0,52	0,00	28,87	0,00	n/d	n/d	n/d	n/d	n/d	n/d	n/d	n/d	n/d	n/d	n/d	n/d
Fimon - Le Fratte	Venetian Hills and Plains	LN	78,87	2,16	0,00	0,00	18,97	0,00	n/d	n/d	n/d	n/d	n/d	n/d	n/d	n/d	n/d	n/d	n/d	n/d
Levata di Curtatone	Central Po Plain	LN	97,00	1,12	0,94	0,02	0,91	0,01	n/d	n/d	n/d	n/d	n/d	n/d	n/d	n/d	n/d	n/d	n/d	n/d
Rivarolo Mantovano	Central Po Plain	LN	n/d	n/d	n/d	n/d	n/d	n/d	32,56	22,67	23,84	0,58	8,72	0,58	6,40	0,00	3,49	0,00	0,00	1,16
Grotta dell'Edera - Layer 2	Trieste Karst	LN	n/d	n/d	n/d	n/d	n/d	n/d	81,95	4,46	9,74	0,41	2,23	0,00	1,22	0,00	0,00	0,00	0,00	0,00
Belforte di Gazzuolo	Central Po Plain	LN	n/d	n/d	n/d	n/d	n/d	n/d	24,41	13,45	32,64	1,17	24,67	0,00	3,66	0,00	0,00	0,00	0,00	0,00
Monselice	Venetian Hills and Plains	LN	n/d	n/d	n/d	n/d	n/d	n/d	22,08	57,58	19,48	0,00	0,87	0,00	0,00	0,00	0,00	0,00	0,00	0,00
Olmo di Nogara	Central Po Plain	LN	n/d	n/d	n/d	n/d	n/d	n/d	14,08	36,62	36,62	7,04	1,41	0,00	1,41	0,00	2,82	0,00	0,00	0,00
Cornuda	Venetian Hills and Plains	LN	n/d	n/d	n/d	n/d	n/d	n/d	10,28	5,91	32,65	0,51	42,03	8,35	0,26	0,00	0,00	0,00	0,00	0,00

Table S9. Ubiquity measured for all identified taxa based on the samples and stratigraphic units. N_{total} (sample) = total number of analyzed samples; N_{taxon} (sample) = number of samples in which a specific taxon is present; U_{taxon} (sample) = ubiquity of the taxon measured based on the samples; N_{total} (SU) = total number of analyzed stratigraphic units; N_{taxon} (SU) = number of stratigraphic units in which a specific taxon is present; U_{taxon} (SU) = ubiquity of the taxon measured based on the stratigraphic units.

**Taxa**	N_{total} (samples)	N_{taxon} (samples)	**U_{taxon} (samples)**	N_{total} (SU)	N_{taxon} (SU)	**U_{taxon} (SU)**
**Cereals**						
Hordeum vulgare	71	1	0.014	18	1	0.056
Triticum aestivum/durum	71	2	0.028	18	2	0.111
Triticum dicoccum	71	3	0.042	18	2	0.111
Triticum monococcum	71	2	0.028	18	1	0.056
**Pulses**						
Vicia sp.	71	2	0.028	18	2	0.111
Lens culinaris	71	1	0.014	18	1	0.056
Pisum sp.	71	1	0.014	18	1	0.056
**Trees & shrubs**						
Acer sp.	71	1	0.014	18	1	0.056
cfr. *Anagyris foetida*	71	1	0.014	18	1	0.056
Corylus avellana	71	11	0.155	18	5	0.278
Cornus mas	71	2	0.028	18	2	0.111
Euonymus latifolius	71	4	0.056	18	3	0.167
Fagus sylvatica	71	4	0.056	18	3	0.167
Hedera helix	71	8	0.113	18	7	0.389
Ilex aquifolium	71	1	0.014	18	1	0.056
Ligustrum vulgare	71	2	0.028	18	2	0.111
Paliurus spina-christi	71	1	0.014	18	1	0.056
Quercus sp.	71	8	0.113	18	6	0.333
Rubus sp.	71	3	0.042	18	3	0.167
Rubus idaeus	71	1	0.014	18	1	0.056
Rubus saxatilis	71	1	0.014	18	1	0.056
Sambucus nigra	71	1	0.014	18	1	0.056
Sambucus racemosa	71	1	0.014	18	1	0.056
Tilia platyphyllos	71	1	0.014	18	1	0.056
Vitis vinifera subsp. *sylvestris*	71	1	0.014	18	1	0.056
**Grassland, undergrowth and woodland margins herbs**						
Agrimonia procera	71	1	0.014	18	1	0.056
Ajuga chamaepitys	71	2	0.028	18	2	0.111
Brassica sp.	71	2	0.028	18	2	0.111
Chenopodium opulifolium	71	14	0.197	18	5	0.278
Cruciata laevipes	71	2	0.028	18	1	0.056
Erodium moschatum	71	1	0.014	18	1	0.056
Galeopsis angustifolia	71	1	0.014	18	1	0.056

Galeopsis laudanum	71	1	0.014	18	1	0.056
Galium odoratum	71	1	0.014	18	1	0.056
Melittis melissophyllum	71	2	0.028	18	2	0.111
Moehringia trinervia	71	2	0.028	18	2	0.111
Myosotis sylvatica	71	1	0.014	18	1	0.056
Ononis natrix	71	3	0.042	18	1	0.056
Parietaria officinalis	71	1	0.014	18	1	0.056
Persicaria lapathifolia	71	22	0.310	18	3	0.167
Plantago sp.	71	1	0.014	18	1	0.056
Polygonatum multiflorum	71	1	0.014	18	1	0.056
Setaria pumila	71	1	0.014	18	1	0.056
Stachys alopecuros	71	1	0.014	18	1	0.056
Urtica sp.	71	1	0.014	18	1	0.056
Vincetoxicum sp.	71	1	0.014	18	1	0.056
Viola sp.	71	1	0.014	18	1	0.056
**Wet grassland, wetland and aquatic plants**						
Bromus racemosus	71	1	0.014	18	1	0.056
Carex sp.	71	23	0.324	18	7	0.389
Cladium mariscus	71	46	0.648	18	11	0.611
Comarum palustre	71	1	0.014	18	1	0.056
Eriophorum vaginatum	71	1	0.014	18	1	0.056
Euphorbia helioscopia	71	2	0.028	18	1	0.056
Nymphaeacea	71	53	0.746	18	14	0.778
Najas major	71	1	0.014	18	1	0.056
Najas marina	71	1	0.014	18	1	0.056
Persicaria minor	71	7	0.099	18	3	0.167
Potamogeton sp.	71	1	0.014	18	1	0.056
Rumex conglomeratus	71	1	0.014	18	1	0.056
Rumex sanguineus	71	1	0.014	18	1	0.056
Schoenoplectus sp.	71	1	0.014	18	1	0.056
Stachys palustris	71	1	0.014	18	1	0.056
Trapa natans	71	59	0.831	18	18	1.000
Trichophorum cespitosum	71	1	0.014	18	1	0.056
**Other herbaceous plants**						
Armeria sp.	71	1	0.014	18	1	0.056
Asparagaceae	71	1	0.014	18	1	0.056
Eryngium sp.	71	1	0.014	18	1	0.056
Fabaceae	71	2	0.028	18	2	0.111
Lamium amplexiculae	71	2	0.028	18	1	0.056
Muscari sp.	71	1	0.014	18	1	0.056
Phlomis sp.	71	1	0.014	18	1	0.056
Poaceae	71	1	0.014	18	1	0.056
Ranunculus sp.	71	1	0.014	18	1	0.056

Rumex sp.	71	7	0.099	18	4	0.222
**Other evidence**						
AOV	71	4	0.056	18	3	0.167

SI References

1. Madella, M., Powers-Jones, A. H. & Jones, M. K. A simple method of extraction of opal phytoliths from sediments using a non-toxic heavy liquid. *J. Archaeol. Sci.* **25**, 801–803 (1998).
2. Lancelotti, C. 'Not all that burns is wood'. A social perspective on fuel exploitation and use during the Indus urban period (2600-1900 BC). *PLOS ONE* **13**, e0192364 (2018).
3. Albert, R. & Weiner, S. Study of phytoliths in prehistoric ash layers from Kebara and Tabun Caves using a quantitative approach: 2nd International Meeting on Phytolith Research. *Phytoliths Appl. Earth Sci. Hum. Hist.* 251–266 (2001).
4. International Committee for Phytolith Taxonomy. International Code for Phytolith Nomenclature (ICPN) 2.0. *Ann. Bot.* **124**, 189–199 (2019).
5. Steffè, G. Le datazioni assolute. in *Il villaggio neolitico di Lugo di Romagna – Fornace Gattelli. Strutture, Ambiente, Culture* (eds Steffè, G. & Degasperi, N.) 279–288 (Istituto Italiano di Preistoria e Protostoria, Firenze, 2019).
6. Steffè, G., Degasperi, N., Dal Santo, N., Ferrari, A. & Rottoli, M. Il villaggio Fiorano di Lugo di Romagna: sintesi e inquadramento culturale. in *Il villaggio neolitico di Lugo di Romagna – Fornace Gattelli. Strutture, Ambiente, Culture* (eds Steffè, G. & Degasperi, N.) 580–608 (Istituto Italiano di Preistoria e Protostoria, Firenze, 2019).
7. XRONOS development team. Grotta dell'Edera. https://xronos.ch/sites/2034 (2022).
8. Skeates, R. & Whitehous, R. New radiocarbon dates for prehistoric Italy 3. *Accord. Res. Pap.* **7**, 149–162 (1999).
9. Skeates, R. New radiocarbon dates for prehistoric Italy, Supplementary list 5. *Accord. Res. Pap.* **9**, 163–181 (2004).
10. Pedrotti, A. Un insediamento d'altura alla Torretta di Isera (TN). in *Dalle radici della storia. Archeologia del Comun Comunale Lagarino. Storia e forme dell'insediamento dalla preistoria al Medioevo* (ed. Tecchiati, U.) 71–86 (Ed. Sella, Rovereto, 1996).
11. Pedrotti, A. *et al.* L'insediamento di Lugo di Grezzana (Verona) nel quadro del primo Neolitico padano alpino. in *Preistoria e protostoria del Veneto* vol. 1 95–107 (Istituto Italiano di Preistoria e Protostoria, Firenze, 2015).
12. Zanetti, A. L. & Tecchiati, U. I resti faunistici provenienti da una fossa della Cultura di Fiorano (Neolitico antico) scavata in località S. Andrea di Cologna Veneta (Verona). Dati preliminari. *Atti Dell8° Convegno Naz. Archeozoologia* **1**, 55–62 (2019).
13. Salzani, L., Padovan, S. & Salzani, P. Nuovo sito del Neolitico Antico a S. Andrea di Cologna Veneta (Verona). Nota preliminare. *Boll. Mus. Civ. Storia Nat. Verona Geol. Paleontol. Preistoria* **25**, 79–93 (2001).
14. Gobbo, I. Archeobotanica di siti mesolitici, neolitici ed eneolitici di pianura dell'Emilia Romagna. (Università degli Studi di Ferrara, 2011).
15. De Grossi Mazzorin, J. L'analisi archeozoologica di alcuni siti della cultura neolitica dei vasi a bocca quadrata del Parmense. *Riv. Studi Liguri* 87–94 (2014).
16. Dal Santo, N. & Mazzieri, P. La frequentazione di VBQ I nel sito pluristratificato di Benefizio: evidenze strutturali e cultura materiale. *Riv. Sci. Preistoriche* **66**, 25–60 (2016).
17. XRONOS development team. Bazzarola. https://xronos.ch/sites/1776 (2022).
18. Skeates, R. & Whitehouse, R. *Radiocarbon Dating and Italian Prehistory*. (1994).
19. Messana, C., Bernabò Brea, M., Bertolini, M. & Thun Hohenstein, U. Faunal exploitation in an Early Neolithic site: the assemblage from Casa Gazza (Travo, Piacenza, Northern Italy). in *Neolithic and Bronze Age studies in Europe. From material culture to territories* vol. 13 22–30 (Archaeopress Publishing LTD, Oxford, 2021).
20. Nicosia, C. *et al.* Nuovi scavi presso il sito del Neolitico Medio di Molino Casarotto nelle Valli di Fimon (Arcugnano, Vicenza). *Preistoria Alp.* **55**, 53–66 (2025).

21. Ferrari, A., Mengoli, D. & Steffè, G. L'abitato neolitico di Casalecchio di Reno, Zona "A" (Bologna). in *Preistoria dell'Italia settentrionale. Studi in ricordo di Bernardo Bagolini: atti del convegno, Udine, 23-24 settembre 2005* (eds Pessina, A. & Visentini, P.) 419–424 (Comune di Udine, Museo Friulano di Storia Naturale, 2006).
22. Mazzieri, P. I siti di via Spezia (Benefizio e via Guidorossi) e Pontetaro a Parma nel quadro della cultura dei vasi a bocca quadrata in Emilia occidentale. (University of Pisa, 2011).
23. Biagi, P., Starnini, E., Borić, D. & Mazzucco, N. Early Neolithic Settlement of the Po Plain (Northern Italy): Vhò and Related Sites. *Doc. Praehist.* **47**, 192–221 (2020).
24. Tirabassi, I. La fase iniziale della Cultura VBQ documentata a Rivaltella-Ca' Romensini (RE. *Riv. Studi Liguri* **LXXVII–LXXIX**, 429–438 (2014).
25. Pessina, A., Fontana, A., Rottoli, M., Occhini, E. & Salvador, S. Il Neolitico della Bassa Pianura Friulana. Aspetti culturali, geoarcheologici e paleobotanici. in *Preistoria e Protostoria del Caput Adriae* 135–146 (2018).
26. Banchieri, D. G., Bini, A., Rottoli, M. & Mainberger, M. Le Prealpi varesine e l'alimentazione durante la preistoria. in *Preistoria del cibo. L'alimentazione nella preistoria e nella protostoria* vol. 1 193–202 (2021).
27. Steiner, B. L., Antolín, F., Soteras, R., Rottoli, M. & Banchieri, D. G. Isolino Virginia (Lake Varese, Italy): New Archaeobotanical Research at the Earliest Pile-Dwelling of the Circumalpine Area. in *Prehistoric Wetland Sites of Southern Europe: Archaeology, Dendrochronology, Palaeoecology and Bioarchaeology* (eds Ballmer, A., Hafner, A. & Tinner, W.) 267–282 (Springer Nature Switzerland, Cham, 2025). doi:10.1007/978-3-031-52780-7_16.
28. Gilli, E. & Montagnari Kokelj, E. La Grotta degli Zingari nel Carso triestino (materiali dagli scavi 1961-1965). *Atti Della Soc. Preistoria E Protostoria Friuli-Venezia Giulia* **9 (1994-1995)**, 63–126 (1996).
29. Bagolini, B. & Biagi, P. The Radiocarbon Chronology of the Neolithic and Copper Age of Northern Italy. *Oxf. J. Archaeol.* **9**, 1–23 (1990).
30. XRONOS development team. La Vela. Xronos.ch (2022).
31. Cottini, M. & Rottoli, M. I carboni di legna e le piante coltivate. in *Bannia - Palazzine di Sopra. Una comunità preistorica del V millennio a.C.* (ed. Visentini, P.) 129–145 (2005).
32. Cazzella, A., Cremaschi, M., Moscoloni, M. & Sala, B. Siti neolitici in località Razza di Campegine (Reggio Emilia). *Preistoria Alp.* **12**, 61–70 (1976).
33. Bernabò Brea, M. *et al.* Lo scavo estensivo nel sito neolitico di Razza di Campeggine (Reggio Emilia). in *Archeologia ad alta velocità in Emilia. Indagini geologiche e archeologiche lungo il tracciato ferroviario* (eds Bernabò Brea, M. & Valloni, R.) 41–86 (All'Insegna del Giglio, Firenze, 2008).
34. Barfield, L. H. & Bagolini, B. The excavations on the Rocca di Rivoli Verona 1963-1968. *Mem. Mus. Civ. Storia Nat. Verona* **14**, 32–37 (1976).
35. Visentini, P. Aspetti cronologici e culturali della fine del Neolitico nell'Italia nord-orientale. in *Preistoria dell'Italia settentrionale. Studi in ricordo di Bernardino Bagolini Atti del Convegno, Udine 23-24 settembre 2005* (ed. Pessina, A.) vol. 53 225–241 (Museo Friulano Storia Naturale, Udine, 2006).
36. Bernabò Brea, M., Miari, M. & Steffè, G. Il Neolitico dell'Emilia Romagna. in *Preistoria e Protostoria dell'Emilia Romagna* vol. 1 119–137 (Firenze, 2017).
37. Dal Santo, N. & Mazzieri, P. Il sito di VBQ iniziale di Ponte Ghiara (Parma). Le industrie litiche e ceramiche. *Origini* **32**, 105–160 (2010).
38. Mottes, E. New evidence of settlement, burial and ritual aspects in the Neolithic and Copper Age in Trentino (northern Italy). in *Upiku: Tauke Festschrift für Gerhard Tomedi zum 65. Geburtstag* (eds Hye, S. & Töchterle, U.) 377–400 (R. Habelt, Bonn, 2019).

39. Mottes, E. Il neolitico dell'alto Garda: aspetti culturali e dinamiche insediative nel quadro della preistoria del territorio sudalpino centro-orientale. in *Vasi a Bocca Quadrata. Evoluzione delle conoscenze nuovi approcci interpretativi* (ed. Mottes, E.) 89–115 (2021).
40. XRONOS development team. Grotta del Mitreo. https://xronos.ch/sites/1274 (2022).
41. Petrucci, G. Resti di fauna dai livelli neolitici e post-neolitici della Grotta del Mitreo nel Carso di Trieste (scavi 1967). in *Atti della Società per la Preistoria e la Protostoria del Friuli-Venezia Giulia* vol. 10 99–118 (Edizioni Svevo, Trieste, 1996).
42. Panelli, C. Il sito neolitico di Vicofertile (Parma). *Riv. Studi Liguri* 439–446 (2014).
43. Castiglioni, E. I carboni di legna e i resti carpologici dall'insediamento Neolitico di Maserà di Padova, via Bolzani. in *Vasi a Bocca Quadrata. Evoluzione delle conoscenze nuovi approcci interpretativi* (ed. Mottes, E.) 249–255 (2021).
44. Bianchin Citton, E. Il neolitico dell'area euganea: un aggiornamento dei dati. in *Atti del convegno di studi, Este – 27 Novembre 2009, Monselice – 28 Novembre 2009* (eds Bianchin Citton, E., Rossi, S. & Zanovello, P.) 83–86 (Edizioni La Torre, Monselice, 2015).
45. Tecchiati, U. I resti faunistici del Neolitico recente (III fase VBQ) di Maserà e Monselice (Padova). in *Dinamiche insediative nel territorio dei Colli Euganei dal Paleolitico al Medioevo. Atti del convegno di studi, Este – 27 Novembre 2009, Monselice – 28 Novembre 2009* (eds Bianchin Citton, E., Rossi, S. & Zanovello, P.) 107–120 (Edizioni La Torre, Monselice, 2015).
46. Bernabò Brea, M., Maffi, M., Mazzieri, P. & Salvadei, L. Testimonianze funerarie della gente dei Vasi a Bocca Quadrata in Emilia occidentale. Archeologia e antropologia. *Riv. Sci. Preistoriche* **60**, 63–126 (2010).
47. Starnini, E., D'Amico, C., Biagi, P., Ghedini, M. & Pitti, G. Strumenti di pietra levigata dalla Lombardia Orientale: aspetti archeometrici e culturali. *Bull. Paletnologia Ital.* **95**, 23–81 (2004).
48. Dal Santo, N. & Mazzieri, P. Il sito del Neolitico recente di Botteghino (Parma). *Riv. Sci. Preistoriche* **57**, 113–138 (2007).
49. Bianchin Citton, E. & Ferrari, S. Il sito neolitico di Monselice - via Valli. in *Atti del convegno di studi, Este – 27 Novembre 2009, Monselice – 28 Novembre 2009* (eds Bianchin Citton, E., Rossi, S. & Zanovello, P.) 89–96 (Edizioni La Torre, Monselice, 2015).
50. Anghinelli, S. Stanziamento Neolitico della Cultura dei Vasi a Bocca Quadrata a Rivarolo Mantovano (MN), nel Campo «Pegorone III». *Preistoria Alp.* **20**, 81–102 (1984).
51. Agrostelli, M., Fontana, A. & Tecchiati, U. Castelnuovo di Teolo (Padova), scavi 2011. I dati archeobotanici e faunistici. in *Preistoria e protostoria del Veneto* vol. 1 159–168 (Istituto Italiano di Preistoria e Protostoria, Firenze, 2015).
52. Tiné, V., Mazzieri, P., Dal Santo, N. & Fuolega, F. Il villaggio neolitico del Dal Molin a Vicenza. in *Preistoria e Protostoria del Veneto* (eds Leonardi, G. & Tiné, V.) 73–94 (Istituto Italiano di Preistoria e Protostoria, Soprintendenza per i beni archeologici del Veneto, Università degli studi di Padova, Firenze, Padova, 2015).
53. Pini, R., Castellano, L., Perego, R., Ravazzi, C. & Rizzi, A. Un insediamento perilacustre del Tardo Neolitico - Età del Rame a Fimon Le Fratte. Successione sedimentaria, stratigrafia pollinica e macroresti vegetali. in *Nuove ricerche nelle Valli di Fimon. L'insediamento del tardo Neolitico de Le Fratte di Arcugnano* (ed. Bianchin Citton, E.) 169–194 (Editrice Veneta, Vicenza, 2016).
54. Castiglioni, E. & Rottoli, M. I resti carpologici dall'abitato di Tosina. in *Contadini, allevatori e artigiani a Tosina di Monzambano (Mn) tra V e IV millennio a.C. Una comunità neolitica nei circuiti padani e veneti* (ed. Poggiani Keller, R.) 157–166 (2014).
55. Carra, M. Per una storia della cerealicoltura in Italia settentrionale dal Neolitico all'Età del Ferro: strategie adattive e condizionamenti ambientali. (Alma Mater Studiorum - Università di Bologna, 2012).

56. Boschin, F. & Riedel, A. The late mesolithic and neolithic fauna of the Edera Cave (Aurisina, Trieste Karst): a preliminary report. in *Atti della Società per la Preistoria e la Protostoria del Friuli-Venezia Giulia* vol. 8 73–90 (Edizioni Svevo, Trieste, 2000).
57. Micheli, R. *et al.* Nuove ricerche al Palù di Livenza: lo scavo del Settore 3. in *Preistoria e Protostoria del Caput Adriae* 481–490 (2018).
58. *Bannia - Palazzine di Sopra. Una comunità preistorica del V millennio a.C.* (Comune di Pordenone Editore, Pordenone, 2005).
59. Guerreschi, A., Catalani, P. & Ceschin, N. Belforte di Gazzuolo (Mantova). Una stazione con vasi a bocca quadrata del Neolitico Superiore. *Preistoria Alp.* **22**, 35–118 (1986).
60. Bianchin Citton, E. & Pedrotti, A. Il neolitico recente di Cornuda: nota preliminare. in *Atti della XXVI Riunione Scientifica Il Neolitico in Italia* vol. 2 445–454 (Istituto Italiano di Preistoria e Protostoria, Firenze, 1987).
61. Salzani, L. Nogara. Scavo di pozzetti neolitici in località Olmo. *Quad. Archeol. Veneto* **11**, 53–55 (1995).
62. Frezza, A. M. Studio morfologico e morfometrico anatomo-scheletrico di Pagellus erythrinus ed Esox lucius attuali per l'interpretazione archeozoologica di resti ittiologici. (Department of Comparative and Evolutionary Biology, University of Naples Federico II, 1997).
63. Jelu, I., Wouters, W. & Van Neer, W. The use of vertebral measurements for body length and weight reconstruction of pike (Esox lucius) from archaeological sites. *Archaeol. Anthropol. Sci.* **13**, 72 (2021).
